# Excessive DNA Double‐Strand Breaks–Associated 3D Genome Reorganization Contributes to Neural Tube Defects with Folate Deficiency

**DOI:** 10.1002/advs.202410603

**Published:** 2025-09-18

**Authors:** Ting Zhang, Lin Lin, Jianting Li, Caihua Li, Shengjun Liang, Xuemei Bai, Fang Wang, Yihua Bao, Dan Guo, Xiaochen Bo, Hao Li, Hebing Chen, Qiu Xie

**Affiliations:** ^1^ Capital Center for Children's Health Capital Medical University Capital Institute of Pediatrics Beijing 100020 China; ^2^ Institute of Health Service and Transfusion Medicine Beijing 100850 China; ^3^ School of Computer Science and Information Technology& KLAS Northeast Normal University Beijing 100730 China; ^4^ Department of Biochemistry and Molecular Biology College of Basic Medicine Shanxi Key Laboratory of Birth Defect and Cell Regeneration MOE Key Laboratory of Coal Environmental Pathogenicity and Prevention Taiyuan 030001 China; ^5^ Shanghai GeneCowin Biotechnology Co. Ltd. Shanghai 200120 China; ^6^ Department of Children's and Adolescent Health Public Health College Harbin Medical University Harbin 150081 China; ^7^ Center for Biomedical Technology Institute of Clinical Medicine Peking Union Medical College Hospital Chinese Academy of Medical Sciences Beijing 100730 China; ^8^ State Key Laboratory of Complex Severe and Rare Diseases Peking Union Medical College Hospital Chinese Academy of Medical Sciences and Peking Union Medical College Beijing 100850 China

**Keywords:** 3D genome, DNA double‐strand breaks, folic acid, high‐throughput chromosome conformation, neural tube defects

## Abstract

Neural tube defects (NTDs) are one of the most common congenital malformations. Folic acid deficiency in pregnant women increases the risk of developing NTDs; however, the underlying etiology and mechanisms remain elusive. In this study, the role of DNA double‐strand breaks (DSBs) in 3D genome organization in NTDs with folate deficiency is reported. The NTD mouse model is burdened with abundant DSBs associated with the disruption of 3D genome organization. DSBs occurring in active genes lead to the stalling of RNA polymerase II (Pol II) and formation of R‐loops in the 3D genome. The DSB ratios of the genomic regions negatively correlated with the distance from the transcription start sites of the gene. The DSB ratios of the proximal and distal enhancers are significantly higher and induce the displacement of loops with busy anchors. Furthermore, DSB‐associated dysregulation of chromatin loops occurs in neural tube closure–associated genes that are abnormally expressed in human NTDs. Taken together, excessive DSB‐associated 3D genome organization disruption within NTDs with folate deficiency contributes to the dysregulation of neural tube closure–associated genes.

## Graphical abstract

Neural tube defects (NTDs) are among the most common congenital malformations. However, the underlying etiology and mechanism remain elusive. Here, the role of DNA double‐strand breaks (DSBs) in 3D genome organization within the NTDs with folate deficiency is reported. DSB‐associated dysregulations of chromatin loops exist on neural tube closure‐associated genes that are abnormally expressed in human NTDs.

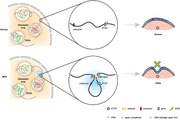

## 1 Introduction

Neural tube defects (NTDs) are severe birth defects reported worldwide. The etiology is the failure of neural tube closure during the first trimester. NTDs are among the leading causes of abortion, infant death, and lifelong disability in children. Worldwide, the incidence of NTDs is 1.86%; in northern China, it is 13.9%.^[^ ^1^ ^]^ NTDs are complex diseases with multiple etiologies, levels, and mechanisms. Methylation is one of the most important epigenetic modifications that is crucial for neurodevelopment. Pre‐pregnancy folate supplementation can prevent 70% of NTDs; however, the underlying etiology and mechanism remain elusive.^[^ ^1^ ^]^

Recent technical advances, such as chromosome conformation capture analysis, which detects the DNA–DNA interactions between close genomic loci within the 3D space of the nucleus, have provided new insights into the spatial organization of chromatin. High‐throughput chromosome conformation capture (Hi‐C) has been used to reveal the landscape of 3D structures.^[^ ^2^ ^]^ These hierarchical structures are essential for controlling gene expression, and disturbances in these structures have been implicated in various human disorders. Although traditionally investigated in cancer cell lines, recent studies have begun to profile 3D genomes of the nervous system to address their functions and mechanisms of action.^[^ ^3^ ^]^ Previous studies have generated high‐resolution Hi‐C maps of mouse neural development, delineating the relationship between transcription and topologically associated domains (TADs), specifically alterations in zinc‐finger binding protein CCCTC‐binding factor (CTCF)‐mediated TADs and loops throughout neural development.^[^ ^4^ ^]^ The deployment of low‐input “easy Hi‐C” in human brain tissues and neural cells has enabled to detect chromatin loops to better elucidate neurological genome‐wide association studies (GWAS), thus linking chromatin structural changes with neurodegenerative diseases.^[^ ^5^ ^]^

DNA double‐strand breaks (DSBs) are among the most severe types of DNA damage that markedly affect genomic stability and are critical for cellular function.^[^ ^6^ ^]^ A nonrandom distribution of DNA damage in the neuronal genome has been discovered, notably in neuronal enhancers, which appear to be hotspots for single‐stranded breaks. This pattern is presumably the result of multiple cycles of enhancer cytosine methylation and demethylation.^[^ ^7^ ^]^ DSBs have the potential to directly affect the 3D genome organization.^[^ ^8^ ^]^ Persistent DSBs in neurons can indirectly affect 3D genome organization through genome structural variations (SVs).^[^ ^9^ ^]^ Furthermore, disruption of genome stability and 3D genome organization by the DSBs in neurons is a pathological step in the progression of neurodegenerative diseases.^[^ ^10^ ^]^ Folate serves as a source of epigenetic modifications that affect the methylation of DNA, proteins, and lipids via a one‐carbon transfer reaction.^[^ ^11^ ^]^ Disrupted one‐carbon metabolism results in increased genome instability and chromosomal breakage due to excessive deoxyuridine monophosphate (dUMP) accumulation, which interferes with DNA synthesis. Folate deficiency causes massive incorporation of uracil into human DNA (4 million per cell) and chromosome breaks, which are implicated in neuronal damage during early embryonic development.^[^ ^12^ ^]^ However, the potential associations between DSB‐mediated genomic alterations and susceptibility to NTDs remain largely unexplored.

Current sequencing technologies have enabled genome‐wide mapping of DSBs at base‐pair (bp) resolution.^[^ ^13^ ^]^ In the present study, using a DSB enrichment workflow established at bp resolution,^[^ ^14^ ^]^ we analyzed the potential role of DSBs in active gene leading to RNA polymerase II (Pol II) stalling and R‐loop formation in the 3D genome regulation, which elicits NTD phenotypes with folate deficiency. We revealed that NTD mice burdened with DSBs were associated with disruption of 3D genome organization. DSB‐associated dysregulation of chromatin loops occurred in neural tube closure–associated genes that are abnormally expressed in human NTDs. Taken together, excessive DSB‐associated 3D genome organization disruption within NTDs with folate deficiency contributes to the dysregulation of neural tube closure–associated genes.

## 2 Results

### 2.1 Folate Deficiency–Induced NTD Mice Burdened with DSBs are Associated with Disruption of 3D Genome Organization

**Figure 1:**
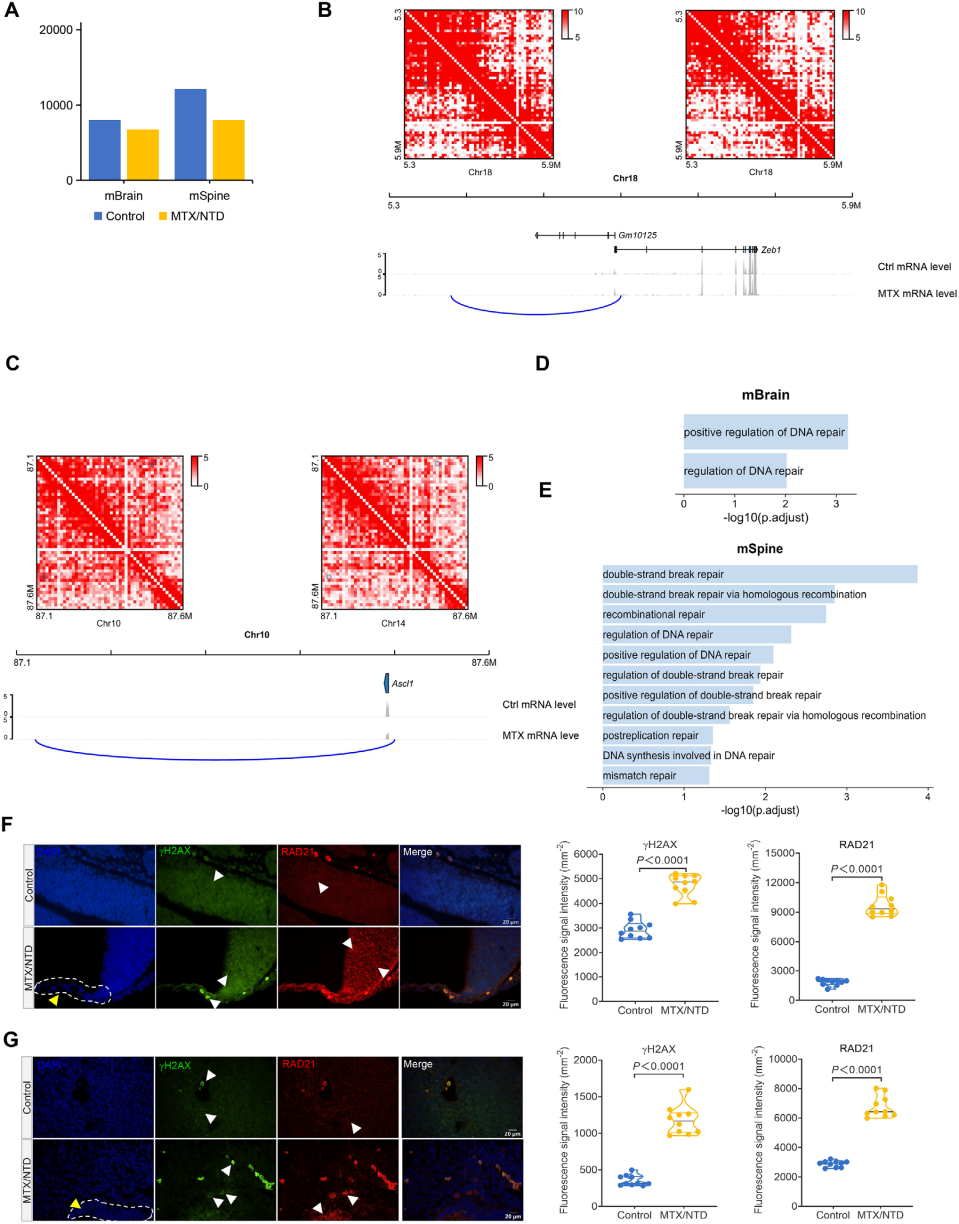
MTX‐induced NTD mice burdened with DSBs are associated with 3D genome organization disruption. A) Bar plot illustrating chromatin loop counts derived from three sets of mouse Hi‐C data. B) Track maps presenting the association between chromatin loop shifts and gene expression in the mouse brain. Gene expression (gray) from control and treatment groups is shown, with emphasis on *Gm10125 and Zeb1* aligning with chromatin loop alterations. C) Track maps presenting the association between chromatin loop shifts and gene expression in mouse spinal tissue. Gene expression (gray) from control and treatment groups is shown, with emphasis on *Ascl1* aligning with chromatin loop alterations. D) GO analysis of genes located in differential loops for mBrain. E) GO analysis of genes located in differential loops for mSpine. F) Representative images of NTD mouse brain tissues with gH2AX and RAD21 foci by immunofluorescent staining gH2AX (panel 2) and RAD21(panel 3)(*n* = 10). Nuclear DNA was stained by DAPI (panel 1). Merged images (panel 4) are shown. Green: gH2AX; Red: RAD21; blue: DAPI staining. White dashed line represents the abnormal area of the nervous system. Bar, 20 µm. Data represent mean ± SEM from *n* = 3 biological replicate. Statistical significance was determined by the Wilcoxon test (**p < 0.01, ***p < 0.001, ****p < 0.0001). G) Representative images of NTD mouse brain tissues with gH2AX and RAD21foci by immunofluorescent staining gH2AX (panel 2) and RAD21(panel 3) (*n* = 10). Nuclear DNA was stained by DAPI (panel 1). Merged images (panel 4) are shown. Green: gH2AX; Red: RAD21; blue: DAPI staining. White dashed line represents the abnormal area of the nervous system. Bar, 20 µm. Data represent mean ± SEM from *n* = 3 biological replicate. Statistical significance was determined by the Wilcoxon test (**p < 0.01, ***p < 0.001, ****p < 0.0001).

The topological chromatin domains from human and mouse cells showed very similar TAD structures in syntenic regions, indicating that mice could serve as a suitable model system for studying 3D genome alterations in NTDs.^[^ ^15^ ^]^ To investigate how folate deficiency comprehensively regulates the 3D genome structure in NTD, we established an NTD mouse model using methotrexate (MTX) as previously reported.^[^ ^16^ ^]^ MTX is a potent analog belonging to a class of specific inhibitors of dihydrofolate reductase (DHFR) that prevents the conversion of folate to its active form. MTX disrupts the one‐carbon metabolism by competitively inhibiting DHFR activity. The intact morphology of normal mouse embryos and the morphology of NTD mouse embryos at day 9.5, during the stage of neural tube closure, exhibited classical spine bifida and anencephaly phenotypes (Figure S1A, Supporting Information). We measured the tissue concentrations of 5‐methyltetrahydrofolate, 5‐formyltetrahydrofolate, and folic acid in NTD models, as reported previously.^[^ ^17^ ^]^ We collected brain (mBrain) and spinal (mSpine) tissues from 9.5‐day embryos for RNA sequencing (RNA‐seq) and Hi‐C (Figure S1B,C, Supporting Information). Gene Set Enrichment Analysis (GSEA) of differentially expressed genes (DEGs) from RNA‐seq revealed that the differential genes were primarily associated with regionalization, Pol II transcription regulatory region sequence‐specific DNA binding, and developmental processes (Figure S1D,E, Supporting Information). Next, to examine the specific effects of 3D structural changes induced by NTDs, we identified chromatin loops in NTD brain and spine samples using Hi‐C with Peakachu to calculate the whole genome interaction with a resolution of 10 kb and a quality‐controlled threshold value of 0.95 to detect loop calling (**Figure** **1**A). A differential loop refers to a loop that is specifically present in either the MTX‐treated or control groups, indicating chromatin loops that are gained or lost upon MTX treatment. By comparing the NTD and control groups, we evaluated the differential loop specificity using Aggregate Peak Analysis (APA) and calculated the number of differential loops. The results showed a loss of 3430 loops and a gain of 2382 loops in NTD brains (Figure S1F, Supporting Information). In addition, the examination of NTD mouse spinal tissues demonstrated a notable loss of 4645 loops, in contrast to the gain of 1783 loops (Figure S1G, Supporting Information). The Chromosome/Chromatin Conformation Capture (3C) assay confirmed loop changes, including loss (Figure S2A,C,E and Table S1, Supporting Information) and gain loops (Figure S2B,D,F and Table S2, Supporting Information). Furthermore, the chromatin loop alterations consistently occurred within identical genomic regions between mouse embryonic stem cells (mESCs) and NTD tissues (Figure S3A–F and Table S3, Supporting Information). These results suggest that persistent loop changes may occur during neural tube closure in MTX‐induced NTD mice.

Chromatin 3D structure can regulate gene expression. We investigated whether gene transcription in mouse NTD tissues was influenced by chromatin loop structure. Interestingly, we found that genes encoding the transcription factors *Zeb1* and *Ascl1* were both involved in chromatin loop changes. *Zeb1*, one of the 562 neural tube closure–associated genes reported in previous studies^[^ ^18^ ^]^ (Table S4, Supporting Information), regulates the orientation of the cleavage plane of dividing neural progenitors, neuronal polarity and migration, and promotes nonhomologous end‐joining DSB repair.^[^ ^19^ ^]^ The results showed that alterations in *Zeb1* transcriptional activity coincided with the changes observed in the chromatin loops (Figure 1B). Furthermore, *Ascl1*, which induces the generation of functional dopaminergic neurons from mouse fibroblasts, actively participates in the activity of neural stem and progenitor cells,^[^ ^20^ ^]^ and its expression is significantly decreased under NTDs, accompanied by loss of chromatin loops (Figure 1C). RT‐qPCR and western blotting experiments confirmed the aberrant expression of *Zeb1* and *Ascl1* in the cell models and NTD tissues (*p*‐value < 0.05, *t*‐test; Figure S4A–D and Table S3, Supporting Information). In addition, the same changes in the immunohistochemistry of *Zeb1* and *Ascl1* were also observed in NTD mouse brains (*p*‐value < 0.0001, *t*‐test; Figure S4E, Supporting Information). To further confirm whether chromatin loop changes were a direct reason for the changes in the expression levels of *Zeb1* and *Ascl1*, we used CRISPR/Cas9 gene editing technology to delete the genomic regions by anchoring the enhancer loop to both the *Zeb1* and *Ascl1* loci (Figure S5A,D and Table S5, Supporting Information). We confirmed the homozygous deletion in a single‐cell–derived clone using Sanger sequencing (Figure S5B,E and Table S6, Supporting Information). RT‐qPCR was performed using single‐cell clones from the target gRNAs of *Zeb1* and *Ascl1*. *Zeb1* was expressed at higher levels than in the control group (*p*‐value = 0.004, *t*‐test; Figure S5C, Supporting Information), whereas *Ascl1* was expressed at lower levels than in the single‐cell clones (*p*‐value = 0.04, *t*‐test; Figure S5F, Supporting Information). These results demonstrated that chromatin loop changes play a critical role in *Zeb1* and *Ascl1* expression.

Next, we performed Gene Ontology (GO) pathway analysis of the genes located in the differential loops of mBrain and mSpine. Notably, the DNA repair pathway was significantly enriched (*p*.adjust < 0.05; Figure 1D,E), indicating that DNA damage and defective DNA repair occurred during NTD progression. Loop extrusion is mediated by cohesion, and CTCF mediates the formation of chromatin loops and self‐interacting TADs.^[^ ^21^ ^]^ The cohesion complex is an important architectural protein that organizes the 3D genome and is necessary for DSB repair.^[^ ^22^ ^]^ To investigate the relationship between chromatin loops and DSBs during neural tube closure, we examined the expression of the cohesion subunit RAD21 and the DNA damage marker gH2AX in E9.5 NTD embryos using immunofluorescence staining. Our results indicated that RAD21 was significantly enriched in the abnormal nervous system with DSBs in both the mBrain and mSpine of NTD mice compared with baseline DSBs in the control group (*p‐*value < 0.0001, *t*‐test; Figure 1F,G), indicating that folate deficiency–induced NTD mice burdened with DSBs were associated with disruption of 3D genome organization.

### 2.2 DSBs Occurring in Active Genes Lead to Pol II Stalling and R‐Loop Formation in the 3D Genome with Folate Deficiency

R‐loops can serve as intermediates through Pol II stalling in DNA damage repair processes, and abnormal accumulation of R‐loops can impede replication fork progression.^[^ ^8d^ ^]^ When considering that chromatin is organized into loops and self‐interacting units called TADs by cohesion and CTCF, how DSBs affect loop and TAD organization under folate deficiency remains unknown. The chromatin conformation of ESCs was consistent with that of developing tissue.^[^ ^15a^ ^]^ We have previously identified a DSB landscape with DSB enrichment workflow in mESCs using MTX, which has been implicated as a risk factor for NTDs.^[^ ^14^ ^]^ mESCs were treated with 0.12 µm MTX for 24 h and intracellular folate levels were measured. The expression in the MTX‐treated group was found to be significantly lower than that in the untreated mESC (*p‐*value = 0.017, *t*‐test; Figure S6A, Supporting Information). To screen whether neural tube closure–associated genes were differentially expressed, we performed RNA‐seq. We observed that DEG mainly focused on tissue development, cell growth, and DNA damage response (Figure S6B, Supporting Information). It is worth noting that neural tube closure–associated genes are mostly differentially expressed in both mESCs and tissues, suggesting that mESCs treated with MTX can be used as a cell model for NTD analysis.

Next, we performed high‐resolution Hi‐C sequencing of the normal and MTX‐treated mESC. The A/B compartment comparison showed that the fundamental structural characteristics of the 3D genome were mostly unchanged after MTX treatment, consistent with observations in mouse NTDs (Figure S6C, Supporting Information). In mESCs, A/B compartments affected by MTX treatment accounted for a small proportion (<5%) of the entire genome (Figure S6D, Supporting Information). Based on changes in the A/B compartment, we divided the genome into four types: stable A, A‐to‐B, B‐to‐A, and stable B regions. A/B compartment dynamics between mESCs and tissues revealed that a large majority of the genomic regions in the mouse tissues were preserved from the embryonic phase (Figure S6E, Supporting Information). Interestingly, by examining gene expression, we found that DEGs were significantly enriched in the stable A region compared to the other regions (Figure S6F, Supporting Information).

**Figure 2:**
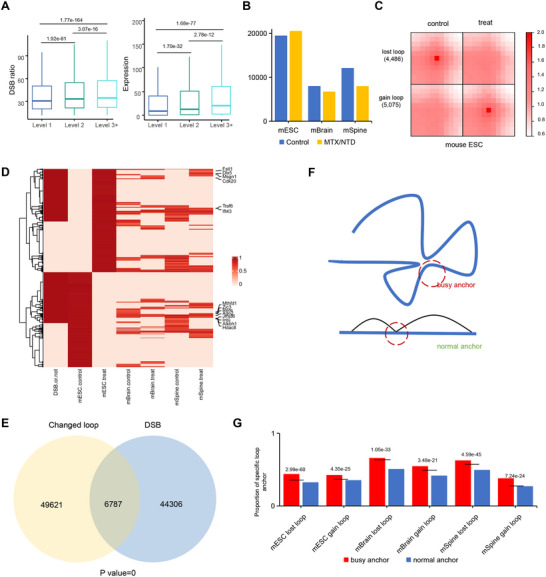
MTX‐treated mECSs induce displacement of loops with busy anchors. A) Box plot indicating the correlation between DSB signal and gene expression within hierarchical TADs. Significance values as follows: 1.92e‐81, 3.07e‐16, 1.77e‐164(left panel); 1.70e‐32, 2.78e‐12, and 1.68e‐77 (right panel). Statistical significance was determined by the Wilcox test. B) Bar plot illustrating chromatin loop counts derived from three sets of mouse Hi‐C data. C) APA analysis validating the designation of specific loops. D) Heatmap illustrating the conserved patterns (gain or loss) of specific loops (3361) in mouse embryonic stem cells, brain, or spine after MTX treatment. Whether specific loops occur DSB has been labeled. E) Venn diagram shows the overlap between DSB located in changed loop achors and the DSB of the whole genome (Chi‐square test calculates significance). F) Schematic representation of the “busy anchor” and “normal achor.” G) Bar plot detailing the prevalence of specific chromatin loop anchors categorized by anchor activity (BUSY or NO). Busy anchors more readily form specific loops, with significance values as follows: 2.01e‐28, 5.38e‐12, 1.03e‐10, 9.91e‐09, 1.65e‐14, and 3.80e‐13. Statistical significance was determined by the chi‐square test.

Subsequently, we examined the relationship between DSB ratios and gene expression in MTX‐treated and control samples at the TAD level. Higher levels of TADs were associated with an increased expression of active genes (Figure S6G, Supporting Information). As the hierarchical level of the TADs increased, both the DSB ratio and gene transcriptional activity gradually increased. A clear correlation was observed among gene transcription, DSBs, and TAB levels when analyzed in the context of TADs (**Figure** **2**A; Figure S6H, Supporting Information).

Indeed, we showed that increased TAD was associated with the DSB ratio as well as transcriptional activity with MTX treatment. We then identified chromatin loops in mESCs treated with Peakachu, which resulted in the loss of 4486 loops and gain of 5075 loops after MTX treatment (Figure 2B,C). Chromatin loop changes in mESCs, mouse brains, and spines with or without MTX treatment, as well as the conservation of these changes in mESCs and mouse tissues, were compared. Only a small subset of chromatin loop changes were conserved in both mESCs and mouse tissues (Figure 2D). Intriguingly, most neural tube closure–associated genes were located in the regions of these conserved and altered chromatin loops, making MTX‐treated mESC a convincing model for studying NTDs.

In parallel, we identified 6787 DSB overlapping with differential loop anchors in mESC treated with MTX. This indicated that the changed loops were more likely to be enriched in the DSB region (Figure 2E). Loop anchors that recurred more than once were designated as “busy anchors,” acting as hubs for chromatin interactions, whereas the others were classified as “normal anchors” (Figure 2F). A comparative analysis revealed that the displacement of the loop with a busy anchor was more significant than that of the loop with a normal anchor after MTX treatment in terms of the prevalence of specific chromatin loop anchors categorized by anchor activity (Figure 2G). This supports our hypothesis that the chromosomal regions with a high frequency of chromatin contact are more prone to folate deficiency–induced disruption, thus leading to significant changes in chromatin loops.

### 2.3 Characteristics of Folate Deficiency–Induced DSBs in Genome

Because DSBs are associated with the 3D genome signature for neural tube development, we wondered whether regions with DSBs are susceptible to forming a new loop for gene dysregulation. To invalidate this, we first mapped DSBs directly using Break Labeling in situ and sequenced mESC with MTX. The level of DSB enrichment increased after MTX treatment, consistent with that observed in folate deficiency–induced NTD mice. The presence and nature of histone marks have classically been used to identify epigenetic features such as enhancers, promoters, and open and closed chromatin. To determine the distribution of DSBs induced in mESCs, we performed ChIP‐seq in mESCs treated with MTX to classify chromatin states using ChromHMM^[^ ^23^ ^]^ (Figure S7A, Supporting Information). Genomic regions were categorized into 10 distinct chromatin states based on the combinatorial patterns of the selected chromatin marks (Figure S7B, Supporting Information). Two states marked as low chromatin signals were excluded from the downstream analysis. Distance analysis revealed that the genomic regions marked as transcription start sites (TSSs) including TssA, TssWk1, and TssWk2, were very close to the TSSs (Figure S7C, Supporting Information). To better understand the role of histone modification with DSB in chromatin, ATAC‐seq experiments were performed in both normal and MTX‐treated mESCs (Figure S7D, Supporting Information). The results showed that a large proportion of ATAC‐seq peaks in normal mESCs overlapped with DNase I‐hypersensitive sites (DHSs) of mESCs from the ENCODE project (Figure S7E, Supporting Information). In addition, distinct ATAC‐seq signals were detected around the TSSs (Figure S7F, Supporting Information).

**Figure 3:**
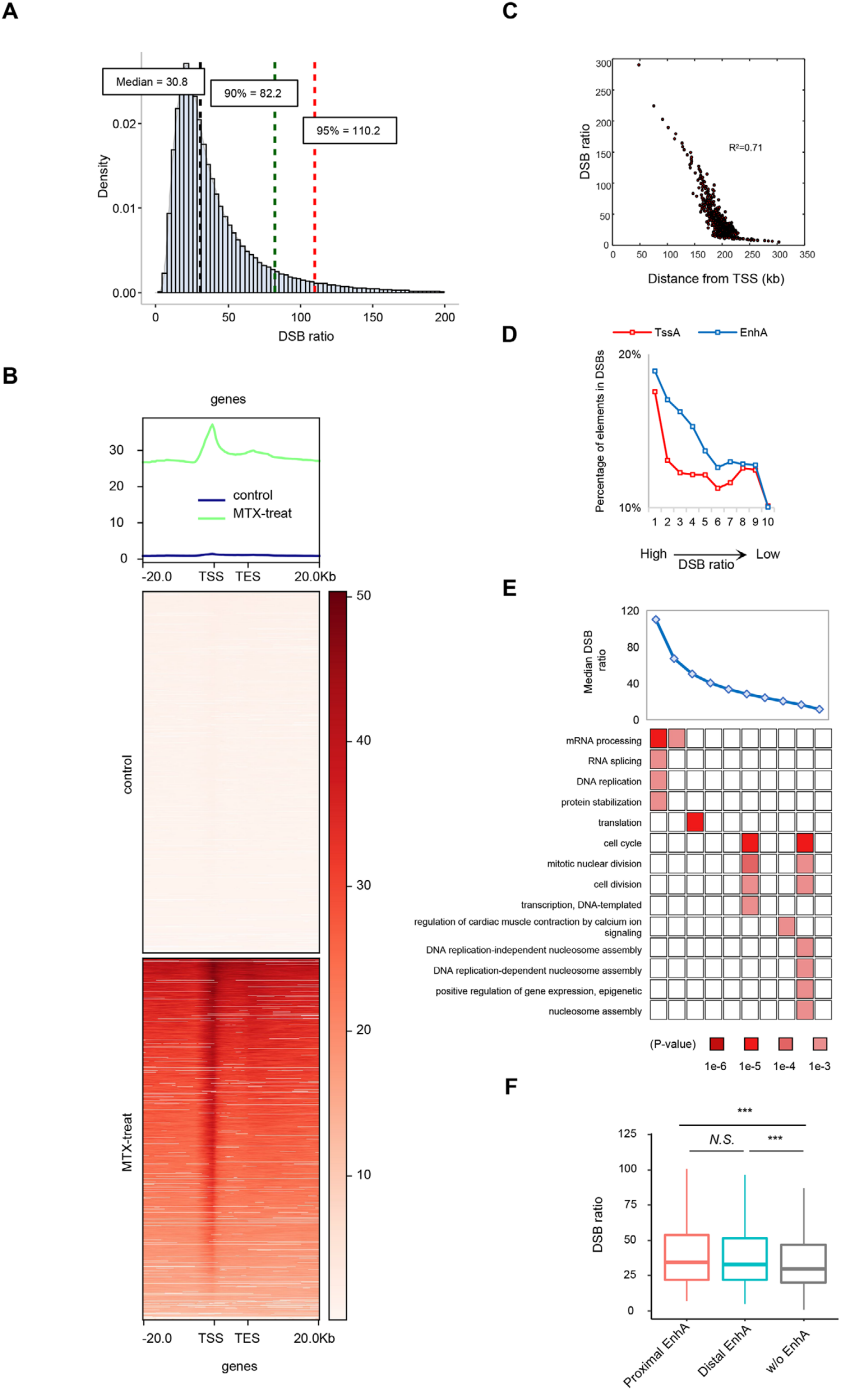
MTX‐induced DSBs accumulate at promoters and enhancers. A) Bar plot showing the distribution of DSB ratios at 5 kb resolution. B) The average signal profile depicts the distribution of DSB enrichment near TSS, reflecting a significant increase in DSB enrichment after MTX treatment. C) Scatter plot showing the correlation between the DSB ratio and the distance from gene TSS. Genomic regions were sorted and split into 500 bins in descending order of the DSB ratio. Distance from TSS was calculated as the length between gene TSS and the median nucleotide of each genomic region. D) Genomic regions were split into ten classes according to the DSB ratio. The percentages of genomic regions with an active TSS (red) and an active enhancer (blue) were positively correlated with DSB ratio. E) Heatmap showing the biological processes particularly associated with each class of DSB‐associated genes. F) Boxplot showing the DSB ratios of proximal EnhAs (red), distal EnhAs (blue), and genomic regions without EnhAs (gray). Data represent mean ± SEM from *n* = 3 biological replicate. Statistical significance was determined by unpaired two‐tailed Student's *t*‐test (***p < 0.001, N.S. represents no significance).

To eliminate the effects of endogenous DSBs, we calculated the DSB ratios as the fold change in DSB enrichment in MTX‐treated and normal mESCs for each genomic region. The results showed that mESCs underwent a marked increase in DSB enrichment after MTX treatment, with an average ≈28.5‐fold increase across the genome (**Figure** **3**A). Moreover, the enrichment of DSBs induced near the TSS was higher than that in the intergenic regions and other areas (Figure 3B). The DSB ratios of the genomic regions were negatively correlated with the distance from the TSS of the gene, and the DSB ratio was extremely high near the TSS (Figure 3C).

Proper functioning of gene promoters and enhancers is essential for coordinated transcription within cells.^[^ ^24^ ^]^ Next, we focused on the DNA breaks at these locations after MTX treatment. The numbers of TssAs and EnhAs in ten different chromatin states were calculated. The performed correlation analysis revealed that the proportions of TssAs and EnhAs located in the genomic regions positively correlated with the DSB ratio of these regions (Figure 3D). We also identified TssA‐associated genes in each class of DSB‐split regions and performed GO analysis using DAVID.^[^ ^25^ ^]^ TssA‐associated genes located in genomic regions with the highest DSB ratios are related to basic biological functions, such as mRNA processing, RNA splicing, and DNA replication. These genes were also associated with DNA repair and the cellular response to DNA damage stimulus functions (Figure 3E), indicating that fragile active promoters affect fundamental processes in the cell and may also be active in DNA repair. GO analysis of 2291 active enhancers with the highest DSB ratios using GREAT^[^ ^26^ ^]^ showed that 1837 genes were associated with these active enhancers and biological processes, such as cytoskeleton‐dependent intracellular transport, cellular response to leukemia inhibitory factor, and cell–substrate adhesion. Next, we identified proximal EnhAs as those with nearby TssAs located within 20 kb upstream or downstream, and distal EnhAs as those lacking TssAs within such a region. The performed comparative analysis indicated that the DSB ratios of the proximal and distal EnhAs were significantly higher than those in regions without EnhAs (*p*‐value < 0.001, *t*‐test; Figure 3F).

Repetitive sequences are composed of transposable elements and simple repeats and are widely distributed in the genomes of mice and humans.^[^ ^27^ ^]^ In our previous study, the hypomethylation of long interspersed nucleotide element‐1 (LINE1 or L1) and genomic DNA was associated with an increased risk of NTDs.^[^ ^8c^ ^]^ We found that the distribution of MTX‐induced DSBs correlated with different repeat subtypes of repetitive elements (Figure S7G, Supporting Information). DSBs were positively correlated with long interspersed nucleotide element‐2 (LINE2 or L2) repeats and negatively correlated with L1 repeats. DSBs positively correlated with multiple repeats within SINEs and low‐complexity repetitive sequences. For long terminal repeats (LTRs), DSBs were positively correlated with gypsy repeats and inversely correlated with ERV1 and ERVK repeats. Additionally, the comparative analysis revealed that the DSB enrichment at genomic regions harboring ATAC‐seq or ChIP‐seq peaks (H3K4me1, H3K4me3, H3K27ac, and H3K27me3) was significantly higher than that seen for those without peaks for both MTX‐treated mESCs (*p*‐value < 3 × 10^−23^, *t*‐test) and normal mESCs (*p*‐value < 0.008, *t*‐test; Figure S7H, Supporting Information). These results suggest that DSBs occur preferentially in epigenetic modification–enriched regions during folate deficiency.

### 2.4 DSB‐Associated Dysregulation of Chromatin Loop Occurs in Neural Tube Closure–Associated Genes

To determine whether DSB‐associated chromatin loop dysregulation influences gene expression, we analyzed the overlap of altered loop anchors and differential genes in mESC. A total of 43 differential genes were enriched in the differential loop anchors (**Figure** **4**A). We then performed GO pathway analysis of 43 genes and found that they were significantly enriched in the neural tube development pathways (*p*.adjust < 0.05; Figure 4B; Figure S8A,B, Supporting Information). Specifically, *Hoxa1* and *Rgma* regulate oriented cell division, cell shape, and microtubule dynamics during neural tube morphogenesis. *Sox6* and *Nrg1* regulate dorsal progenitor identity and interneuron diversity during neocortical development. *Baiap2*, *Plk2*, and *Nrg1* are related to dendritic spine development, whereas *Rgma* and *Nrg1* are enriched in the regulation of axon regeneration. Furthermore, *Plk5*, *Baiap2*, *Rgma*, and *Nrg1* may participate in the positive regulation of neuron projection development. *Rgma* and *Nrg1* also play important roles in regulating neuronal projection regeneration. In addition, *Baiap2*, *Fgfbp3*, and *Nrg1* regulate the nervous system development.

Figure 4DSB‐associated dysregulation of chromatin loop aligns with the differential gene expression. A) Venn diagram shows the number of differential genes enriched in changed loops achors. B) GO analysis of differential genes enriched in changed loops achors. C) CRISPR/Cas9 gene editing was utilized to delete a differential loop in the *Sox6* locus nearby DSB hotspot. A combination of two different gRNAs cloned into vectors conveying puromycin resistance to allow for double selection after the lentiviral transduction of sv129 mESC. One single cell clones obtained from gRNA combinations carrying the homozygous deletion and the same single cell clones that underwent no CRISPR system treatment. Track map elucidating the relationship between chromatin loop modifications and gene expression in mouse embryonic stem cells and NTD mouse brain. Annotations: CTCF (orange), H3K27me3 (green), ATAC‐seq (purple), and gene expression (gray). Chromatin loop gains (red) and losses (blue) are depicted, highlighting loop anchors and exons. Differences between MTX‐treated and control groups reveal expression changes in *Sox6* potentially attributed to chromatin loop and histone signal variations. D) Track maps presenting the association between chromatin loop shifts and gene expression in mouse brain and spinal tissue cells. Gene expression (gray) from control and treatment groups is shown, with emphasis on *Gm10125, Zeb1*, and *Ascl1* aligning with chromatin loop alterations. E) Bar plot presenting chromatin structural variations across three mouse Hi‐C datasets, analyzed using EagleC. F) Circos maps illustrating chromosomal translocations in mouse ESC. MTX treatment escalates the frequency of these translocations. G) Chromatin interaction heatmap visualizing structural variation patterns. Overlaying tracks highlight signals for DSB (black), RNA‐seq (control: yellow; MTX treatment: orange), and RNA ratio (red, control/MTX treatment). A co‐localization between inversion break and DSB signal may directly modulate *Dpp6* gene expression. H) Chromatin interaction heatmap detailing structural variation patterns in mouse spine tissue. Corresponding tracks depict DSB (black), RNA‐seq (control: dark green; MTX treatment: light green), and RNA ratio (orange, control/MTX treatment). Expression alterations in the *Gm20707* genes could be linked to nearby inversions.

**Figure:**
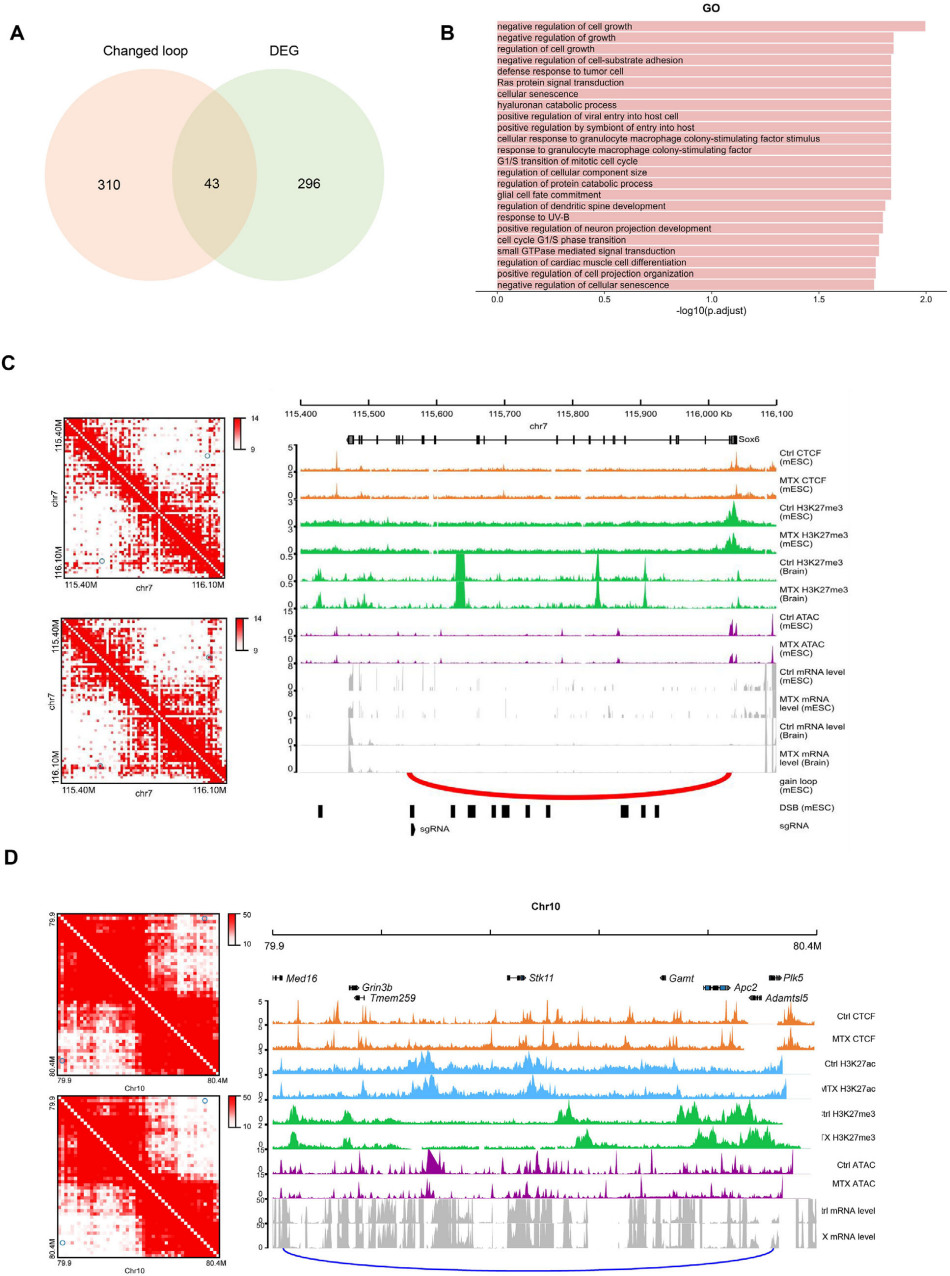

**Figure:**
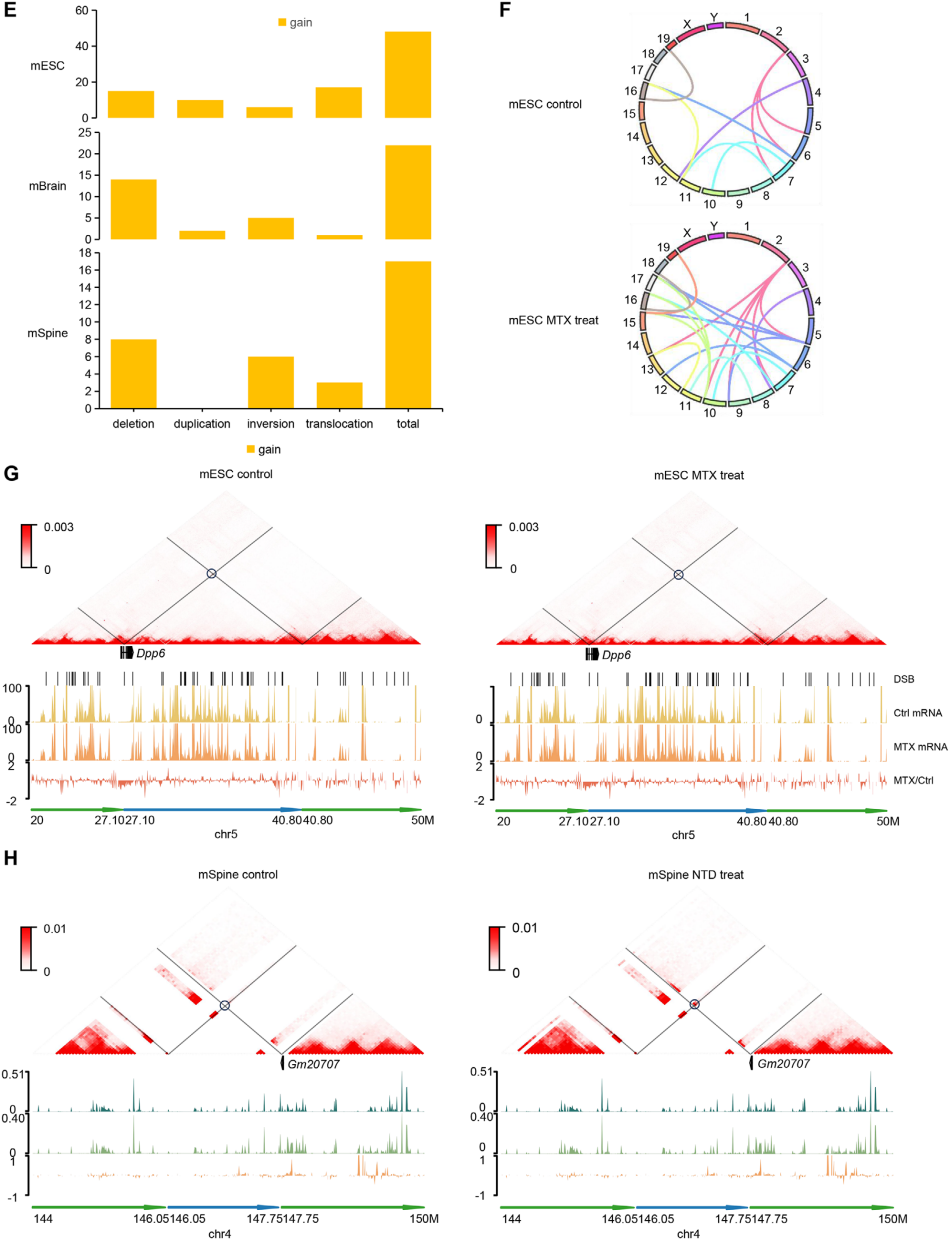

To better understand the relationship between the function of the chromatin loop and gene expression, we examined the histone markers located at the intersections of chromatin loops in mESCs. *Sox6*, an important gene encoding a transcription factor, plays a crucial role in the development of the central nervous system.^[^ ^28^ ^]^ The results revealed *Sox6* downregulation in the NTDs and a loop at *Sox6* with H3K27me3 signaling (Figure 4C). RT‐qPCR confirmed the aberrant expression of *Sox6* in the cell model and NTD tissues (*p*‐value < 0.05, *t*‐test; Figure S8C,D and Table S3, Supporting Information). To confirm whether chromatin loop changes were a direct reason for the change in the expression levels of *Sox6*, CRISPR/Cas9 gene editing was utilized to delete a differential loop near the DSB hotspots in the *Sox6* locus (Tables S3 and S5, Supporting Information). RT‐qPCR was performed using single‐cell clones from the target gRNAs. *Sox6* mRNA levels decreased compared to those in the control group (*p*‐value = 0.04, *t*‐test; Figure S8E, Supporting Information). Dysregulation of *Sox6* expression hinders the molecular separation of progenitor cells and differentiation of cortical interneurons. *Adamtsl5* and *Plk5*, which are crucial for the formation of extracellular microfibrils and development of neurons,^[^ ^29^ ^]^ showed increased transcription in the context of NTDs. These alterations may be related to the loss of chromatin loops labeled with the H3K27me3 signal (Figure 4D). By contrast, reduced expression of the repressor *Trim66* appeared to be associated with the loss of chromatin loops marked by the H3K27ac signal (Figure S8F, Supporting Information).

DSBs are the intrinsic components that maintain the 3D conformation of DNA during transcription. The organization of the genome impacts both the loci where DSBs form and the pairs of loci that join to form SVs.^[^ ^30^ ^]^ To evaluate this, we used Eagle C on Hi‐C data to analyze the new SVs induced by MTX treatment in the mESC, brains, and spines (Figure 4E). The results showed MTX treatment significantly increased chromosomal translocations in mESC (Figure 4F). Furthermore, significant variations in gene expression appear to be related to nearby structural changes. For example, the gene *Dpp6* has been reported to impact neuronal excitability and plasticity.^[^ ^21^, ^31^ ^]^ Folate deficiency–induced DSBs chromosomal inversions in mESC resulted in truncation of the *Dpp6* gene (Figure 4G). Furthermore, a correlation between SVs and changes in their expression has been observed in mouse spines. We observed significant chromosomal inversions associated with NTDs in the genomic regions of *Gm20707* and *Gm49083*, which might explain their increased expression (Figure 4H; Figure S8G, Supporting Information).

### 2.5 Dysregulation of Neural Tube Closure–Associated Genes with DSB‐Associated Chromatin Loops in Human NTDs

We assessed the degree of overlap among the eight distinct gene definitions to explore potential correlations between the dysregulation of neural tube closure–associated genes and DSB‐associated chromatin loops and their expression (Figure S9A, Supporting Information). Our analysis revealed that alterations in DSBs and chromatin loops have a direct and pronounced impact on gene expression. To verify them in human NTDs, 90 significantly differentiated genes among 560 neural tube closure–associated genes from infant brains with NTDs were selected and analyzed using NanoString (Table S7, Supporting Information), and five selective gene annotation methods were used to investigate their overlaps. Among the examined genes, 12 exhibited alterations in both DSBs and chromatin loops. Notably, these 12 genes demonstrated changes in expression in human NTD fetal brains and were previously linked to NTDs (**Figure** **5**A,B). To measure the effect of folate deficiency–induced DSBs on the regulation of NTD gene rewiring of loop interactions, we used CRISPR/Cas9 gene editing technology to delete the genomic regions with the highest DSB ratio by anchoring the representative differential loop on *Ift122*, which was one of the 12 genes identified above. Three sgRNA targets were designed. sgRNA1 and sgRNA2 targeted the distal promoter–enhancer loop of the *Ift122* locus with DSB hotspots, whereas sgRNA3 targeted the proximal promoter–enhancer loop of the *Ift122* locus without DSB hotspots (Figure 5C and Table S4, Supporting Information). First, the promoter–enhancer loop of the *Ift122* locus was confirmed in three biological replicates of mouse embryonic fibroblasts (Figure 5D). Sanger sequencing confirmed the homozygous deletion in a single‐cell–derived clone (Figure S9B and Table S6, Supporting Information). CRISPR/Cas9 on *Ift122* was confirmed using a 3C assay (Figure S9C,D and Table S1, Supporting Information). Following the targeting of sgRNA1 and sgRNA2, *Ift122* RNA expression was found to be significantly increased in single‐cell clone1 and 2 (*p*‐value = 0.01, *t*‐test; Figure 5E) (*p*‐value = 0.007, *t*‐test; Figure 5F), which was consistent with the results of the NanoString nCounter assay in NTD fetal samples (Figure 5B and Table S2, Supporting Information). The mRNA levels of two genes located outside the loop (*Alox5* and *Cand2*) showed no changes. Following the targeting of sgRNA3, *Ift122* RNA expression did not change in the single‐cell clone compared to that in the control group (data not shown). In addition, RT‐qPCR was performed using single‐cell clones treated with MTX. Again, *Ift122* was expressed at higher levels following MTX treatment than in single‐cell clones targeting sgRNA1 (*p*‐value = 0.04, *t*‐test; Figure S9E, Supporting Information) and sgRNA2 (*p*‐value = 0.006, *t*‐test; Figure S9F, Supporting Information). *Alox5* and *Cand2* mRNA expression did not change significantly after MTX treatment. This indicates that the integrity of DSB‐associated dysregulation of the chromatin loop targeting *Ift122* is crucial for its expression. *Axin2*, one of the 12 identified genes, was also verified to be dysregulated with a DSB‐associated chromatin loop in NTD mouse models (Figure S10A–C and Tables S3,S5, and S8, Supporting Information). The expression of *Axin2* was found to be regulated by chromatin loop changes near DSB hotspots at the *Axin2* locus targeted by sgRNA targets (Figure S10D,E and Table S8, Supporting Information). Taken together, these data suggest that folate deficiency–induced DSBs at loop anchors contribute to the misexpression of neural tube closure–associated genes.

Figure 5NTD genes dysregulation with DSB‐associated chromatin loop in human NTD. A) Upset plot exhibits that genes with DSB and chromatin loop changes are colocalized with gene expression changes or NTDs. B) Boxplots comparing normalized mRNA levels of 12 NTD candidate genes between healthy controls and NTD‐infant samples. Genes are displayed with human symbols and mouse orthologs. *n* represents the numbers of normal or NTD samples. Data represent mean ± SEM from *n* = 3 biological replicate. Statistical significance was determined by the Wilcoxon test. C) CRISPR/Cas9 gene editing was utilized to delete a differential loop in the *Ift122* locus nearby DSB hotspot. A combination of two different gRNAs cloned into vectors conveying puromycin resistance to allow for double selection after the lentiviral transduction of NIH‐3T3‐cells. Two single‐cell clones obtained from two different gRNA combinations carrying the homozygous deletion and the same single cell clones that underwent no CRISPR system treatment. RT‐qPCR assays were performed to verify the transcription. The best clone is shown, while the two remaining with MTX are included in Figure S9 (Supporting Information). *Ift122* gene inside the loop as well as two genes *Alox5*, *Cand2* located outside on either side of the loop were studied. D) Heatmaps showing the chromatin interaction at the *Ift122* locus in three biological replicates of mouse embryonic fibroblasts. E,F) RT‐qPCR data of gene expression for two different single cell clones carrying the homozygous deletion of the right DSB hotspots of a differential loop in the *Ift122, Alxo5, Cand2* locus vs control group. The control group is also single‐cell clones that underwent the same RISPR system treatment with unrelated region targeting. All expressed genes inside the loop as well as two genes located outside the loop were studied. Data represent mean ± SEM from *n* = 3 biological replicate. Statistical significance was determined by unpaired two‐tailed Student's *t*‐test. G) Conceptual model suggesting that DSB and higher‐order chromatin structures alterations contribute to MTX‐induced NTDs neurodevelopmental abnormalities.

**Figure:**
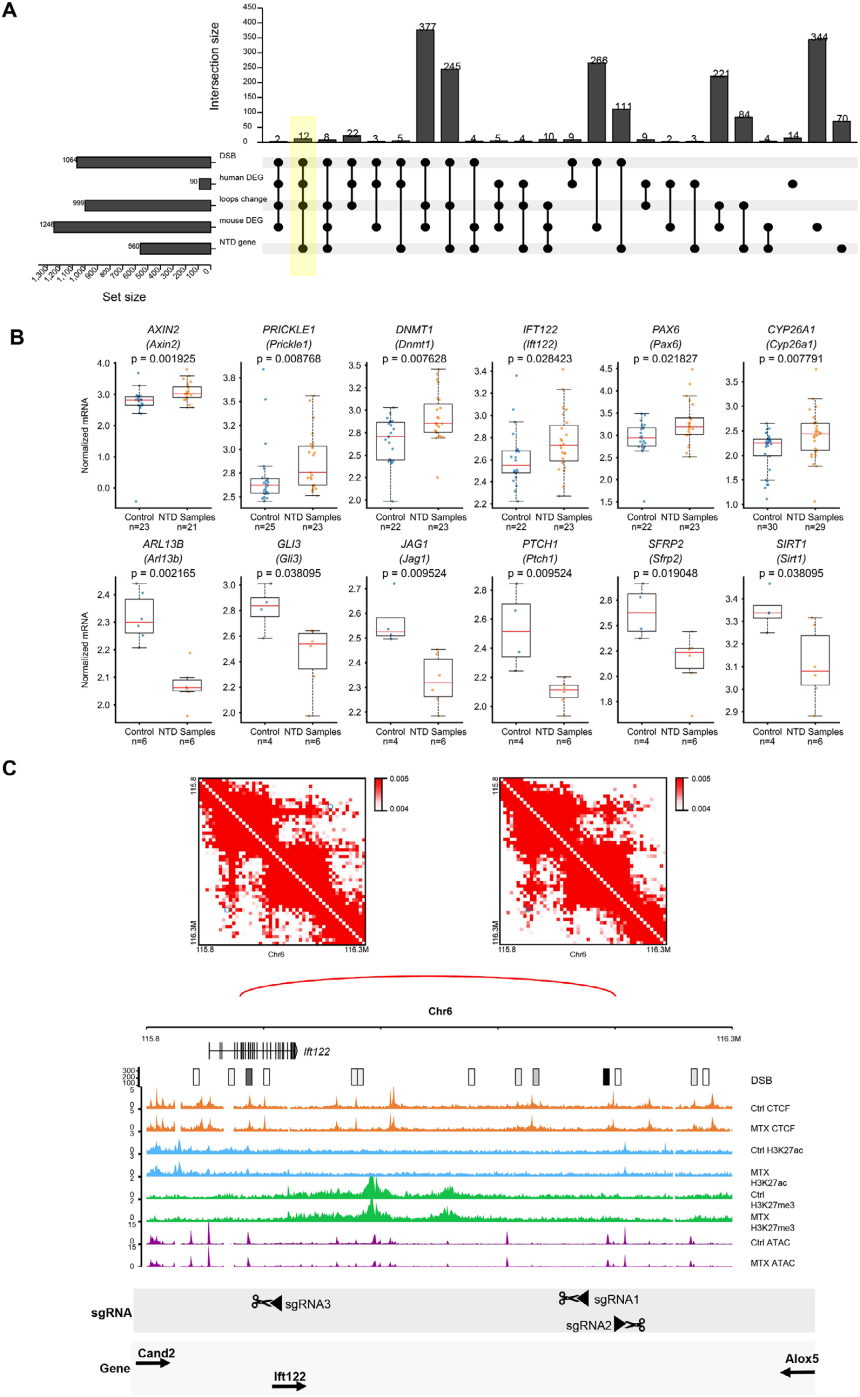

**Figure:**
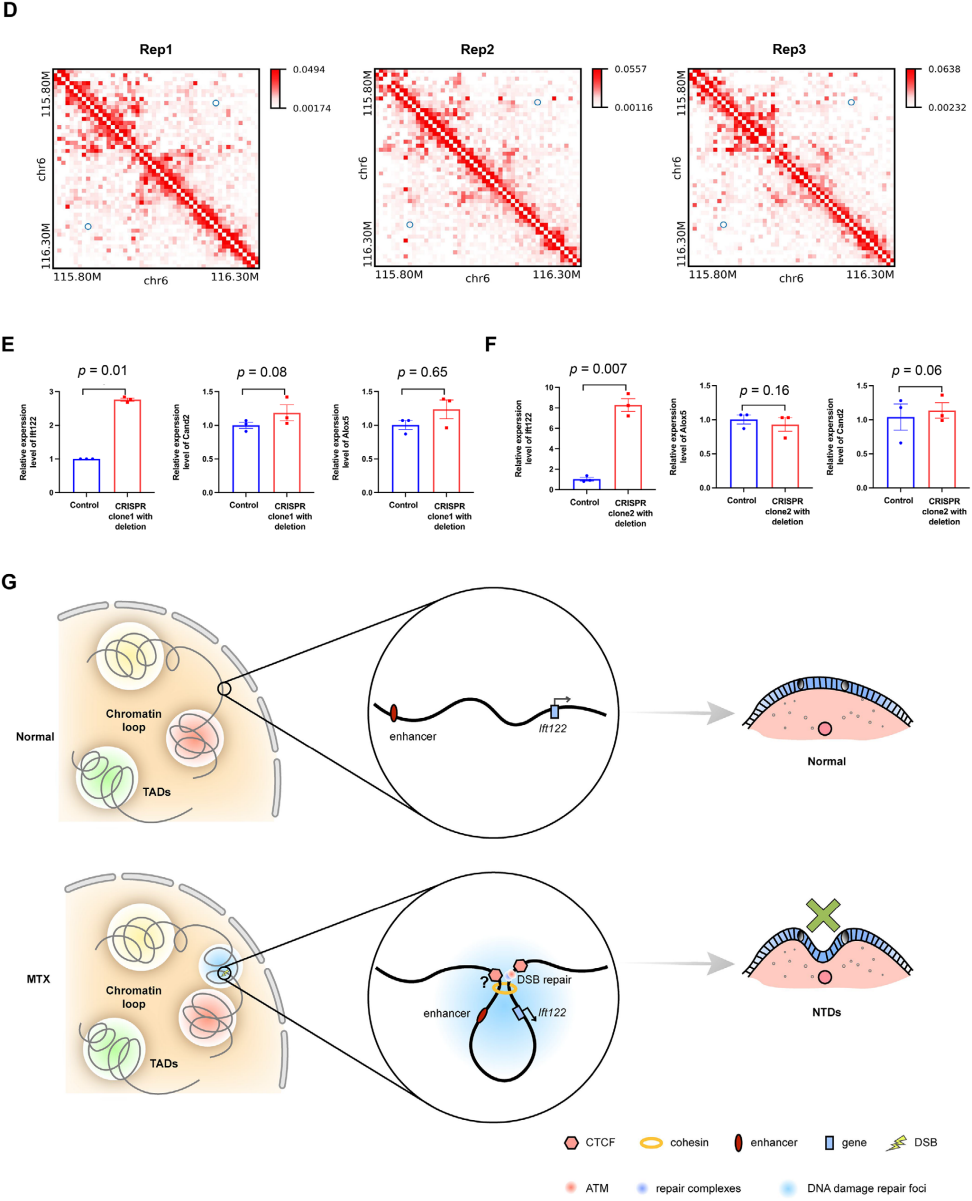

## 3 Discussion

Folate and other B vitamins play crucial roles in epigenetic regulation. Folate‐related epigenetic markers have been proposed as plausible mechanisms underlying the association between folate levels and various disease outcomes such as NTDs.^[^ ^22^, ^32^ ^]^ Animal models are pivotal for investigating NTDs and severe congenital malformations arising from failed tube closure during embryogenesis. Using animal models such as mice, chicks, *Xenopus*, and zebrafish, researchers have successfully constructed many NTD models to study the underlying mechanisms of failed neural tube closure.^[^ ^33^ ^]^ Genetically tractable mice are widely used via chemical induction (e.g., retinoic acid exposure, folic acid antagonists, valproic acid, and hyperglycemia) or gene knockout (e.g., *Pax3* and *Sox2* mutations) to mimic human NTDs, such as spina bifida.^[^ ^34^ ^]^ DHFR is the key enzyme involved in folate metabolism. It catalyzes the reduction of folic acid and dihydrofolate, the only pathways that activate the oxidized form of folate. In this study, we injected MTX, a specific inhibitor of DHFR, into pregnant mice.

Our previous study revealed that the disruption of one‐carbon metabolism results in increased genome instability and chromosomal breakage with folate deficiency. However, a gap remains in understanding the relationship between chromosomal breakage and NTD phenotype. Several methods have been proposed in the past for capturing chromosomal breaks. For example, pyridoxamine‐induced DNA damage was detected with ChIP‐seq using the DNA damage marker gH2AX.^[^ ^22b^ ^]^ Single‐nucleotide resolution DSB identification can also be verified using DSB Capture.^[^ ^8b^ ^]^ An approach based on Pulsed‐field gel electrophoresis (PFGE) was used to detect DSB fragments containing DNA replication sites.^[^ ^22b^ ^]^ All these methods provide powerful ways to identify DSBs under various conditions; however, the quantitative evaluation of DSBs and the biological significance of their levels have rarely been mentioned and remain of interest. However, questions remain regarding whether changes in genomic, epigenetic, and gene expression interactions in the presence of folate deficiency lead to gene dysregulation, and thus, abnormal neural tube development. Most published genetic studies of NTDs have focused on neural tube closure–associated genes derived from folate metabolic pathways or identified in mouse NTD models. Moreover, no published large‐scale GWAS or epigenetic studies have focused on neural tube closure–associated genes. Thus, further high‐throughput sequencing using approaches such as Hi‐C is required to extend the discovery of the dynamic transcriptional characteristics of neural tube closure–associated genes.

Most TADs are invariant among different cell types and species, suggesting that they act as fundamental functional units of the genome.^[^ ^15b^ ^]^ We analyzed the expression of 560 neural tube closure–associated gene expression in the nervous tissues of patients with NTDs. Some of these genes have been confirmed to be related to neural tube closure. ≈83% of the DEGs were caused by changes in the DSB gene structure and 3D chromatin organization. Particularly, almost half of these genes were affected by both DSB changes and 3D chromatin structures, suggesting that DSB formation contributes to the disruption of 3D chromatin structures involved in neurodevelopmental gene dysregulation. In addition, we characterized the remaining half of the neural tube closure–associated genes that were influenced by modifications in the 3D chromatin structure. Our CRISPRi and anchor deletion results also indicate that the low folate‐induced dynamics of such domains change the precision of gene regulation by facilitating enhancer–promoter contacts in distal gene noncoding regions when compared with knockdown of proximal gene noncoding regions. All these factors are consistent with the more complex role of the 3D chromatin structure in transcriptional regulation, which may be a prerequisite for the transcriptional changes involved in NTDs. Neurodevelopmental abnormalities are regulated by a combination of mechanisms inferred as 3D genome.

DSBs occur during physiological transcription, DNA replication, and antigen receptor diversification. Folate is required to transfer one carbon unit during the de novo synthesis of nucleotides. Although folate deficiency–induced DSBs tend to accumulate in promoters and active enhancers, similar to endogenous DSBs, some differences exist between them. For endogenous DSBs, transcription did not have a causal effect on DSB formation, as revealed by the logistic models and causal association tests. In our previous study, we assessed the quality and quantity of DSB enriched in both normal and MTX‐treated mESCs using next‐generation sequencing.^[^ ^14^ ^]^ We also calculated global DSB levels by dividing the DSB enrichment in MTX‐treated mESCs by the DSB enrichment in normal mESCs. Measuring these DSB levels eliminates the bias from endogenous DSBs in mESCs, thereby reflecting genomic instability following MTX treatment. Importantly, DSB levels have a biological significance in gene expression and regulation. DSB levels were negatively correlated with the distance between the TSSs and DSBs. The abundance of regulatory elements was positively correlated with DSB ratios. The DSB levels were found to be correlated with TADs in chromatin, a pronounced surge in the DSB ratio, and a marked augmentation of gene transcription throughout the TAD spectrum. Additionally, SVs linked to DSB were correlated with neural gene expression in the brains of patients with NTDs. The DSB levels quantitatively characterized in our study are meaningful and provide new insights into the functions of DSBs in various diseases. However, additional experiments are necessary to determine how the DSB‐coordinated 3D chromatin structure that disrupts neurodevelopmental gene expression contributes to NTD phenotypes. Furthermore, it is worth noting that MTX‐treated fetuses without NTD phenotypes were not included in our study. Without examining these unaffected fetuses, the status of the DSBs in neural tissues remains undetermined. This gap hinders definitive conclusions regarding whether the observed DSB alterations are specifically pathogenic (i.e., causal of NTDs) or merely generalized responses to folate disruption, which may occur even in the absence of overt malformations. Future studies should include controls to clarify the specificity of DSB dysregulation in driving NTD pathogenesis.

In this study, we constructed a genome‐wide genetic landscape of folate deficiency using multidimensional datasets. Our results suggest that DSBs can alter the interaction between regulatory elements and genes, and further affect transcriptional activity. Taken together, our research reveals that folate deficiency causes a one‐carbon group disorder, leading to impaired DNA synthesis and a pathological increase in DSBs. Therefore, the abnormal expression of neural tube closure–associated genes, is considered as one of the important mechanisms underlying the occurrence of NTDs. Our findings underscore the significance of understanding genes, their structural intricacies, and the 3D chromatin organization in relation to DSBs. Excessive DSB‐associated 3D genome organization disruption within NTDs with folate deficiency contributes to the dysregulation of neural tube closure–associated genes. These models have the potential to aid the development of therapeutic interventions by manipulating the 3D chromatin structure.

## 4 Experimental Section

### Cell Cultures and MTX Treatment

mESCs Sv/129 were provided by Xuanwu Hospital (Beijing, China). The mESCs were maintained in mitotically inactivated primary mouse embryonic fibroblasts before cultivation under feeder‐free conditions. Mouse embryonic fibroblasts, NIH‐3T3 cells, were purchased from the Stem Cell Bank of the Chinese Academy of Sciences (Shanghai, China). The cells were maintained at 37 °C in a humidified atmosphere with 5% CO_2_ and passaged every 2–3 days. Split ratios ranging from 1:4 to 1:7 were used. MTX (Sigma) was applied to the mESCs at a final concentration of 0.12 µm, 24 h after seeding, as MTX treatment. NU7026 (Calbiochem) was used at a final concentration of 5 mm.

### Animals

CD‐1 mice (7–8 weeks old) were provided by Beijing Vital River Animal Technology Co., Ltd., China (no. 110011200105514958, 25 g). Female CD‐1 mice were fed a low‐folate diet for more than 8 weeks. Sexually mature individuals were mated overnight. The vaginal plug was detected at 8:00 a.m. the following morning and designated as Embryonic day 0.5 day (E0.5d). NTD mouse models were induced by intraperitoneal injection of 1.5 mg kg^−1^ MTX (Sigma–Aldrich), and control mouse models were injected with phosphate‐buffered saline (PBS) instead of E7.5. On E9.5, pregnant mice were euthanized by cervical dislocation, and fetal phenotypes were observed under a microscope. Incidence of NTD are presented in Table S9 (Supporting Information). All the samples showing clinical manifestations of NTDs, including anencephaly and spinal bifida, were collected. The brains and spinal cords were collected for the experiments. Littermates without NTD phenotypes following MTX treatment were also excluded. No specific randomization method was used in this study. Experiments were carried out following the National Institutes of Health's Guide for the Care and Use of Laboratory Animals. This study was approved by the Animal Ethics Committee of the Capital Institute of Pediatrics (approval number B374).

### Determination of Folate Concentrations

The folate concentrations in cells and tissues were determined using competitive receptor‐binding immunoassay kit (Cat.#A14208; Chemiluminescent Immune enzyme Assay, Beckman Coulter, USA) and Chemiluminescent Immune enzyme assay access II (Beckman Coulter, Fullerton, Germany). Briefly, 10^6^ cells or 15 mg of brain tissue were collected in 1 mL of Tris buffer solution, sonicated for nine cycles, and then centrifuged at 10 000 rpm for 3 min at 4 °C before the supernatant was tested.

### 3 C Assay

According to a previously published protocol,^[^ ^35^ ^]^ 2 × 10^6^ cells were isolated and cross‐linked with 1% formaldehyde for 10 min at room temperature. Glycine solution (2.5 M) was added at a final concentration of 0.2 M. Fixed cells were lysed in ice‐cold Hi‐C lysis buffer (10 mm Tris‐HCl [pH 7.5], 10 mm NaCl, and 0.2% NP‐40) for 15 min. Then the cells were centrifuged at 2500 × *g* for 5 min at 4 °C to remove the lysis buffer and supernatant. ≈43 µL of 10% Triton X‐100 was added and the mixture was incubated at 37 °C for 15 min. Next, 12 µL of 10×NEBuffer 2 and 400 units of MboI enzyme (R0147; NEB) were introduced for overnight digestion at 37 °C on a rocking platform. On the subsequent day, the MboI enzyme was inactivated by heating at 65 °C for 30 min. The DNA was then religated using T4 DNA ligase, incubated at 24 °C for 4 h with intermittent manual agitation. Finally, 30 µL of proteinase K was added and incubated overnight at 65 °C, after which DNA was extracted using a phenol/chloroform mixture. Finally, PCR and Sanger sequencing were used to validate long‐range chromatin interactions. The specific PCR primers are detailed in Table S1 (Supporting Information).

### Western Blotting

Total protein was extracted from the cells using RIPA lysis buffer (Beyotime, China) containing 1 mmol L^−1^ phenylmethylsulfonyl fluoride (Beyotime, China). A cocktail of phosphorylase and protease inhibitors was added to prevent protein degradation. A BCA Protein Assay Kit (Beyotime, China) was used to determine protein concentration. The boiled protein samples were separated on a 10% sodium dodecyl sulfate‐polyacrylamide gel and transferred onto a polyvinylidene fluoride (Beyotime, China) membrane, which was wetted in 100% methanol for 15 s. Thereafter, membranes were blocked in 5% BSA blocking buffer at 37 °C for 1 h and incubated overnight at 4 °C with the primary antibodies against GAPDH (1:10 000), anti‐ASCL1 (1:1000), and anti‐ZEB1 (1:1000). The secondary antibodies were horseradish peroxidase (HRP)‐conjugated goat anti‐rabbit (1:5000) and HRP‐conjugated goat anti‐mouse (1:5000), incubated for 1 h at room temperature. Finally, signals were detected using an enhanced chemiluminescence detection reagent (Thermo Fisher Scientific). Immune complexes were detected using enhanced chemiluminescence kit (Wbkls0100, Millipore), quantitative optical density analysis was performed with Image‐Lab 6.1 software.

### Immunohistochemical Analysis

Nervous tissue samples from animals were fixed in formalin and embedded in paraffin. The tissue blocks were cut into gauze sections and fixed on glass slides. Sections were deparaffinized and antigens were exposed using a wet heat epitope retrieval method. Sections were incubated overnight at 4 °C with the primary anti‐ASCL1 antibody (1:100), anti‐ZEB1 antibody (1:2000), and anti‐Axin2 antibody (1:200). After washing three times with PBS, secondary antibodies (HRP‐conjugated goat anti‐mouse antibody (1:200)) were added and incubated for 1 h at room temperature. The sections were visualized by diaminobenzidine staining for 10 min at room temperature and counterstaining with hematoxylin. The sections were dehydrated and mounted with neutral balsam. The number of positive cells was counted under a microscope by a single investigator. According to the integrated optical density (IOD), expressions were collected using Image‐Pro Plus 9.0 under the microscope. The sections of each sample were selected for IOD analysis by a single investigator to minimize bias.

### Immunofluorescence Microscopy

Mice were transcranially perfused with ice‐cold PBS and fixed with ice‐cold 4% paraformaldehyde in PBS. Dissected mouse brains or spines tissue were drop‐fixed overnight in 4% paraformaldehyde at 4 °C. Brain tissue or spines were sectioned using vibrating microtome (Leica BioSystems, Wetzlar, Germany) to generate 40 mm coronal slices. Slices were blocked for 2 h at room temperature in blocking buffer (10% NGS, 0.3% Triton X‐100, PBS), then incubated with primary antibody overnight at 4 °C. After washing two to three times with PBS, secondary antibodies were added and incubated for 30 min. DAPI was used as the counterstain. The slides were imaged using Leica DMI6000B microscope (Leica). At least two coronal slices were used for image quantification for each mouse.

### Chromatin Immunoprecipitation Assay

Following the manufacturer's protocol, a SimpleChIP™ Enzymatic Chromatin IP Kit (CST) was used for the ChIP assays. Briefly, formaldehyde‐cross‐linked chromatin was obtained from ≈2 × 10^7^ mESCs. Cross‐linked chromatin was immunoprecipitated using antibodies against H3K4me1, H3K4me3, H3K27me3, H3K27ac, H3K9me3, gamma‐phosphorylated H2AX, and CTCF. Nonspecific mouse IgG and H3 were used as negative and positive controls, respectively.

### RNA Extraction, Reverse Transcription, and Real‐Time RT‐PCR Analysis

Total RNA was isolated using TRIzol reagent (Invitrogen, Carlsbad, CA, USA), following the manufacturer's recommendations. Reverse transcription was performed using TransScript First‐Strand cDNA Synthesis SuperMix (TransGen Biotech, Beijing, China). UltraSYBR Mixture (CWBiotech, Beijing, China) was chosen for real‐time PCR, and analysis to quantify the relative mRNA levels in the different cell groups was then performed using QuantStudio™ 7 Flex Real‐Time PCR system (Applied Biosystems instruments, CA, USA). The reaction composition was as follows: forward and reverse primers (0.5 and 10 µmol, respectively), UltraSYBR Mixture (12.5 µL; containing TransStart Taq, SYBR Green I, dNTPs, and PCR enhancer), cDNA (1 µL), and ddH_2_O (10.5 µL). After the initial denaturation step at 95 °C for 3 min, 45 amplification cycles were performed as follows: denaturation at 95 °C for 15 s, annealing at 58 °C for 20 s, and extension at 72 °C for 30 s, followed by a final step of 10 min at 72 °C. Gene expression was normalized to that of GAPDH. The relative levels of the mRNA transcripts were calculated using the classical ΔΔCt method. The primers used in this experiment are listed in Table S3 (Supporting Information).

### RNA Sequencing

The total RNA was extracted as previously described. Proteins were assessed using denaturing agarose gel electrophoresis and quantified using NanoDrop spectrophotometer (NanoDrop, USA). Library preparation and RNA sequencing were then performed. Briefly, the total RNA was treated with Epicenter Ribo‐Zero kit to remove all rRNAs. The remaining RNAs were processed using TruSeq RNA Sample Prep Kit following the Illumina protocol. RT‐PCR was performed using the Phusion High‐Fidelity DNA polymerase, Index (X) Primer, and Universal PCR primers. Finally, the products were purified using AM Pure XP system, and library quality was evaluated using the Agilent Bioanalyzer 2100 system. The RNA library was sequenced on an Illumina HiSeq 4000 platform, and 150 bp paired‐end reads were generated.

### Assay for Transposase‐Accessible Chromatin (ATAC) Sequencing

Fresh cells and tissues were collected after centrifugation at 500 × *g* and washed twice with cold PBS. Chromatin‐enriched fractions were extracted with cold lysis buffer and the pellets were resuspended in transposition reaction buffer containing Tn5 transposases (Nextera DNA Sample Prep Kit; Illumina Nextera). Transposition reactions were incubated at 37 °C for 30 min, followed by DNA purification using DNA Clean‐up and Concentration kit (Zymo, USA). The libraries were initially PCR‐amplified using NEB Next High‐Fidelity 2×PCR Master Mix (NEB, UK). The number of cycles was maintained between 12 and 15. The final amplified library was purified using Zymo DNA Clean‐up and Concentration kit. The DNA was evaluated using TBE gel electrophoresis and quantified using Agilent 2100 Bioanalyzer Nanodrop. The libraries were multiplexed and paired‐end 150 bp sequencing was performed using Illumina HiSeq 4000.

### ChIP Sequencing

DNA libraries of H3K4me1, H3K27ac, H3K4me3, H3K27me3, H3K9me3, phosphor S139 gamma H2A.X, and CTCF were prepared for HiSeq 4000 sequencing as described previously.^[^ ^22d^ ^]^

### DSB Enrichment Workflow

The DSB workflow had been summarized previously.^[^ ^14^ ^]^ Briefly, mESCs as single‐cell suspensions were centrifuged and washed. The cells were then lysed in lysis buffer containing 10 mm Tris–Cl (pH 7.4), 10 mm NaCl, 3 mm MgCl_2_, and 0.1% IGEPAL CA‐630. The suspension was centrifuged immediately at 500 × *g* for 10 min at 4 °C. The nuclei were carefully embedded in 0.8% LMP agarose melted in sterile 50 mm EDTA (pH 8.0), and washed them with fresh LIDS buffer containing 10 mm Tris–Cl (pH 8.0), 100 mm EDTA, and 1% lauryl sulfate lithium salt overnight at 37 °C. After washing overnight, the LMP agarose was washed twice by 10 mm Tris–Cl and 0.25 mm EDTA (pH 8.0), with shaking at 60 rpm at room temperature. Blunt ending of the DSB ends was performed. DNA was carefully extracted using phenol:chloroform and precipitated from LMP agarose using alcohol. The DNA was ligated to 20 pmol of biotinylated linker 1 in a 100 µL reaction volume containing 400 U of T4 DNA ligase by incubation at 16 °C overnight. After gel purification, the DNA ligated with linker 1 was treated with *MmeI* (NEB) according to the manufacturer's instructions. For PCR amplification, the suspension was prepared using a Dynal bead suspension, Phusion polymerase (NEB), sequencing primers, and dNTP. The PCR cycles were as follows: initial denaturation at 98 °C for 2 min, followed by 24 cycles of 98 °C for 10 s, 65 °C for 30 s, 72 °C for 30 s, with a final extension at 72 °C for 5 min. DSB enrichment products were obtained by electrophoresis and gel purification. Clustering was performed using TruSeq PE Cluster Kit v3‐cBot‐HS, and sequencing was carried out on the Illumina HiSeq 2000 platform using TruSeq SBS Kit v3‐HS chemistry.

### Sequencing Data Processing—RNA‐Seq Analysis

RNA‐seq experiments were conducted in triplicate. Individual runs showed a high degree of correlation with the promoters. Sequencing reads were trimmed using Trim Galore (version 0.6.0), a Perl wrapper based on Cutadapt and FastQC. Reads with a quality >20 were aligned to the mouse reference genome (mm10) using TopHat2 (version 2.1.1).^[^ ^36^ ^]^ Gene expression levels were calculated using Cufflinks software (version 2.2.1).^[^ ^37^ ^]^ The relative abundance of transcripts was measured as fragments per kilobase of exons per million mapped reads (FPKM). Differential expression between different groups was tested using the Cuffdiff module, and genes with a q‐value < 0.05 were selected as being differentially expressed. The GTF annotation file for UCSC mm10 (GRCm38.81) was used for the gene annotation.

### Sequencing Data Processing—ChIP‐Seq Analysis

ChIP‐seq reads were trimmed using Trim Galore (version 0.6.0). Reads with quality >20 were aligned to the mouse reference genome (mm10) using STAR (version 2.7.0).^[^ ^38^ ^]^ Reads with low mapping quality (<20) were discarded using SAMtools. Any duplicate reads were marked and removed using PICARD software. ChIP‐seq peaks were called using MACS (version 1.4.2) with the parameters –*p* ×10^−6^ –nolambda –nomodel.

### Sequencing Data Processing—CUT&Tag Analysis

The CUT&Tag experiments were performed as previously described (TD903, Vazyme) to generate DNA libraries derived from mouse tissues.^[^ ^35^, ^39^ ^]^ CUT&Tag sequencing reads were first trimmed using fastp and then aligned to mouse genome reference mm10 with Bowtie2 (v.2.3.5.1) with “–end‐to‐end –very‐sensitive –no‐unal –no‐mixed –no‐discordant –phred33 ‐I 10 ‐X 700” options. Any PCR duplicates were removed using Picard MarkDuplicates tool with “VALIDATION_STRINGENCY = LENIENT” option. MACS2 (v.2.2.4) was used for peak calling for histone marks and TFs with “‐p 1e‐2 –nomodel –shift 0 –keep‐dup all ‐B –SPMR” options.

### Sequencing Data Processing—ATAC‐Seq Analysis

ATAC‐seq experiments were conducted in triplicate. Individual runs showed a high degree of correlation with the promoters. The sequencing reads were trimmed using Trim Galore (version 0.6.0). Reads with quality >20 were aligned to the mouse reference genome (mm10) using STAR (version 2.7.0).^[^ ^38^ ^]^ Reads with low mapping quality (<20) were discarded using SAMtools. Any duplicate reads were marked and removed using PICARD software. ATAC‐seq peaks were called using MACS (version 1.4.2) with the parameters –*p* ×10^−6^ –nolambda –nomodel.

### Sequencing Data Processing—Hi‐C Data Analysis

Paired‐end reads obtained by sequencing were mapped to mm10 using HiC‐Pro (v3.1.0).^[^ ^40^ ^]^ Default settings were used to remove duplicate reads, identify valid interactions, and generate contact maps. All ValidPairs files were used to merge technical duplicates and downsample reads for consistent sequencing depth. The “*. allvalidpairs*” files were converted into “*.hic*” files using Juicer tools (v1.22.01).^[^ ^41^ ^]^ The “.*hic*” files were converted to “.*Mcool*” files using hic2cool (v0.8.3).^[^ ^42^ ^]^ Hi‐C compartments were identified at 100‐kb resolution using dcHiC.^[^ ^43^ ^]^ Hierarchical TAD was defined at 50‐kb resolution using TAD (v1.4)^[^ ^44^ ^]^ with*.hic* as input. Loops were identified at a 10‐kb resolution using Peakachu.^[^ ^45^ ^]^ The differential loops were calculated according to the probability of loop output by Peakachu (loops with a probability greater than 0.95 in this sample and less than 0.8 in the control group, were defined as the differential loops of this sample). APA was performed using HiCPeaks software (https://github.com/XiaoTaoWang/HiCPeaks) for visualization. Joint epigenomic analyses were performed using pyGenomeTracks.^[^ ^46^ ^]^

### Sequencing Data Processing—DSB Data Analysis

Data were first converted to the FASTQ format using Illumina Casava 1.8 software. Quality evaluation was performed using FastQC 0.10.1 software. Joint sequences (FASTX) were removed from the sequencing data, and the length was then filtered. Sequences containing the correct enzyme digestion sites were retained based on the conditions encoded by the in‐house Perl script. The sequence was mapped onto the mouse genome sequence and alignment (mm10) to obtain valid sequences that aligned to the mouse genome. To analyze the data in an unbiased fashion, the data were median‐smoothed from 5 to 1000 K (steps of 5 K) as a bin to obtain the valid read counts of each sample. The DSB resolution was set at 5 kb for subsequent analyses. Statistics on fold changes or *p* values were calculated using HYPGEOMDIST in R. ANNOVAR and DAVID tools were used for gene annotation in the DSB enrichment regions.

### Sequencing Data Processing—Busy Anchor Analysis

The busy anchor was defined: For Hi‐C data, based on the calculated loops (two anchors define a loop), the frequency of anchor occurrence in all loops was counted, and an anchor whose frequency was greater than or equal to two was defined as a busy anchor. The busy anchor was then further analyzed using a set of Hi‐C data (including control and treatment groups) combined with previously defined differential loops. All loop anchors in the Hi‐C data were divided into busy and normal anchors, and the number of anchors overlapping the differential loops was counted. Based on the aforementioned statistics, the *scipy.stats.chi2_contingency* test was applied to the variable independence in the contingency table between busy and normal anchors to verify the correlation between busy anchors and loop changes.

### Sequencing Data Processing—SV Analysis

First, a cooler was used for preprocessing “*.mcool*” file as input to calculate the SV.^[^ ^47^ ^]^ The iterative correction and eigenvector decomposition (ICE) method was used for Hi‐C normalization with the “cooler balance” option. Second, SV was calculated using Eagle C,^[^ ^21a^ ^]^ and SV changes were obtained by comparing the control and treatment groups. PyCircos (https://github.com/ponnhide/pyCircos) was used to visualize chromosomal translocations. The results of the joint analysis of the SVs and epigenomics were visualized using the visualization module in NeoLoopFinder.^[^ ^48^ ^]^

### Correlation Between Gene Expression and DSB Enrichment

The DSB enrichment was calculated based on previous reports.^[^ ^14^ ^]^ If the TSS of a given gene was located within a DSB enrichment region, the gene was considered to have enriched DSBs. Genes with expression levels (FPKM) >1 were sorted in descending order of DSB enrichment and splitted into 100 equally sized bins. Average expression levels and DSB enrichment were calculated for each gene bin. The correlation between gene expression and DSB enrichment was calculated using Pearson's correlation coefficient.

### Calculation of Epigenetic Signal Enrichment Surrounding Gene TSSs

As shown in Figure 3D, genomic regions ±5 kb around the TSSs were splitted into 100 equally sized bins, and the average ATAC‐seq and ChIP‐seq density in each bin was calculated in units of reads per kilo million per base pair (RPKM).

### Analysis of Chromatin State

Chromatin states were defined from the ChIP‐seq data, including H3K4me1, H3K4me3, H3K27me3, and H3k27ac, using ChromHMM with a ten‐state model because a larger number of states did not appear to identify additional states. The resulting state names were then assigned based on patterns previously described in the NIH Roadmap and ENCODE projects: Active promoter (TssA; H3K4me3 and H3K4me1), Flanking promoter (TssFlnk; H3K4me3, H3K4me1, and H3K27ac), Weak/Poised Enhancer (EnhWk; H3K4me1), Active Enhancer 1 (EnhA1; H3K27ac and H3K4me1), Active Enhancer 2 (EnhA2; H3K27ac), and two Quiescent states with a low signal, which were merged (Quies/low; low signal for all assays).

### Participants

All samples were obtained from the Lvliang area of Shanxi Province, northern China. Informed consent for inclusion in the study was obtained from patients or their families. The enrolled pregnant women were diagnosed by trained local clinicians using ultrasonography and registered. The surgical procedures were performed as previously described.^[^ ^49^ ^]^ The study protocol was approved by the Ethics Board of the Capital Institute of Pediatrics. Information on the clinical samples is presented in Table S7 (Supporting Information).

### CRISPR/Cas9‐Mediated Loop Anchor Deletions

Specific gRNAs targeting the regions upstream and downstream of the selected distal enhancer loop anchor sites were designed. A combination of two gRNAs was used to generate deletions. Sense and antisense oligonucleotides containing the 25 bp gRNA sequence and the required overhangs matching the plentiCRISPR v2‐GFP and plentiCRISPR v2‐puro vectors were purchased and annealed. The plentiCRISPR v2‐GFP vector, plentiCRISPR v2‐puro vector, and T4 DNA ligase were used to clone the annealed gRNAs into plasmids containing expression cassettes for hSpCas9 and chimeric guide RNA. The vector used was LentiCRISPRv2, which confers resistance to puromycin. Thus, a combination of the two gRNAs cloned into the pMD2.G and pSPAX2 plasmids allowed for double selection after transduction (Table S8, Supporting Information). Correct insertion of gRNAs into the plasmid was verified using Sanger sequencing. Cell‐free lentiviral supernatants were produced by zKirus‐based transient co‐transfection of Lenti‐X 293T cells. Briefly, the pLentiGuide‐PurogRNA vector, lentiviral gag/pol packaging plasmid psPAX, and envelope plasmid encoding the vesicular stomatitis virus glycoprotein (VSV‐G) were transfected at a molar ratio of 4:1:1 using standard lipo3000 transfection. After 48 and 72 h of transfection, two consecutive viral supernatants were harvested, cleared through 0.45‐m pore‐size PVDF membrane filter, combined and then stored at −80 °C. Transduction with gRNA‐expressing viruses was performed by transfecting NIH‐3T3 cells with two consecutive spinoculations. Briefly, 0.2 × 10^6^ cells were pelleted and resuspended in 1 mL of lentiviral particles. Polybrene (8 µg mL^−1^) was added and the cells were centrifuged at 2100 × *g* for 120 min at 32 °C. The supernatant was discarded, the cells resuspended in 1 mL of full growth medium and incubated for 48–72 h. Cells were selected with 2 µg mL^−1^ puromycin for 10 days and then recovered in puromycin‐free medium for an additional week. Positive monoclonal cells were sorted for subsequent experiments using flow cytometry based on GFP expression. All the selected positive cells were cultured for 2–3 weeks to obtain stable cell lines with loop deletions. Successful deletions were confirmed using agarose gel electrophoresis of the PCR amplicons, with genomic DNA as a template (Table S6, Supporting Information).

### NanoString nCounter Assay

A NanoString nCounter was used to detect the expression of transcripts in fetal brain tissues. Total RNA was extracted according to the manufacturer's instructions (Qiagen), and gene‐specific probes were designed according to the manufacturer's instructions (NanoString Technologies). Hybridization was performed according to the nCounter Element 48‐plex Assay Manual (NanoString Technologies). Gene expression data were filtered using quality control (QC) criteria following the manufacturer's recommendations. The raw counts of QC‐passed samples were normalized to five reference genes used as internal controls (*ACTB*, *TUBB*, *CLTC*, *TBP*, and *POLR1B*). All QC and normalization procedures were performed using nSolver Analysis Software v2.0, and all data were log2‐transformed before further analysis. Student's *t*‐test were used to compare normalized expression values between normal tissues and NTDs.

### Antibodies

The following antibodies were used: rabbit polyclonal antibodies against H3K4me1 (ab8895; Abcam), H3K27ac (ab4729; Abcam), and H3K4me3 (39159; Active Motif); mouse monoclonal antibody against H3K27me3 (ab6002; Abcam); phosphor‐S139 gamma H2A.X antibody (05‐636; Millipore); rabbit monoclonal antibody against H3K9me3 (13969; Cell Signaling Technology); RAD21 with anti‐RAD21 antibody (ab217678; Abcam); anti‐H2A.X‐Phosphorylated (Ser139) antibody (ab22551; Abcam); anti‐GAPDH (ABL1020; Abbkine); anti‐ASCL1 antibody (ab240385; Abcam); anti‐ZEB1 antibody (ab303480; Abcam); anti‐Axin2 antibody (ab109307; Abcam); and HRP‐conjugated secondary antibodies such as goat anti‐rabbit (ZB2301; Zsbio), goat anti‐mouse (ZB2305; Zsbio), and goat anti‐mouse (ab970230; Abcam).

### Statistical Analyses

Statistical analyses were performed using a two‐tailed Student's *t*‐test for comparisons between two groups or ANOVA followed by Tukey's post hoc test for comparisons among more than two groups, after verifying the normality of the data. Statistically significant differences in all figures are indicated as *: *p* < 0.05; **: *p* < 0.01; ***: *p* < 0.001; and ****: *p* < 0.0001. All data are presented as the means ± SEMs. Statistical analyses were performed using SPSS 22.0 software.

### Ethics Approval and Consent to Participate

The study protocol was approved by the Ethics Board of the Capital Institute of Pediatrics, Beijing, China (approval number: [2015001]). Informed consent was obtained from the donors and their guardians. All procedures involving animal handling were performed in accordance with institutional guidelines approved by the Animal Ethics Committee of the Capital Institute of Pediatrics, Beijing, China (approval number B374).

### Availability of Data and Materials

The RNA‐seq, ATAC‐seq, ChIP‐seq, CUT&Tag data of mESCs, and animal models that support the findings of this study have been deposited in the National Center for Biotechnology Information Gene Expression Omnibus (GEO; https://www.ncbi.nlm.nih.gov/geo/) under the accession numbers GSE149627, GSE299189, and GSE299190. All Hi‐C data have been deposited in the Genome Sequence Archive of the National Genomics Data Center, China National Center for Bioinformation/Beijing Institute of Genomics, Chinese Academy of Sciences (GSA; CRA011730), which is publicly available at https://ngdc.cncb.ac.cn/gsa. The Hi‐C data of mouse embryonic fibroblasts were obtained from the GEO with the accession number GSE198989.^[^ ^50^ ^]^ All the other relevant data are available from the corresponding author upon request.

## Conflict of Interest

The authors declare no conflict of interest.

## Author Contributions

T.Z., L.L., and J.L. contributed equally to the paper as first authors. T.Z., Q.X., H.B.C., and H.L. conceptualized the study. B.X.M., Z.J.Z., F.W., Y.H.B., and D.G. collected the mouse and human samples. Q.X. and J.T.L. performed QC of ChIP‐seq, ATAC‐seq, Hi‐C, and RNA‐seq data sets. L.L. carried out all bioinformatics analyses of data sets as well as their integration and comparison with public data. H.L. and H.B.C. interpreted the results. Q.X. wrote the manuscript draft, together with L.L. and J.T.L. All authors reviewed and approved the final version of this manuscript.

## Supporting information



Supplemental Figures 1–10

Supplemental Table 1

Supplemental Table 2

Supplemental Table 3

Supplemental Table 4

Supplemental Table 5

Supplemental Table 6

Supplemental Table 7

Supplemental Table 8

Supplemental Table 9

## Data Availability

The data that support the findings of this study are openly available in GEO at https://doi.org/[doi], reference number 149627，299189，299190 and GSA at https://ngdc.cncb.ac.cn/gsa, reference number 011730.
