# Supplementary material for: Excessive DNA Double‐Strand Breaks–Associated 3D Genome Reorganization Contributes to Neural Tube Defects with Folate Deficiency
Source: Adv Sci (Weinh). 2025 Sep 18;12(47):e10603. doi: 10.1002/advs.202410603 (PMC12713105; doi:10.1002/advs.202410603)
Supplement: Supplementary file 1 — Supplemental Figures 1–10 [file ADVS-12-e10603-s001.pptx]

## Slide 1
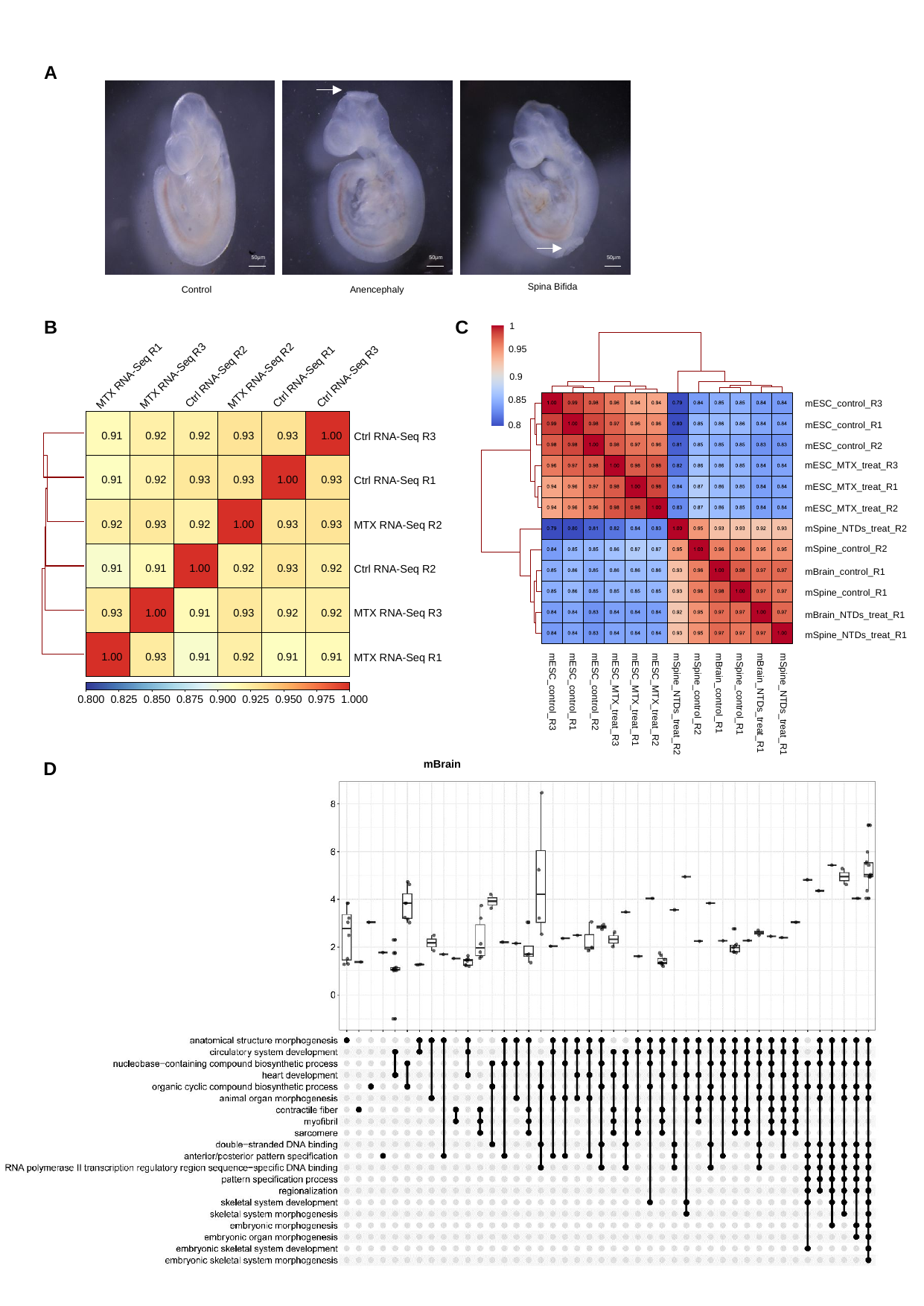

A
50μm
50μm
50μm
Spina Bifida
Control
Anencephaly
B
C
1
0.95
0.9
0.85
mESC_control_R3
mESC_control_R1
0.8
mESC_control_R2
mESC_MTX_treat_R3
mESC_MTX_treat_R1
mESC_MTX_treat_R2
mSpine_NTDs_treat_R2
mSpine_control_R2
mBrain_control_R1
mSpine_control_R1
mBrain_NTDs_treat_R1
mSpine_NTDs_treat_R1
mESC_control_R3
mESC_control_R1
mESC_control_R2
mBrain_control_R1
mSpine_control_R2
mSpine_control_R1
mESC_MTX_treat_R3
mESC_MTX_treat_R1
mESC_MTX_treat_R2
mBrain_NTDs_treat_R1
mSpine_NTDs_treat_R2
mSpine_NTDs_treat_R1
MTX RNA-Seq R1
MTX RNA-Seq R3
MTX RNA-Seq R2
Ctrl RNA-Seq R2
Ctrl RNA-Seq R1
Ctrl RNA-Seq R3
0.91
0.92
0.92
0.93
0.93
1.00
Ctrl RNA-Seq R3
Ctrl RNA-Seq R1
MTX RNA-Seq R2
Ctrl RNA-Seq R2
MTX RNA-Seq R3
MTX RNA-Seq R1
0.91
0.92
0.93
0.93
1.00
0.93
0.92
0.93
0.92
1.00
0.93
0.93
0.91
0.91
1.00
0.92
0.93
0.92
0.93
1.00
0.91
0.93
0.92
0.92
1.00
0.93
0.91
0.92
0.91
0.91
0.800
0.825
0.850
0.875
0.900
0.925
0.950
0.975
1.000
D
mBrain

## Slide 2
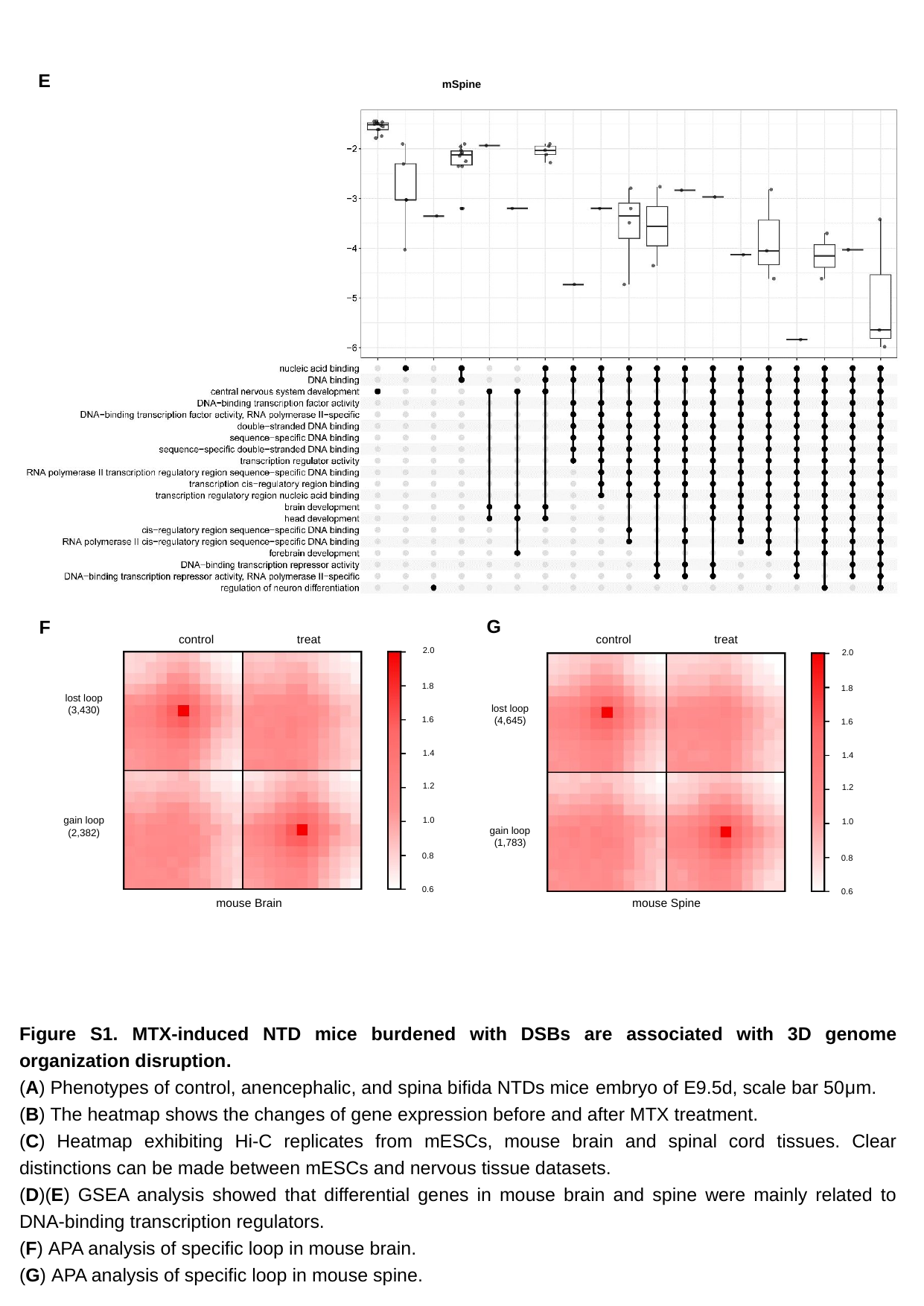

E
mSpine
G
F
control
treat
2.0
1.8
lost loop
(3,430)
1.6
1.4
1.2
gain loop
(2,382)
1.0
0.8
0.6
mouse Brain
control
treat
2.0
1.8
lost loop
(4,645)
1.6
1.4
1.2
1.0
gain loop
(1,783)
0.8
0.6
mouse Spine
Figure S1. MTX-induced NTD mice burdened with DSBs are associated with 3D genome organization disruption.
(A) Phenotypes of control, anencephalic, and spina bifida NTDs mice embryo of E9.5d, scale bar 50μm.
(B) The heatmap shows the changes of gene expression before and after MTX treatment.
(C) Heatmap exhibiting Hi-C replicates from mESCs, mouse brain and spinal cord tissues. Clear distinctions can be made between mESCs and nervous tissue datasets.
(D)(E) GSEA analysis showed that differential genes in mouse brain and spine were mainly related to DNA-binding transcription regulators.
(F) APA analysis of specific loop in mouse brain.
(G) APA analysis of specific loop in mouse spine.

## Slide 3
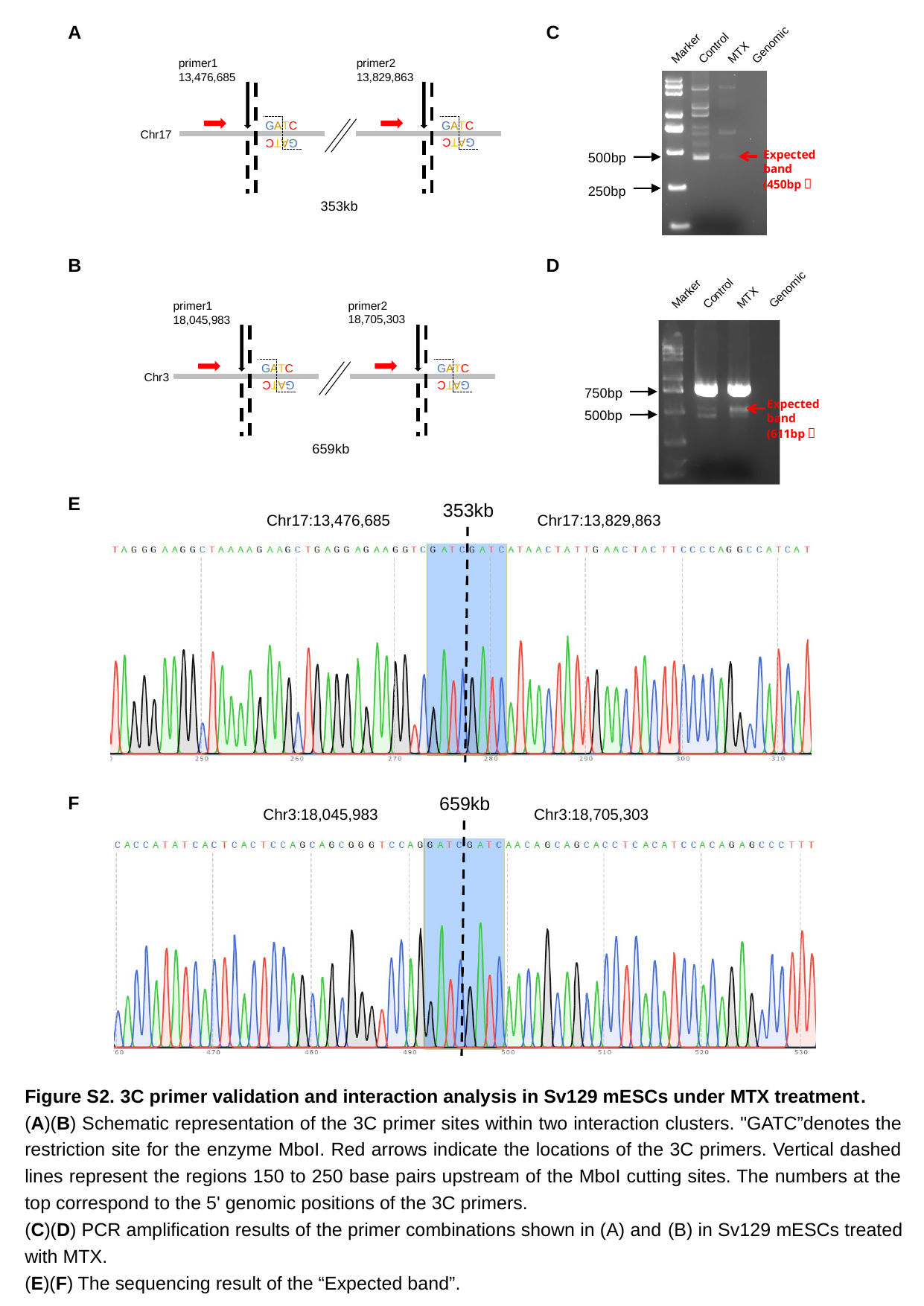

MTX
Control
Genomic
Marker
500bp
250bp
Expected
band (450bp）
C
A
primer1
13,476,685
primer2 13,829,863
GATC
GATC
Chr17
GATC
GATC
353kb
B
D
MTX
Control
Genomic
Marker
750bp
500bp
Expected
band (611bp）
primer2 18,705,303
primer1
18,045,983
GATC
GATC
Chr3
GATC
GATC
659kb
E
353kb
Chr17:13,476,685
Chr17:13,829,863
F
659kb
Chr3:18,705,303
Chr3:18,045,983
Figure S2. 3C primer validation and interaction analysis in Sv129 mESCs under MTX treatment.
(A)(B) Schematic representation of the 3C primer sites within two interaction clusters. "GATC”denotes the restriction site for the enzyme MboI. Red arrows indicate the locations of the 3C primers. Vertical dashed lines represent the regions 150 to 250 base pairs upstream of the MboI cutting sites. The numbers at the top correspond to the 5' genomic positions of the 3C primers.
(C)(D) PCR amplification results of the primer combinations shown in (A) and (B) in Sv129 mESCs treated with MTX.
(E)(F) The sequencing result of the “Expected band”.

## Slide 4
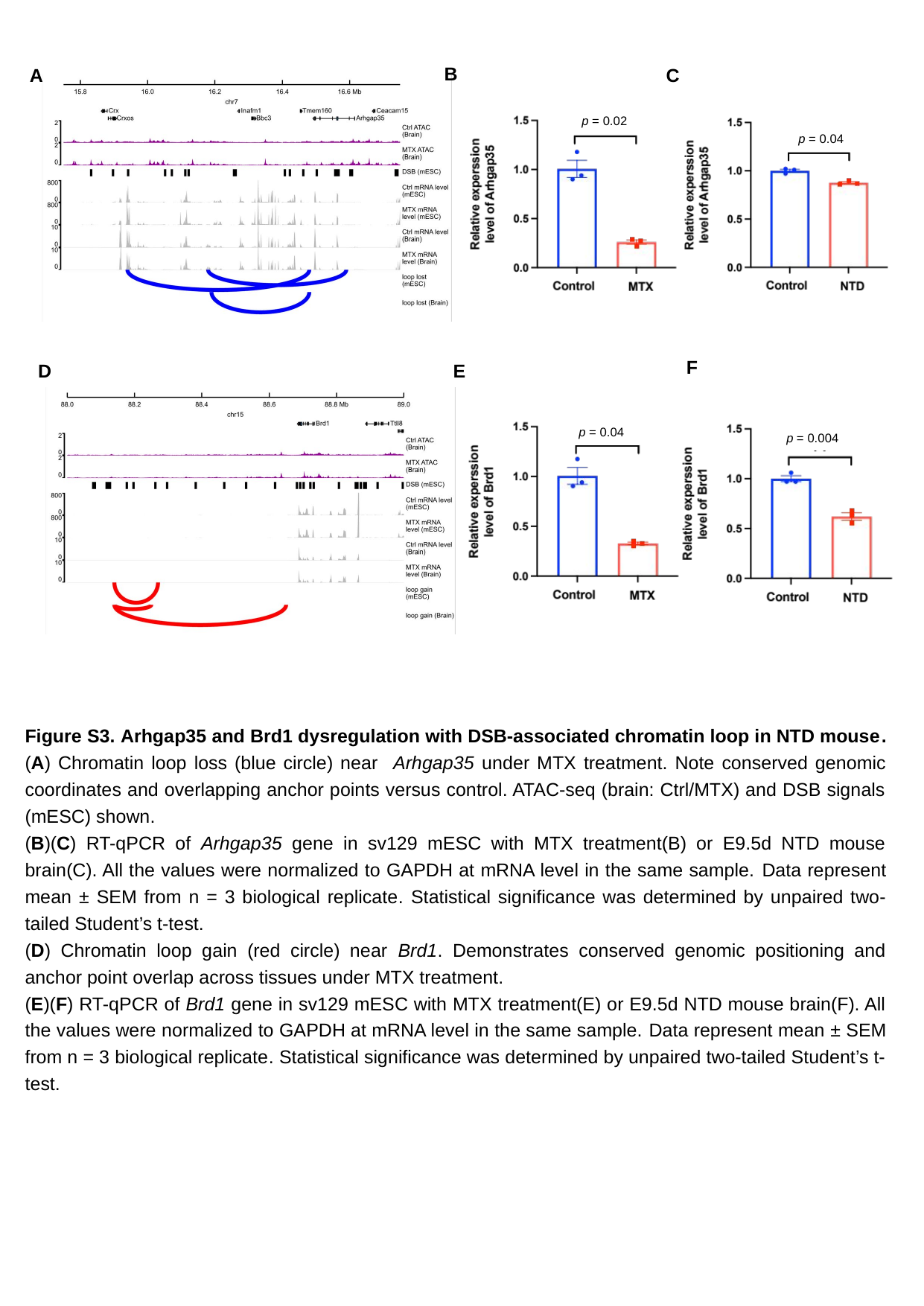

B
A
C
p = 0.02
p = 0.04
F
D
E
p = 0.04
p = 0.004
Figure S3. Arhgap35 and Brd1 dysregulation with DSB-associated chromatin loop in NTD mouse.
(A) Chromatin loop loss (blue circle) near Arhgap35 under MTX treatment. Note conserved genomic coordinates and overlapping anchor points versus control. ATAC-seq (brain: Ctrl/MTX) and DSB signals (mESC) shown.
(B)(C) RT-qPCR of Arhgap35 gene in sv129 mESC with MTX treatment(B) or E9.5d NTD mouse brain(C). All the values were normalized to GAPDH at mRNA level in the same sample. Data represent mean ± SEM from n = 3 biological replicate. Statistical significance was determined by unpaired two-tailed Student’s t-test.
(D) Chromatin loop gain (red circle) near Brd1. Demonstrates conserved genomic positioning and anchor point overlap across tissues under MTX treatment.
(E)(F) RT-qPCR of Brd1 gene in sv129 mESC with MTX treatment(E) or E9.5d NTD mouse brain(F). All the values were normalized to GAPDH at mRNA level in the same sample. Data represent mean ± SEM from n = 3 biological replicate. Statistical significance was determined by unpaired two-tailed Student’s t-test.

## Slide 5
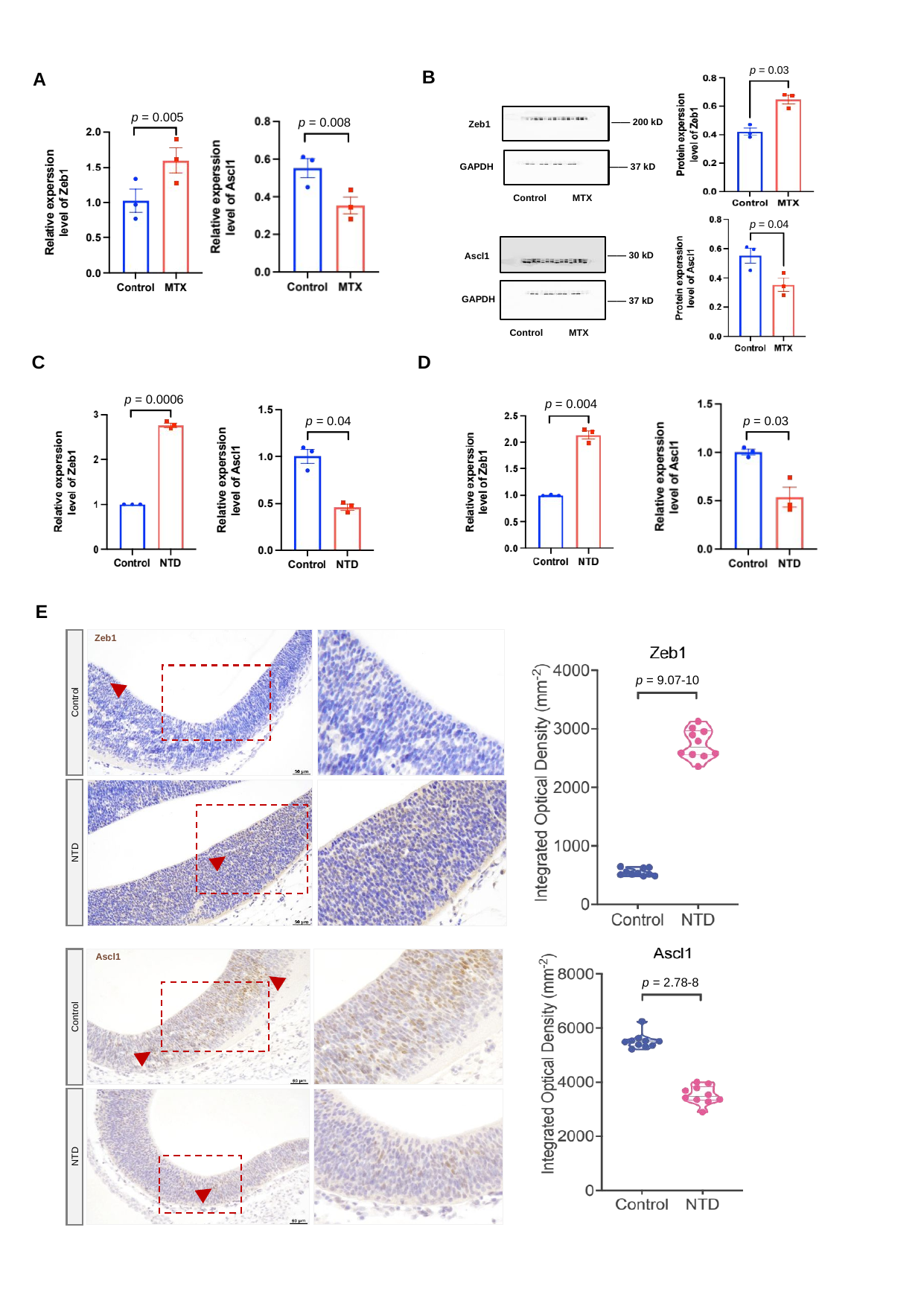

B
A
p = 0.03
—— 200 kD
Zeb1
GAPDH
—— 37 kD
Control MTX
Ascl1
GAPDH
—— 37 kD
Control MTX
—— 30 kD
p = 0.005
p = 0.008
p = 0.04
C
D
p = 0.0006
p = 0.03
p = 0.004
p = 0.04
E
Zeb1
Control
NTD
Ascl1
Control
NTD
p = 9.07-10
p = 2.78-8

## Slide 6
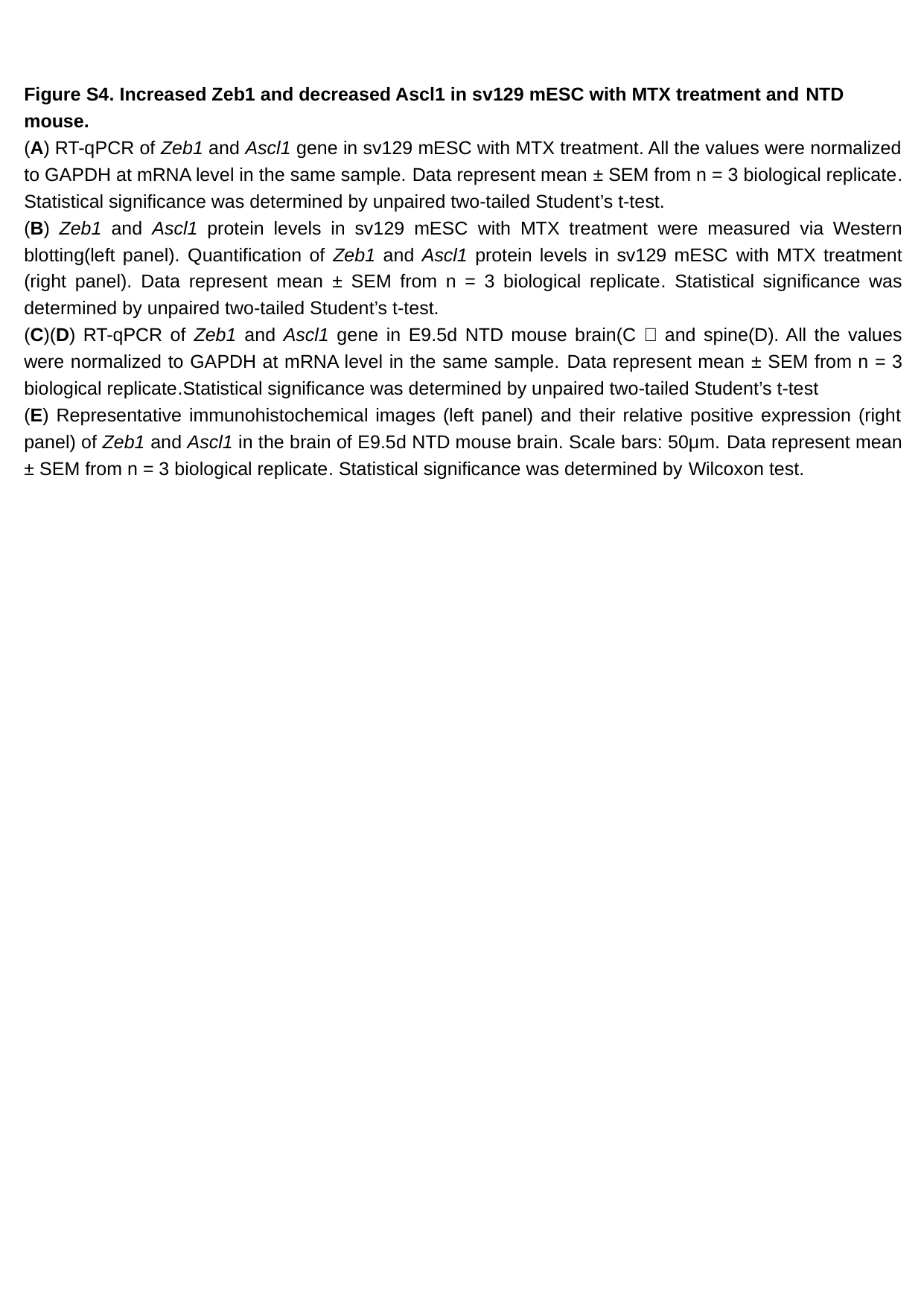

Figure S4. Increased Zeb1 and decreased Ascl1 in sv129 mESC with MTX treatment and NTD mouse.
(A) RT-qPCR of Zeb1 and Ascl1 gene in sv129 mESC with MTX treatment. All the values were normalized to GAPDH at mRNA level in the same sample. Data represent mean ± SEM from n = 3 biological replicate. Statistical significance was determined by unpaired two-tailed Student’s t-test.
(B) Zeb1 and Ascl1 protein levels in sv129 mESC with MTX treatment were measured via Western blotting(left panel). Quantification of Zeb1 and Ascl1 protein levels in sv129 mESC with MTX treatment (right panel). Data represent mean ± SEM from n = 3 biological replicate. Statistical significance was determined by unpaired two-tailed Student’s t-test.
(C)(D) RT-qPCR of Zeb1 and Ascl1 gene in E9.5d NTD mouse brain(C）and spine(D). All the values were normalized to GAPDH at mRNA level in the same sample. Data represent mean ± SEM from n = 3 biological replicate.Statistical significance was determined by unpaired two-tailed Student’s t-test
(E) Representative immunohistochemical images (left panel) and their relative positive expression (right panel) of Zeb1 and Ascl1 in the brain of E9.5d NTD mouse brain. Scale bars: 50μm. Data represent mean ± SEM from n = 3 biological replicate. Statistical significance was determined by ​​Wilcoxon test.

## Slide 7
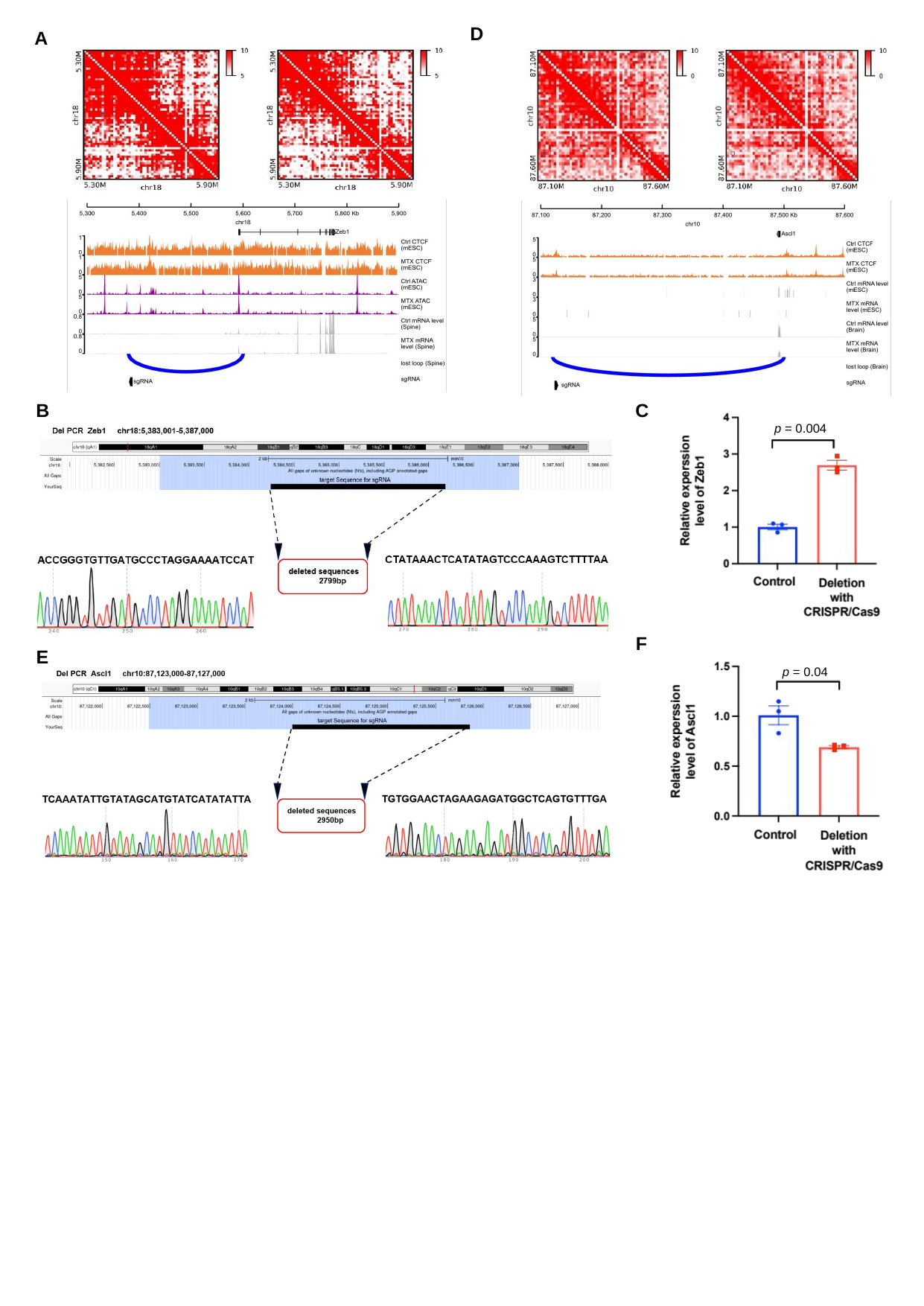

D
A
B
C
p = 0.004
F
E
p = 0.04

## Slide 8
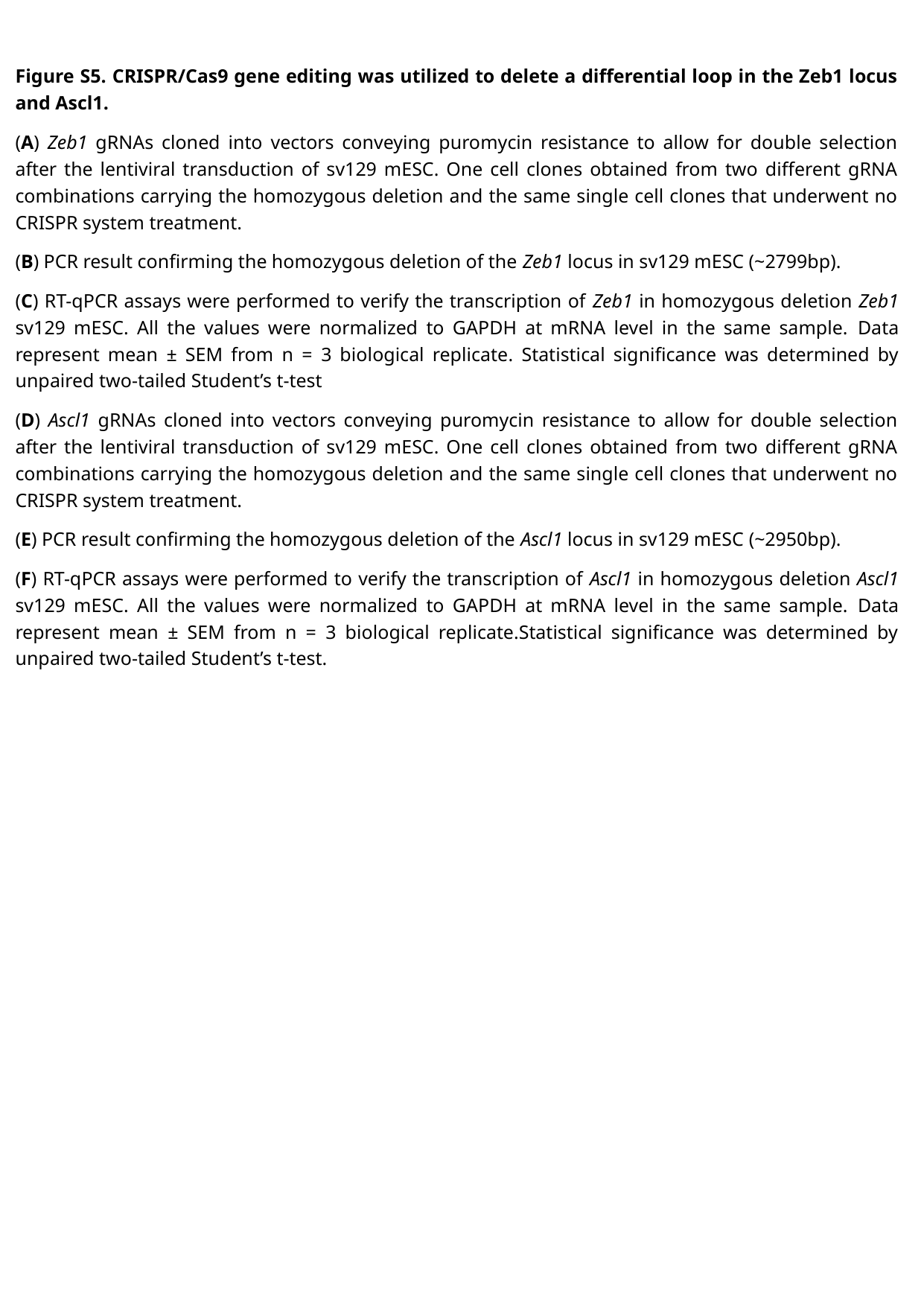

Figure S5. CRISPR/Cas9 gene editing was utilized to delete a differential loop in the Zeb1 locus and Ascl1.
(A) Zeb1 gRNAs cloned into vectors conveying puromycin resistance to allow for double selection after the lentiviral transduction of sv129 mESC. One cell clones obtained from two different gRNA combinations carrying the homozygous deletion and the same single cell clones that underwent no CRISPR system treatment.
(B) PCR result confirming the homozygous deletion of the Zeb1 locus in sv129 mESC (~2799bp).
(C) RT-qPCR assays were performed to verify the transcription of Zeb1 in homozygous deletion Zeb1 sv129 mESC. All the values were normalized to GAPDH at mRNA level in the same sample. Data represent mean ± SEM from n = 3 biological replicate. Statistical significance was determined by unpaired two-tailed Student’s t-test
(D) Ascl1 gRNAs cloned into vectors conveying puromycin resistance to allow for double selection after the lentiviral transduction of sv129 mESC. One cell clones obtained from two different gRNA combinations carrying the homozygous deletion and the same single cell clones that underwent no CRISPR system treatment.
(E) PCR result confirming the homozygous deletion of the Ascl1 locus in sv129 mESC (~2950bp).
(F) RT-qPCR assays were performed to verify the transcription of Ascl1 in homozygous deletion Ascl1 sv129 mESC. All the values were normalized to GAPDH at mRNA level in the same sample. Data represent mean ± SEM from n = 3 biological replicate.Statistical significance was determined by unpaired two-tailed Student’s t-test.

## Slide 9
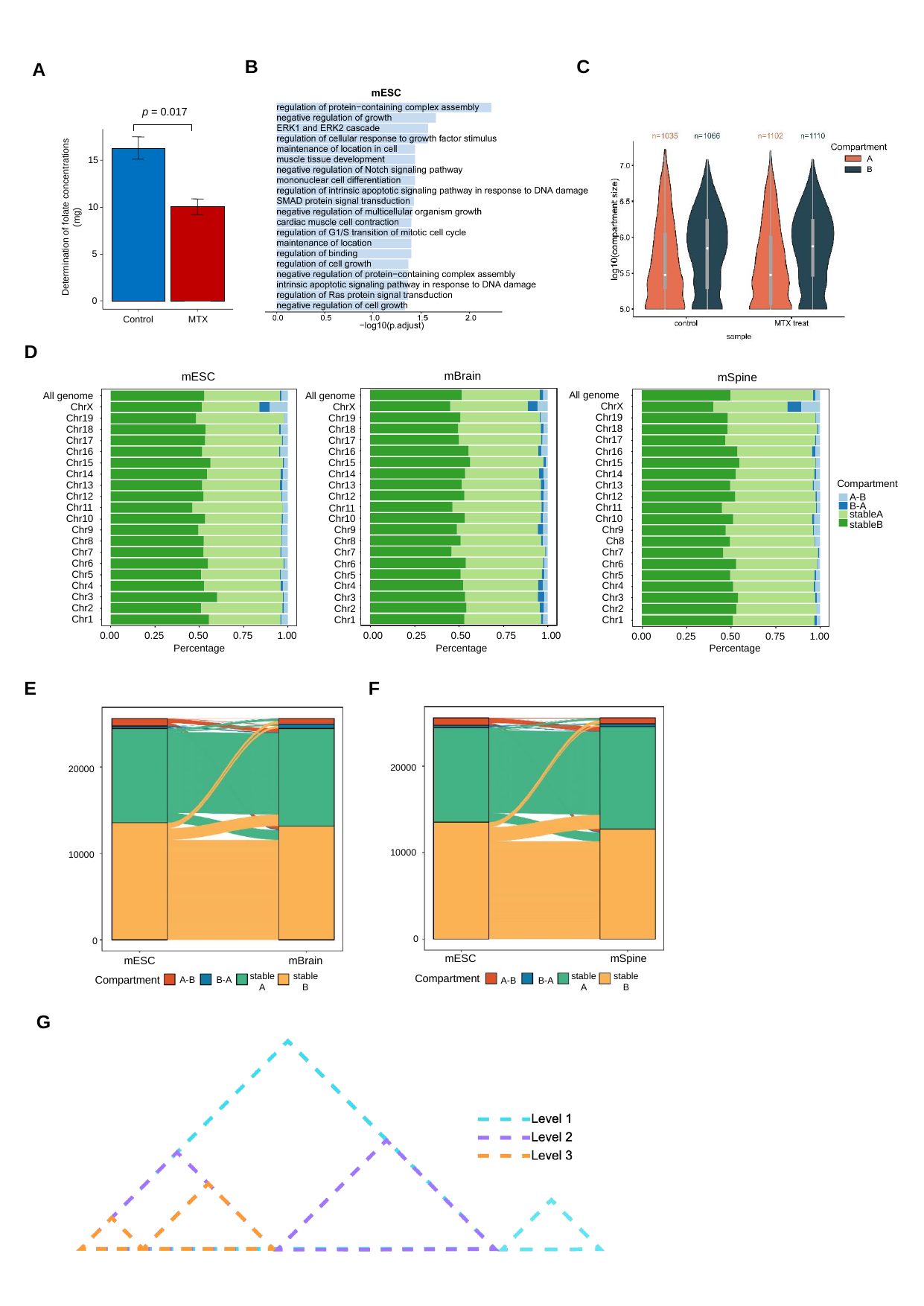

C
B
A
p = 0.017
15
10
Determination of folate concentrations
(mg)
5
0
Control
MTX
D
mBrain
mESC
mSpine
All genome
ChrX
Chr19
Chr18
Chr17
Chr16
Chr15
Chr14
Chr13
Chr12
Chr11
Chr10
Chr9
Chr8
Chr7
Chr6
Chr5
Chr4
Chr3
Chr2
Chr1
0.50
0.00
0.25
0.75
1.00
Percentage
All genome
ChrX
Chr19
Chr18
Chr17
Chr16
Chr15
Chr14
Compartment
Chr13
Chr12
A-B
B-A
Chr11
stableA
Chr10
stableB
Chr9
Ch8
Chr7
Chr6
Chr5
Chr4
Chr3
Chr2
Chr1
0.00
0.25
0.50
0.75
1.00
Percentage
All genome
ChrX
Chr19
Chr18
Chr17
Chr16
Chr15
Chr14
Chr13
Chr12
Chr11
Chr10
Chr9
Chr8
Chr7
Chr6
Chr5
Chr4
Chr3
Chr2
Chr1
0.25
0.50
0.75
1.00
0.00
Percentage
E
F
20000
10000
0
mESC
mSpine
stable A
stable B
Compartment
A-B
B-A
20000
10000
0
mESC
mBrain
stable A
stable B
Compartment
A-B
B-A
G

## Slide 10
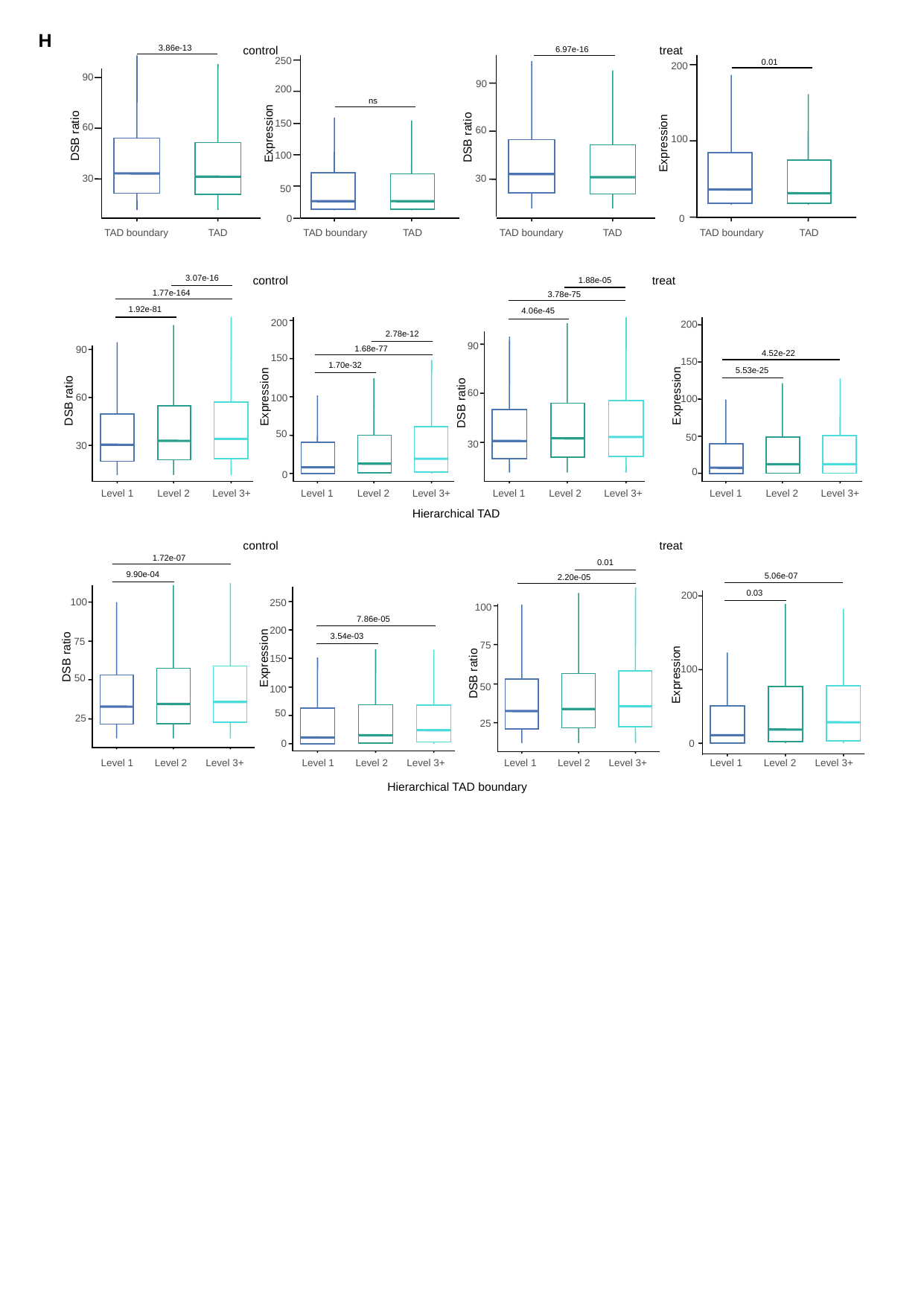

H
3.86e-13
6.97e-16
0.01
250
200
150
Expression
100
50
0
TAD boundary
TAD
90
60
DSB ratio
30
TAD boundary
TAD
90
60
DSB ratio
30
TAD boundary
TAD
200
100
Expression
0
TAD boundary
TAD
ns
control
treat
control
3.07e-16
1.88e-05
1.77e-164
3.78e-75
1.92e-81
4.06e-45
200
150
Expression
100
50
0
Level 1
Level 2
Level 3+
90
60
DSB ratio
30
Level 1
Level 2
Level 3+
90
60
DSB ratio
30
Level 1
Level 2
Level 3+
200
150
Expression
100
50
0
Level 1
Level 2
Level 3+
2.78e-12
1.68e-77
4.52e-22
1.70e-32
5.53e-25
Hierarchical TAD
treat
control
treat
1.72e-07
0.01
9.90e-04
5.06e-07
2.20e-05
0.03
200
100
250
100
200
75
75
DSB ratio
Expression
150
100
DSB ratio
Expression
50
50
100
50
25
25
0
0
Level 1
Level 2
Level 3+
Level 1
Level 3+
Level 1
Level 2
Level 3+
Level 1
Level 2
Level 3+
Level 2
7.86e-05
3.54e-03
Hierarchical TAD boundary

## Slide 11
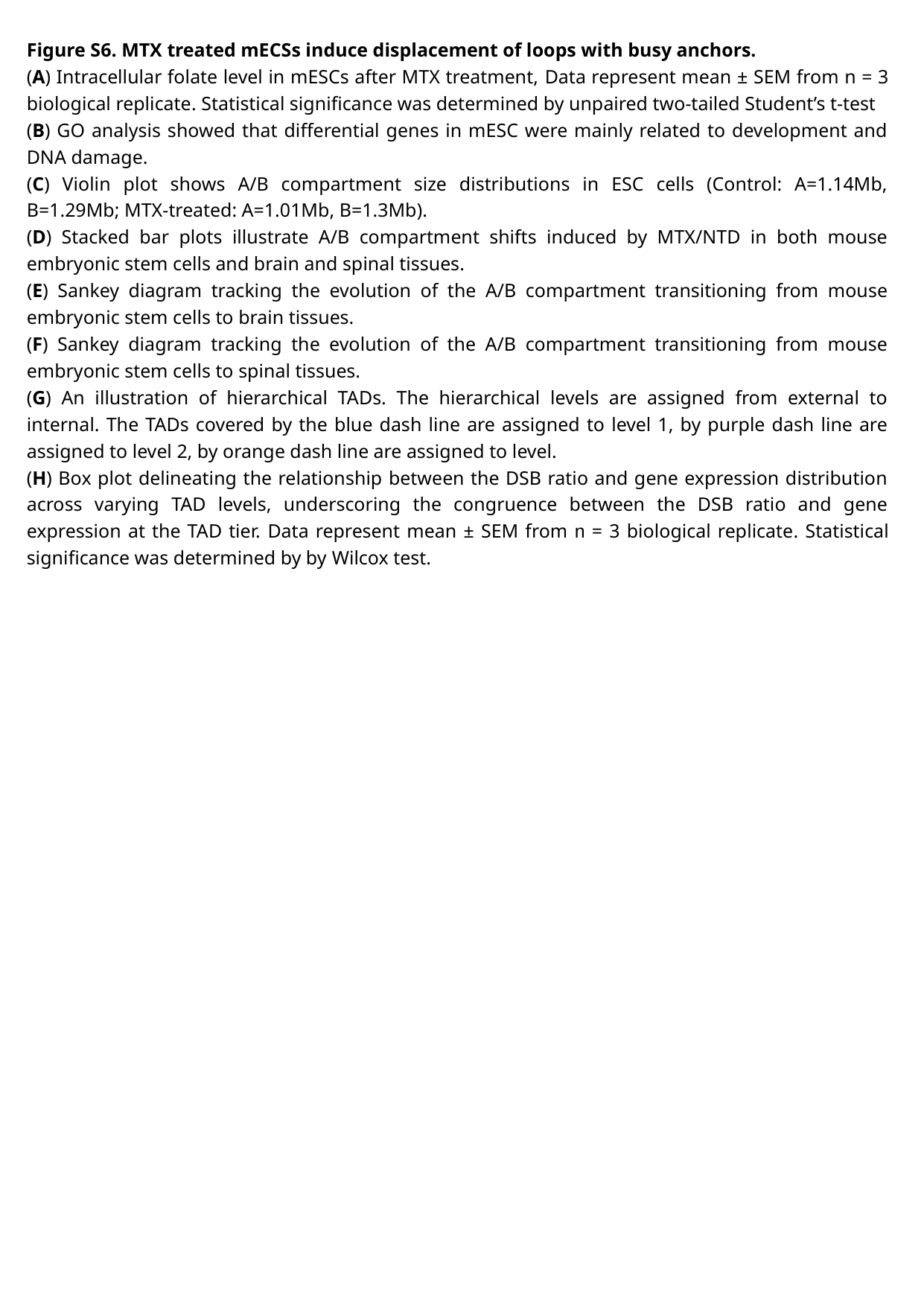

Figure S6. MTX treated mECSs induce displacement of loops with busy anchors.
(A) Intracellular folate level in mESCs after MTX treatment, Data represent mean ± SEM from n = 3 biological replicate. Statistical significance was determined by unpaired two-tailed Student’s t-test
​​(B) GO analysis showed that differential genes in mESC were mainly related to development and DNA damage.
(C) Violin plot shows A/B compartment size distributions in ESC cells (Control: A=1.14Mb, B=1.29Mb; MTX-treated: A=1.01Mb, B=1.3Mb).​
(D) Stacked bar plots illustrate A/B compartment shifts induced by MTX/NTD in both mouse embryonic stem cells and brain and spinal tissues.
(E) Sankey diagram tracking the evolution of the A/B compartment transitioning from mouse embryonic stem cells to brain tissues.
(F) Sankey diagram tracking the evolution of the A/B compartment transitioning from mouse embryonic stem cells to spinal tissues.
(G) An illustration of hierarchical TADs. The hierarchical levels are assigned from external to internal. The TADs covered by the blue dash line are assigned to level 1, by purple dash line are assigned to level 2, by orange dash line are assigned to level.
(H) Box plot delineating the relationship between the DSB ratio and gene expression distribution across varying TAD levels, underscoring the congruence between the DSB ratio and gene expression at the TAD tier. Data represent mean ± SEM from n = 3 biological replicate. Statistical significance was determined by by Wilcox test.

## Slide 12
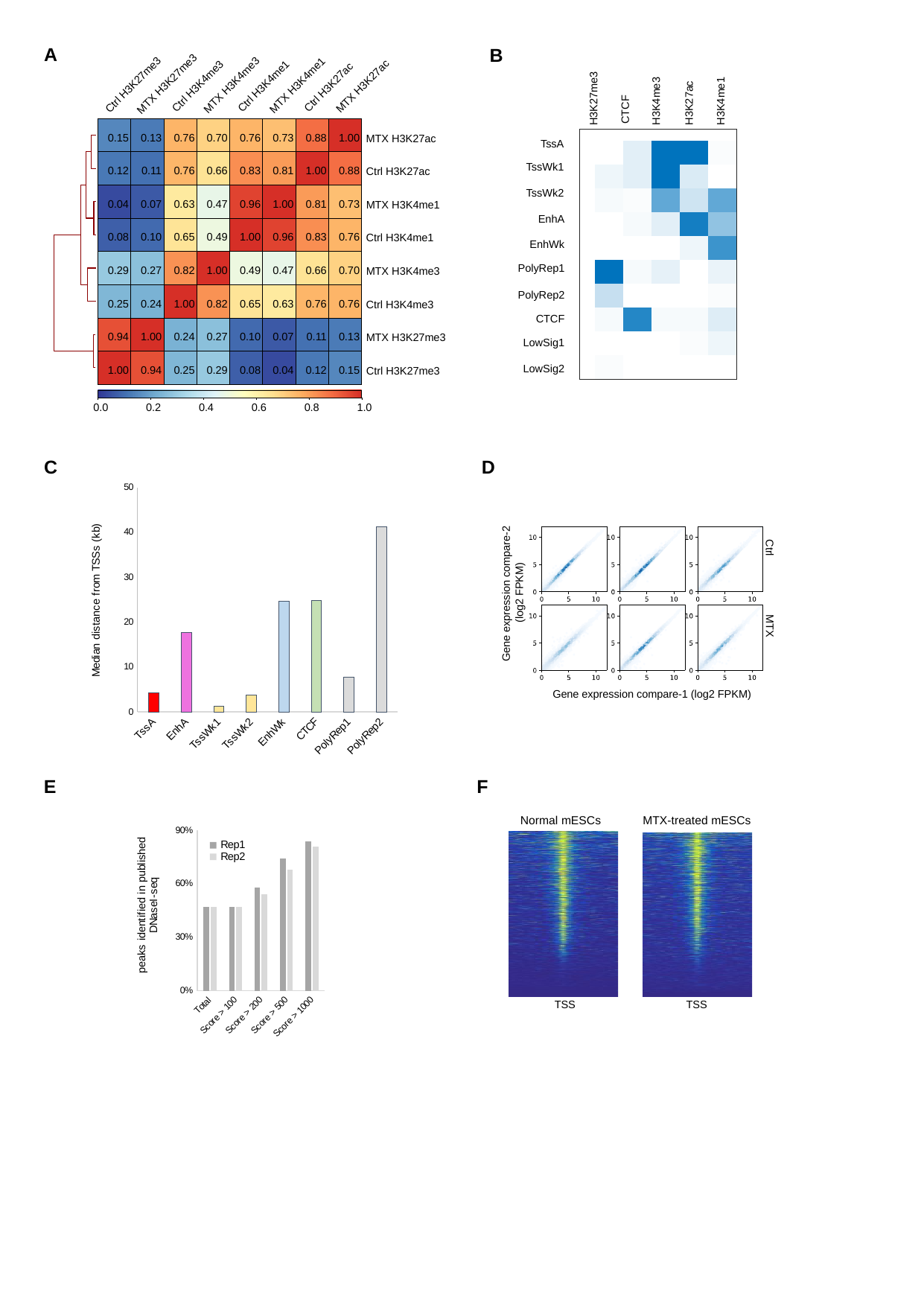

A
B
H3K27me3
H3K4me1
H3K4me3
H3K27ac
CTCF
TssA
TssWk1
TssWk2
EnhA
EnhWk
PolyRep1
PolyRep2
CTCF
LowSig1
LowSig2
MTX H3K27me3
Ctrl H3K27me3
MTX H3K4me3
MTX H3K4me1
Ctrl H3K4me3
Ctrl H3K4me1
MTX H3K27ac
Ctrl H3K27ac
0.15
0.13
0.76
0.70
0.76
0.73
0.88
1.00
MTX H3K27ac
Ctrl H3K27ac
MTX H3K4me1
Ctrl H3K4me1
MTX H3K4me3
Ctrl H3K4me3
MTX H3K27me3
Ctrl H3K27me3
0.12
0.11
0.76
0.66
0.83
0.81
1.00
0.88
0.04
0.07
0.63
0.47
0.96
1.00
0.81
0.73
0.08
0.10
0.65
0.49
1.00
0.96
0.83
0.76
0.29
0.27
0.82
1.00
0.49
0.47
0.66
0.70
0.25
0.24
1.00
0.82
0.65
0.63
0.76
0.76
0.94
1.00
0.24
0.27
0.10
0.07
0.11
0.13
1.00
0.94
0.25
0.29
0.08
0.04
0.12
0.15
0.0
0.2
0.4
0.6
0.8
1.0
C
D
### Chart
| Category | |
|---|---|
| TssA | 4.286 |
| EnhA | 17.654 |
| TssWk1 | 1.339 |
| TssWk2 | 3.725 |
| EnhWk | 24.7495 |
| CTCF | 24.8725 |
| PolyRep1 | 7.748 |
| PolyRep2 | 41.222 |
Ctrl
Gene expression compare-2
 (log2 FPKM)
MTX
Gene expression compare-1 (log2 FPKM)
E
F
Normal mESCs
MTX-treated mESCs
TSS
TSS
### Chart
| Category | Rep1 | Rep2 |
|---|---|---|
| Total | 0.47 | 0.47 |
| Score > 100 | 0.47 | 0.47 |
| Score > 200 | 0.58 | 0.54 |
| Score > 500 | 0.74 | 0.68 |
| Score > 1000 | 0.84 | 0.81 |

## Slide 13
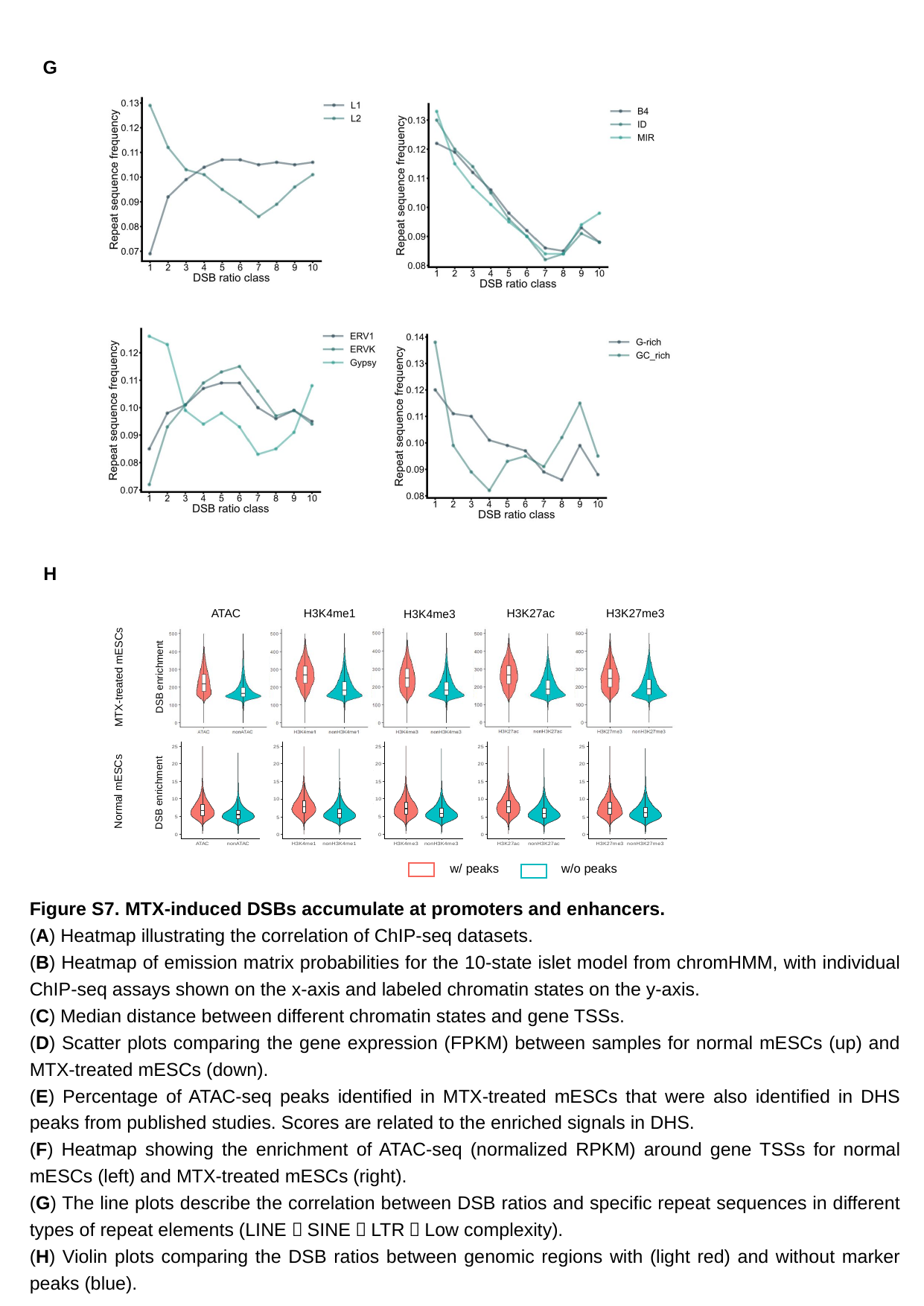

G
H
H3K27ac
H3K27me3
ATAC
H3K4me1
H3K4me3
MTX-treated mESCs
DSB enrichment
Normal mESCs
DSB enrichment
w/ peaks
w/o peaks
Figure S7. MTX-induced DSBs accumulate at promoters and enhancers.
(A) Heatmap illustrating the correlation of ChIP-seq datasets.
(B) Heatmap of emission matrix probabilities for the 10-state islet model from chromHMM, with individual ChIP-seq assays shown on the x-axis and labeled chromatin states on the y-axis.
(C) Median distance between different chromatin states and gene TSSs.
(D) Scatter plots comparing the gene expression (FPKM) between samples for normal mESCs (up) and MTX-treated mESCs (down).
(E) Percentage of ATAC-seq peaks identified in MTX-treated mESCs that were also identified in DHS peaks from published studies. Scores are related to the enriched signals in DHS.
(F) Heatmap showing the enrichment of ATAC-seq (normalized RPKM) around gene TSSs for normal mESCs (left) and MTX-treated mESCs (right).
(G) The line plots describe the correlation between DSB ratios and specific repeat sequences in different types of repeat elements (LINE，SINE，LTR，Low complexity).
(H) Violin plots comparing the DSB ratios between genomic regions with (light red) and without marker peaks (blue).

## Slide 14
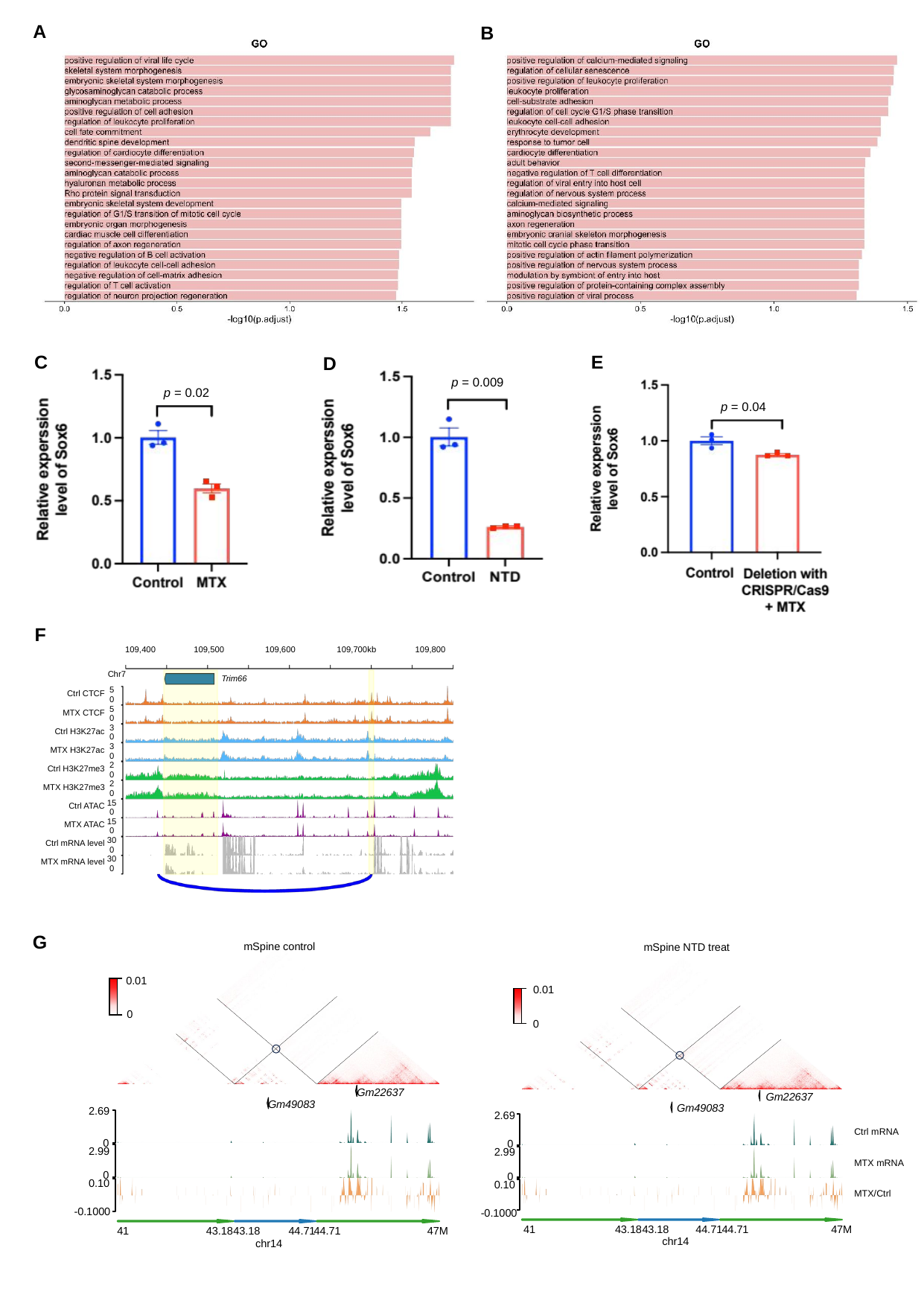

A
B
C
E
D
p = 0.02
p = 0.009
p = 0.04
F
109,400
109,500
109,600
109,700kb
109,800
Chr7
5
0
5
0
3
0
3
0
2
0
2
0
15
0
15
0
30
0
30
0
Trim66
Ctrl CTCF
MTX CTCF
Ctrl H3K27ac
MTX H3K27ac
Ctrl H3K27me3
MTX H3K27me3
Ctrl ATAC
MTX ATAC
Ctrl mRNA level
MTX mRNA level
G
mSpine control
0.01
0
Gm22637
Gm49083
2.69
0
2.99
0
0.10
-0.1000
41
43.18
43.18
44.71
44.71
47M
chr14
mSpine NTD treat
0.01
0
Gm22637
Gm49083
2.69
0
2.99
0
0.10
-0.1000
41
43.18
43.18
44.71
44.71
47M
chr14
Ctrl mRNA
MTX mRNA
MTX/Ctrl

## Slide 15
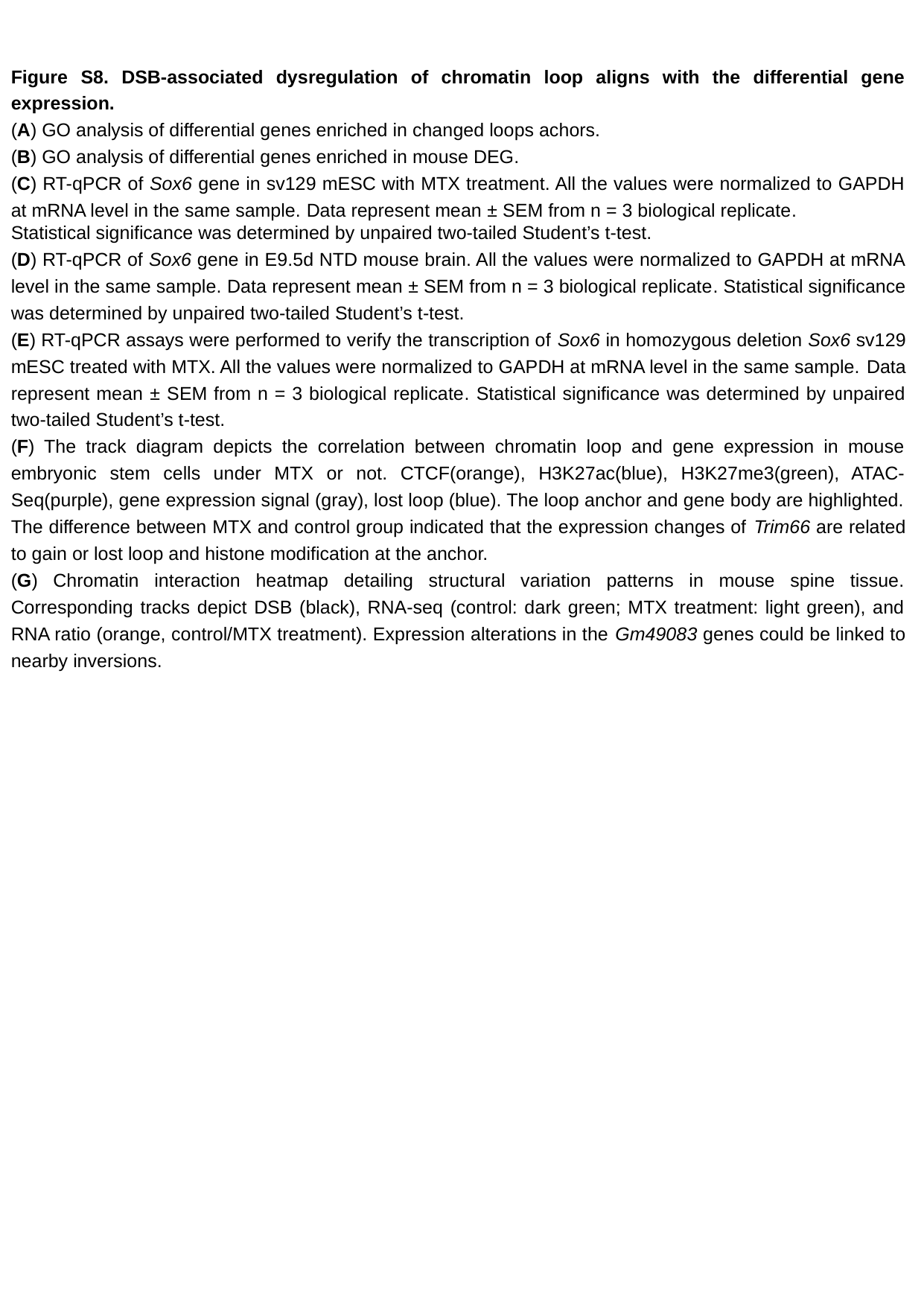

Figure S8. DSB-associated dysregulation of chromatin loop aligns with the differential gene expression.
(A) GO analysis of differential genes enriched in changed loops achors.
(B) GO analysis of differential genes enriched in mouse DEG.
(C) RT-qPCR of Sox6 gene in sv129 mESC with MTX treatment. All the values were normalized to GAPDH at mRNA level in the same sample. Data represent mean ± SEM from n = 3 biological replicate.
Statistical significance was determined by unpaired two-tailed Student’s t-test.
(D) RT-qPCR of Sox6 gene in E9.5d NTD mouse brain. All the values were normalized to GAPDH at mRNA level in the same sample. Data represent mean ± SEM from n = 3 biological replicate. Statistical significance was determined by unpaired two-tailed Student’s t-test.
(E) RT-qPCR assays were performed to verify the transcription of Sox6 in homozygous deletion Sox6 sv129 mESC treated with MTX. All the values were normalized to GAPDH at mRNA level in the same sample. Data represent mean ± SEM from n = 3 biological replicate. Statistical significance was determined by unpaired two-tailed Student’s t-test.
(F) The track diagram depicts the correlation between chromatin loop and gene expression in mouse embryonic stem cells under MTX or not. CTCF(orange), H3K27ac(blue), H3K27me3(green), ATAC-Seq(purple), gene expression signal (gray), lost loop (blue). The loop anchor and gene body are highlighted. The difference between MTX and control group indicated that the expression changes of Trim66 are related to gain or lost loop and histone modification at the anchor.
(G) Chromatin interaction heatmap detailing structural variation patterns in mouse spine tissue. Corresponding tracks depict DSB (black), RNA-seq (control: dark green; MTX treatment: light green), and RNA ratio (orange, control/MTX treatment). Expression alterations in the Gm49083 genes could be linked to nearby inversions.

## Slide 16
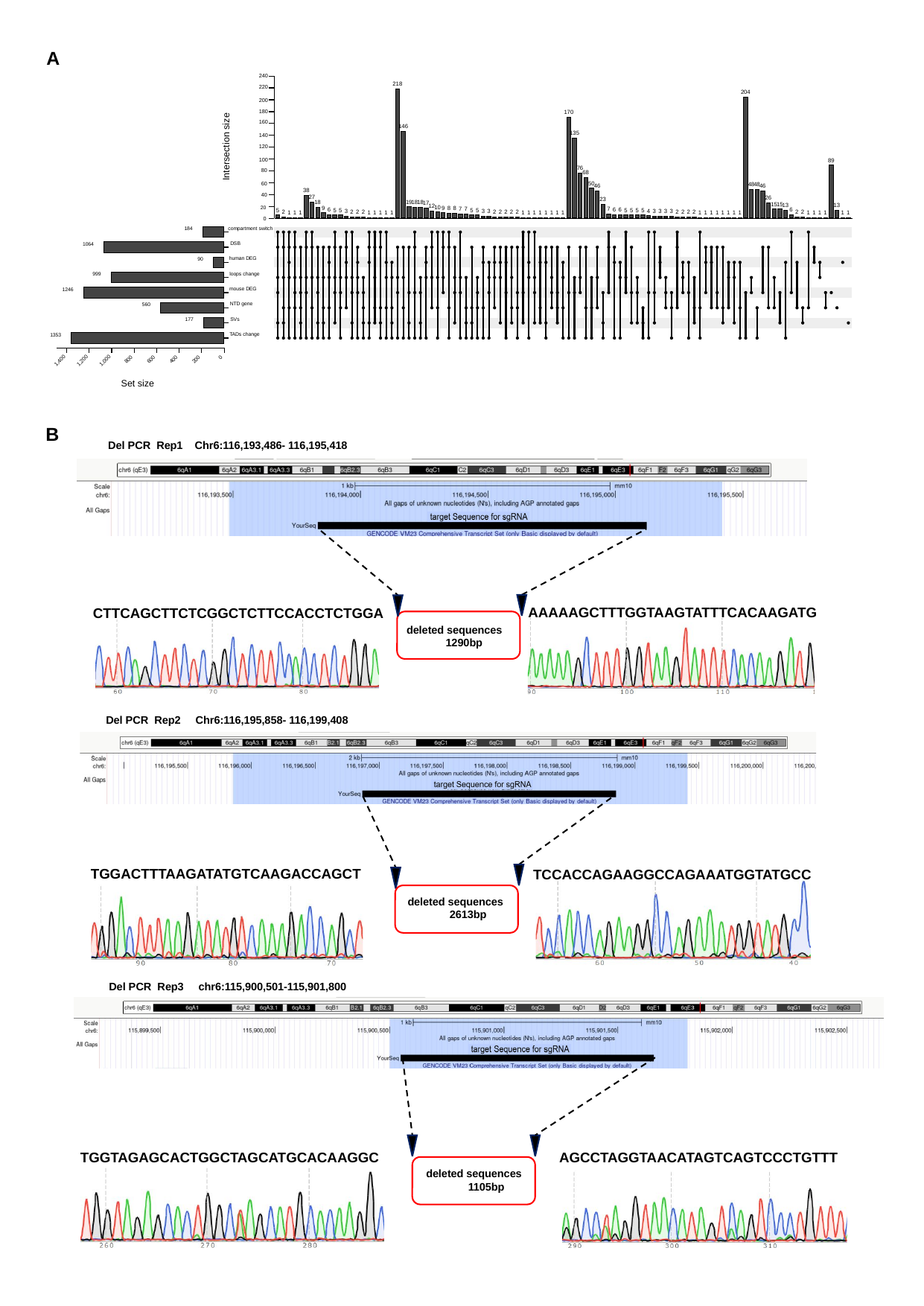

A
240
220
200
180
160
140
Intersection size
120
100
80
60
40
20
0
218
204
170
146
135
89
76
68
50
48
48
46
46
38
27
26
23
19
18
18
18
17
15
15
13
13
12
10
9
9
8
8
7
7
7
6
6
6
6
5
5
5
5
5
5
5
5
5
4
3
3
3
3
3
3
3
2
2
2
2
2
2
2
2
2
2
2
2
2
2
2
1
1
1
1
1
1
1
1
1
1
1
1
1
1
1
1
1
1
1
1
1
1
1
1
1
1
1
1
1
1
184
1064
90
999
1246
560
177
1353
compartment switch
DSB
human DEG
loops change
mouse DEG
NTD gene
SVs
TADs change
1,400
1,200
1,000
800
600
400
200
0
Set size
B
Del PCR Rep1 Chr6:116,193,486- 116,195,418
CTTCAGCTTCTCGGCTCTTCCACCTCTGGA
 deleted sequences 1290bp
AAAAAGCTTTGGTAAGTATTTCACAAGATG
Del PCR Rep2 Chr6:116,195,858- 116,199,408
TGGACTTTAAGATATGTCAAGACCAGCT
 TCCACCAGAAGGCCAGAAATGGTATGCC
deleted sequences 2613bp
Del PCR Rep3 chr6:115,900,501-115,901,800
TGGTAGAGCACTGGCTAGCATGCACAAGGC
deleted sequences 1105bp
AGCCTAGGTAACATAGTCAGTCCCTGTTT

## Slide 17
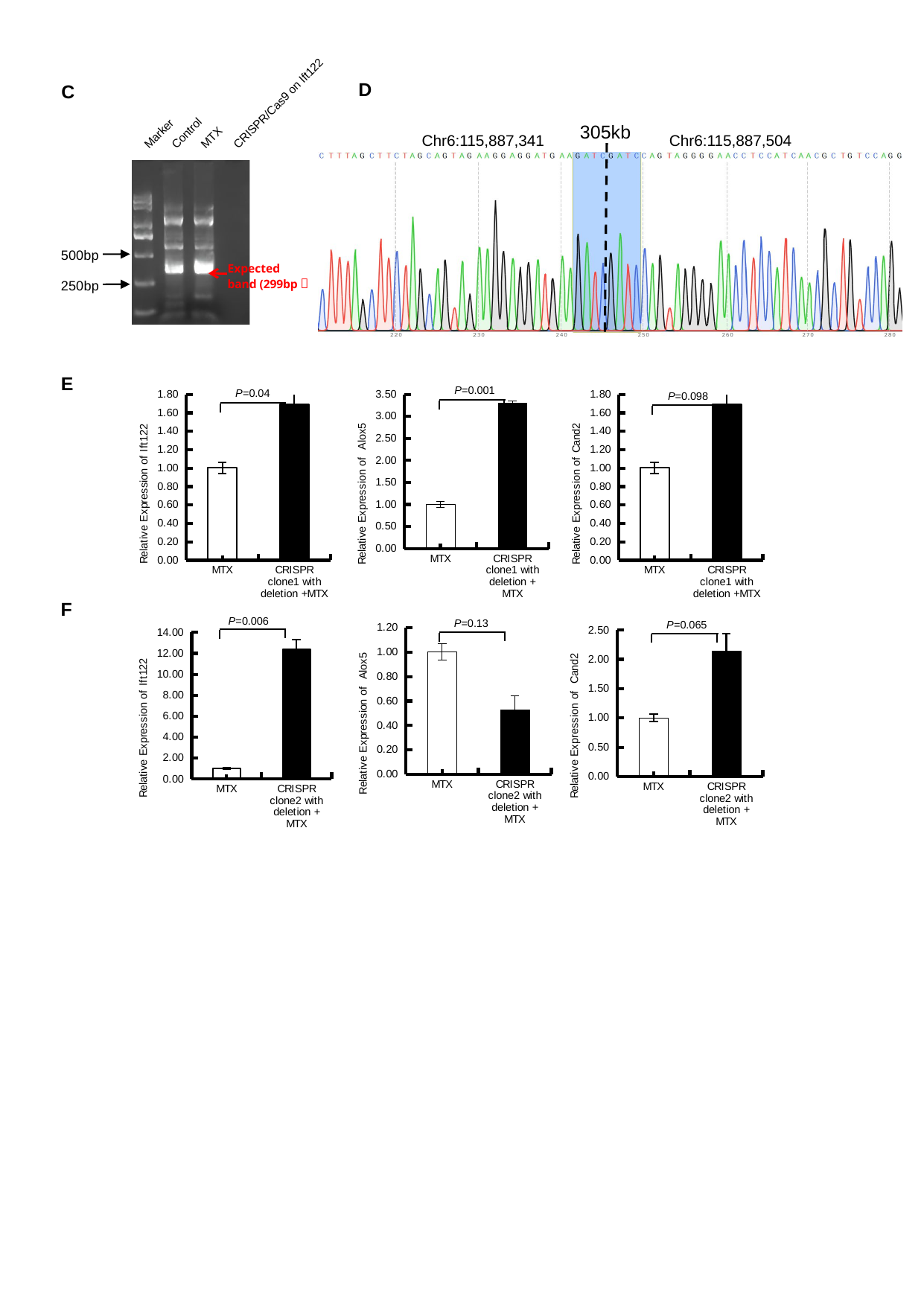

D
C
CRISPR/Cas9 on Ift122
MTX
Control
Marker
500bp
250bp
Expected
band (299bp）
305kb
Chr6:115,887,341
Chr6:115,887,504
P=0.006
P=0.13
P=0.065
### Chart
| Category | |
|---|---|
| MTX | 1.00488569353858 |
| CRISPR clone2 with deletion + MTX | 0.527349375495811 |
### Chart
| Category | |
|---|---|
| MTX | 1.00400131214665 |
| CRISPR clone2 with deletion + MTX | 2.14400801299338 |
### Chart
| Category | |
|---|---|
| MTX | 1.00915419285264 |
| CRISPR clone2 with deletion + MTX | 12.3854038549148 |
P=0.001
P=0.04
P=0.098
### Chart
| Category | |
|---|---|
| MTX | 1.00488569353858 |
| CRISPR clone1 with deletion + MTX | 3.30711633192282 |
### Chart
| Category | |
|---|---|
| MTX | 1.00400131214665 |
| CRISPR clone1 with deletion +MTX | 1.6952268219268 |
### Chart
| Category | |
|---|---|
| MTX | 1.00400131214665 |
| CRISPR clone1 with deletion +MTX | 1.6952268219268 |
### Chart
| Category |
|---|
E
F

## Slide 18
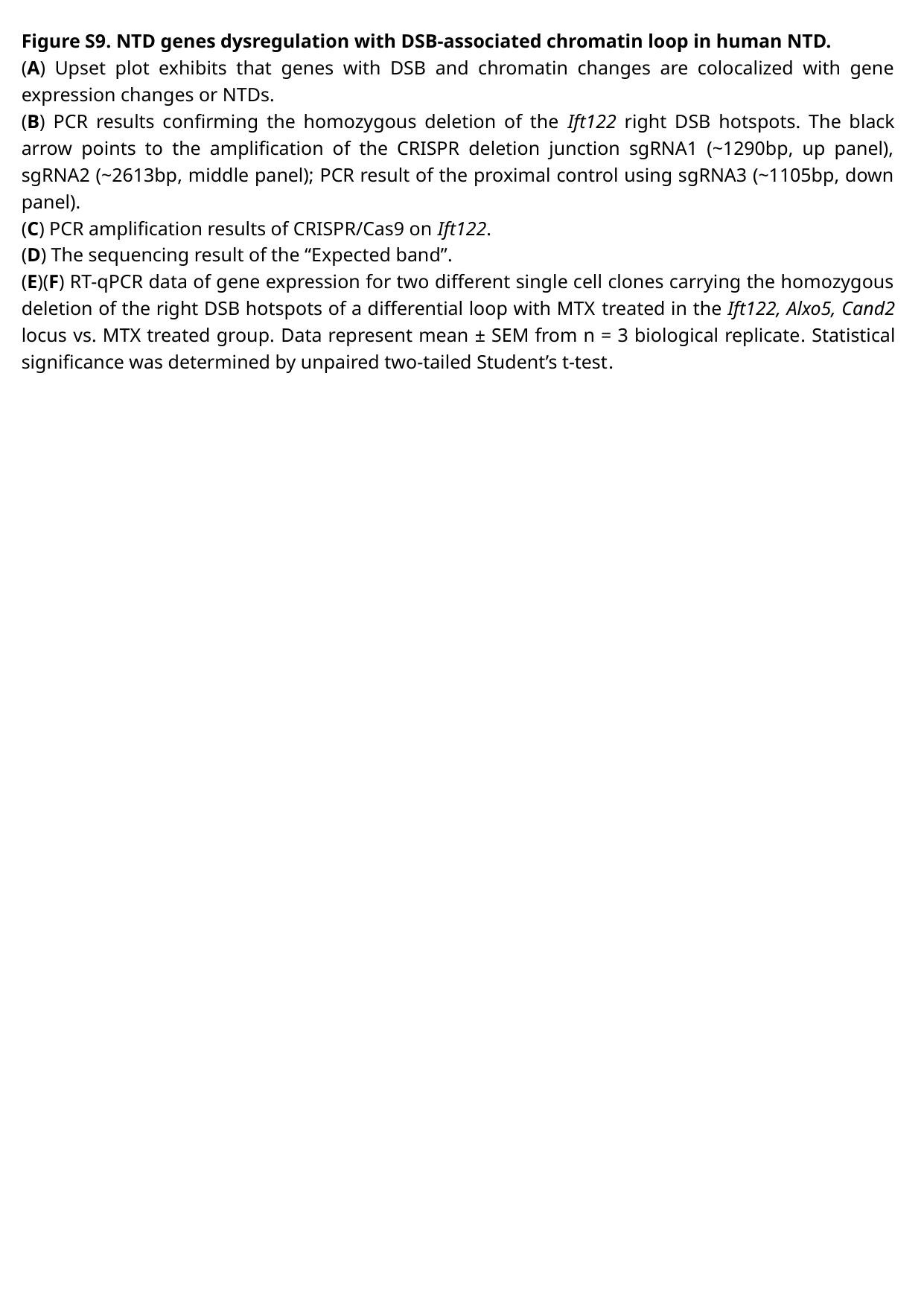

Figure S9. NTD genes dysregulation with DSB-associated chromatin loop in human NTD.
(A) Upset plot exhibits that genes with DSB and chromatin changes are colocalized with gene expression changes or NTDs.
(B) PCR results confirming the homozygous deletion of the Ift122 right DSB hotspots. The black arrow points to the amplification of the CRISPR deletion junction sgRNA1 (~1290bp, up panel), sgRNA2 (~2613bp, middle panel); PCR result of the proximal control using sgRNA3 (~1105bp, down panel).
(C) PCR amplification results of CRISPR/Cas9 on Ift122.
(D) The sequencing result of the “Expected band”.
(E)(F) RT-qPCR data of gene expression for two different single cell clones carrying the homozygous deletion of the right DSB hotspots of a differential loop with MTX treated in the Ift122, Alxo5, Cand2 locus vs. MTX treated group. Data represent mean ± SEM from n = 3 biological replicate. Statistical significance was determined by unpaired two-tailed Student’s t-test.

## Slide 19
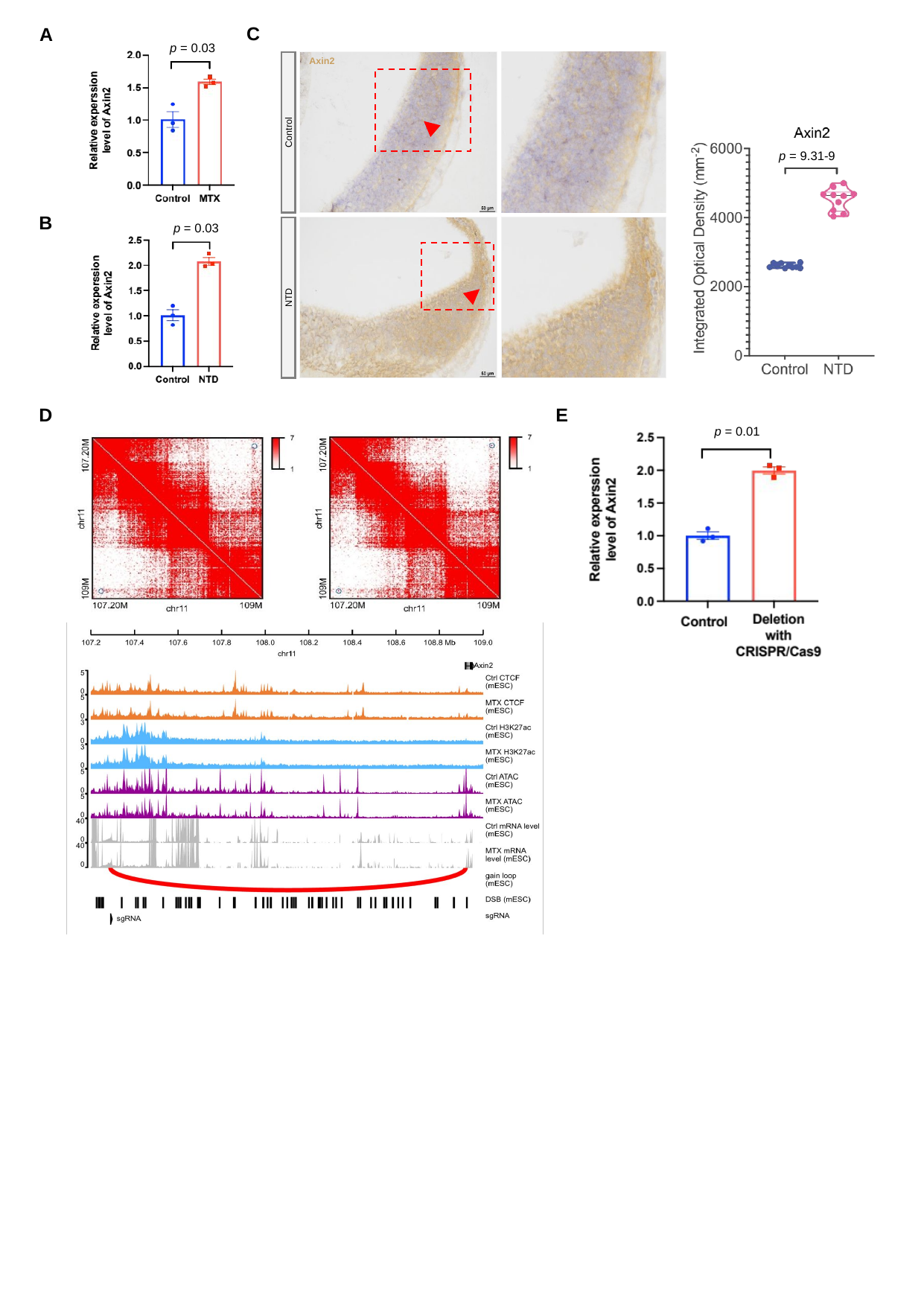

C
A
p = 0.03
Axin2
Control
NTD
p = 9.31-9
B
p = 0.03
E
D
p = 0.01

## Slide 20
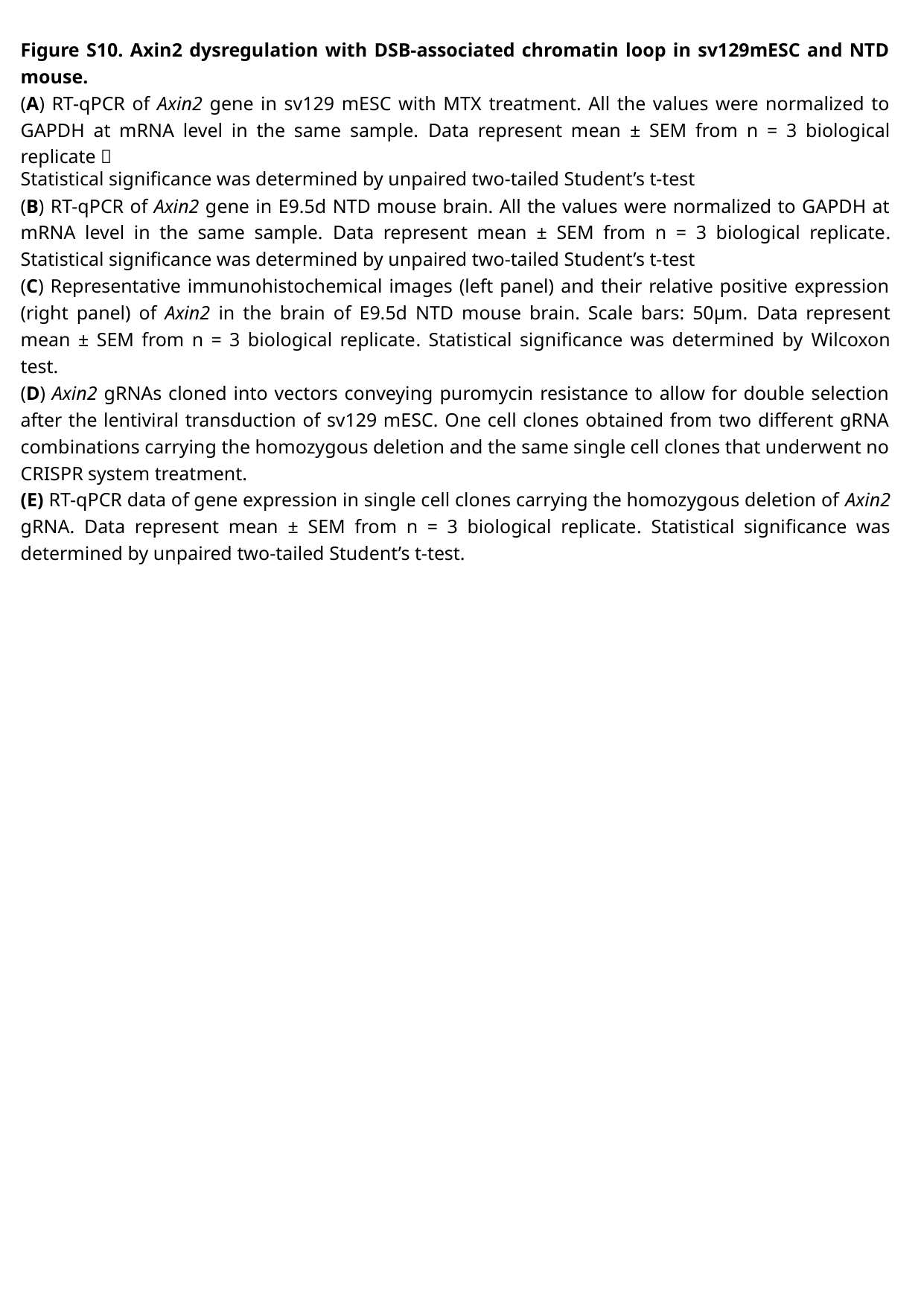

Figure S10. Axin2 dysregulation with DSB-associated chromatin loop in sv129mESC and NTD mouse.
(A) RT-qPCR of Axin2 gene in sv129 mESC with MTX treatment. All the values were normalized to GAPDH at mRNA level in the same sample. Data represent mean ± SEM from n = 3 biological replicate，
Statistical significance was determined by unpaired two-tailed Student’s t-test
(B) RT-qPCR of Axin2 gene in E9.5d NTD mouse brain. All the values were normalized to GAPDH at mRNA level in the same sample. Data represent mean ± SEM from n = 3 biological replicate. Statistical significance was determined by unpaired two-tailed Student’s t-test
(C) Representative immunohistochemical images (left panel) and their relative positive expression (right panel) of Axin2 in the brain of E9.5d NTD mouse brain. Scale bars: 50μm. Data represent mean ± SEM from n = 3 biological replicate. Statistical significance was determined by ​​Wilcoxon test.
(D) Axin2 gRNAs cloned into vectors conveying puromycin resistance to allow for double selection after the lentiviral transduction of sv129 mESC. One cell clones obtained from two different gRNA combinations carrying the homozygous deletion and the same single cell clones that underwent no CRISPR system treatment.
(E) RT-qPCR data of gene expression in single cell clones carrying the homozygous deletion of Axin2 gRNA. Data represent mean ± SEM from n = 3 biological replicate. Statistical significance was determined by unpaired two-tailed Student’s t-test.
