# Supplementary material for: Excessive DNA Double‐Strand Breaks–Associated 3D Genome Reorganization Contributes to Neural Tube Defects with Folate Deficiency
Source: Adv Sci (Weinh). 2025 Sep 18;12(47):e10603. doi: 10.1002/advs.202410603 (PMC12713105; doi:10.1002/advs.202410603)
Supplement: Supplementary file 2 — Supplemental Table 1 [file ADVS-12-e10603-s004.docx]

Supplementary Table S1: Primers used for 3C assay

| Forward/  Reverse | Sequence(5'to3') | Category |
| --- | --- | --- |
| Forward | TCCCAAGGCCTGACACGATT | Chr17：13476685-13476878 |
| Reverse  Forward  Reverse  Forward  Reverse | CGGGGCCAAACAGCTAAGAA  CGCCAGTCTGTTCCTTGTCT  CGGCTGTCTTGCAAAAGTGG  CAGCACCTCATCACAGAGCA  CCGCAGTGAGATGCATTTCG | Chr17：13829624-13829882  Chr3：18045633-18046045  Chr3：18705125-18705322  Chr6：115887341-115887504  Chr6：116192610-116192744 |
