# Supplementary material for: Excessive DNA Double‐Strand Breaks–Associated 3D Genome Reorganization Contributes to Neural Tube Defects with Folate Deficiency
Source: Adv Sci (Weinh). 2025 Sep 18;12(47):e10603. doi: 10.1002/advs.202410603 (PMC12713105; doi:10.1002/advs.202410603)
Supplement: Supplementary file 4 — Supplemental Table 3 [file ADVS-12-e10603-s006.docx]

Supplementary Table S3: Primers used for Real Time RT-qPCR

| Name | Forward/  Reverse | Sequence(5'to3') |
| --- | --- | --- |
| GAPDH | Forward | TGACCTCAACTACATGGTCTACA |
|  | Reverse | CTTCCCATTCTCGGCCTTG |
| Ift122 | Forward | GCATTCTGAAGTACACGCACA |
|  | Reverse | TGCTCAGGAGACCATAACCCA |
| Cand2 | Forward | GGTGCCTTCTACATCTCCAGC |
|  | Reverse | CATCAGGTCACTGGTAGCCAT |
| Alox5 | Forward | GGGCTGTAGCGAGAAGCATC |
| Zeb1  Ascl1  Sox6  Axin2  Arhgap35  Brd1 | Reverse  Forward  Reverse  Forward  Reverse  Forward  Reverse  Forward  Reverse  Forward  Reverse  Forward  Reverse | CACGGTGACATCGTAGGAGT  ACCGCCGTCATTTATCCTGAG  CATCTGGTGTTCCGTTTTCATCA  TTCTCCGGTCTCGTCCTACTC  CCAGTTGGTAAAGTCCAGCAG  ACGTCTACCTCACCACATAAGC  CGGGGTTCCAAAAGTAACACT  AACCTATGCCCGTTTCCTCTA  GAGTGTAAAGACTTGGTCCACC  CCGCATCCCCACCTACAAC  GACTTCCCAATGCCGCACT  TTCTTCCCCGTGCAGTATTAAAC  CCCTTCGATTTCTATCTCCACCA |
