# Supplementary material for: Excessive DNA Double‐Strand Breaks–Associated 3D Genome Reorganization Contributes to Neural Tube Defects with Folate Deficiency
Source: Adv Sci (Weinh). 2025 Sep 18;12(47):e10603. doi: 10.1002/advs.202410603 (PMC12713105; doi:10.1002/advs.202410603)
Supplement: Supplementary file 7 — Supplemental Table 6 [file ADVS-12-e10603-s010.docx]

Supplementary Table S6: Primers used for PCR with indel validation

| Name | Forward/  Reverse | Sequence(5'to3') |
| --- | --- | --- |
| Ift122-Ko1 | Forward | TGAAGCATTTCAGCATCTCG |
|  | Reverse | AAGCCACACCGGAGGTTAAT |
| Ift122-Ko2 | Forward | AGAAAGGGAAAAGGGACCAA |
|  | Reverse | TTCTCATGTTGTCCCAGCAA |
| Ift122-Con | Forward | TCCCATAGCCAAGTGGAGAG |
| Zeb  Ascl1  Sox6  Axin2 | Reverse  Forward  Reverse  Forward  Reverse  Forward  Reverse  Forward  Reverse | GGCAAGTGGCTTTCTTTCTG  AGCCAAACATCCCCAGTCTT  TCAGTCTTCCTTTGGTCTGCT  TGTGGTTTTGTGCTTCGGAG  ATCTGCGCACCGTTTACATG  AGGCACAGACACATAGCTCA  AACACTGTGGGAGGAGGAAG  TCTGTCTTCCCAATGTCCCC  TCGTTTGATCCAGGCTAGCT |
