# Supplementary material for: Excessive DNA Double‐Strand Breaks–Associated 3D Genome Reorganization Contributes to Neural Tube Defects with Folate Deficiency
Source: Adv Sci (Weinh). 2025 Sep 18;12(47):e10603. doi: 10.1002/advs.202410603 (PMC12713105; doi:10.1002/advs.202410603)
Supplement: Supplementary file 8 — Supplemental Table 7 [file ADVS-12-e10603-s009.docx]

Supplementary Table S7: Clinical manifestations of normal fetus and NTDs fetus

| Sample type | No | Tissue | Gender | Gestational Weeks |
| --- | --- | --- | --- | --- |
| Normal | A1910 | Brain | female | 22 |
| Normal | A2715 | Brain | female | 36 |
| Normal | A1385 | Brain | female | 26 |
| Normal | A1581 | Brain | male | 21 |
| Normal | A1490 | brain and spine | male | 25 |
| Normal | A2570 | Brain | female | 22 |
| Normal | A2499 | Brain | male | 26 |
| Normal | A2542 | brain and spine | female | 25 |
| Normal | A2390 | Brain | male | 18 |
| Normal | A1462 | Brain | female | 15 |
| Normal | A2415 | Brain | male | 23 |
| Normal | A1753 | Brain | male | 16 |
| Normal | A2633 | Brain | female | 24 |
| Normal | A2664 | Brain | female | 22 |
| Normal | A2625 | Brain | female | 23 |
| Normal | A2550 | Brain | male | 24 |
| Normal | A2630 | Brain | female | 24 |
| Normal | A2853 | Brain | male | 27 |
| Normal | A2867 | brain and spine | male | 24 |
| Normal | A2628 | Brain | female | 25 |
| Normal | A2427 | Brain | female | 19 |
| Normal | A2430 | Brain | male | 18 |
| Normal | A2502 | Brain | female | 22 |
| Normal | A2928 | Brain | male | 20 |
| Normal | A1492 | Brain | female | 20 |
| Normal | A1815 | Brain | male | 20 |
| Normal | A1718 | Brain | female | 20 |
| Normal | A2125 | Brain | male | 22 |
| Normal | A2547 | Brain | female | 28 |
| Normal | A2755 | Brain | female | 18 |
| Normal | A2777 | Brain | male | 20 |
| Normal | A2557 | Brain | female | 18 |
| Normal | A2582 | Brain | male | 19 |
| Normal | A1799 | Brain | male | 16 |
| Normal | A2743 | Brain | male | 18 |
| Normal | A2776 | Brain | male | 16 |
| Normal | A2576 | Brain | female | 19 |
| Normal | A2497 | Brain | female | 15 |
| Normal | A1795 | Brain | female | 14 |
| Normal | A1631 | Brain | male | 18 |
| Normal | A1709 | Brain | female | 15 |
| Normal | A1733 | Brain | male | 17 |
| Normal | A1741 | Brain | female | 14 |
| Normal | A1770 | Brain | female | 20 |
| Normal | A1787 | Brain | female | 22 |
| Normal | A1789 | Brain | female | 22 |
| Normal | A1818 | Brain | female | 25 |
| Normal | A1914 | Brain | male | 21 |
| Normal | A1936 | Brain | male | 22 |
| Normal | A2575 | Brain | female | 18 |
| Normal | A2726 | Brain | female | 22 |
| Normal | A2750 | Brain | female | 18 |
| Normal | A2752 | Brain | male | 18 |
| Normal | A2584 | Brain | female | 17 |
| Normal | A2397 | Brain | male | 19 |
| Normal | A2671 | Brain | male | 20 |
| Normal | A2875 | Brain | female | 21 |
| Normal | A2889 | brain and spine | female | 24 |
| Normal | A2193 | Brain | female | 25 |
| Normal | A2709 | Brain | female | 21 |
| Normal | A2691 | Brain | male | 22 |
| Normal | A2162 | Brain | male | 21 |
| Normal | A2738 | Brain | female | 20 |
| Normal | A2888 | Brain | female | 23 |
| Normal | A2889 | Brain | female | 24 |
| Normal | A1416 | brain and spine | male | 20 |
| Normal | A2872 | Spine | female | 20 |
| Normal | A2611 | Brain | male | 30 |
| Normal | A2666 | Brain | female | 20 |
| Normal | A2643 | Brain | female | 20 |
| Normal | A1783 | Brain | male | 21 |
| Normal | A2694 | Brain | male | 18 |
| Normal | A2918 | Brain | male | 15 |
| Normal | A2753 | Brain | male | 18 |
| Normal | A2888 | Brain | female | 23 |
| Normal | A1490 | Brain | male | 25 |
| Normal | A2872 | Brain | female | 20 |
| Normal | A2542 | Brain | female | 25 |
| Normal | A2497 | Brain | female | 15 |
| Normal | A2920 | Brain | male | 18 |
| Normal | A2776 | Brain | male | 16 |
| Normal | A2867 | Brain | male | 24 |
| Normal | A2415 | Brain | male | 23 |
| Normal | A2397 | Brain | male | 19 |
| Normal | A2155 | Brain | female | 18 |
| Normal | A2734 | Brain | female | 18 |
| Normal | A2595 | Brain | female | 17 |
| Normal | A2336 | Brain | female | 16 |
| Normal | A2671 | Brain | male | 20 |
| Normal | A2666 | Brain | female | 20 |
| Normal | A1783 | Brain | male | 21 |
| Normal | A2694 | Brain | male | 18 |
| Normal | A2777 | Brain | male | 20 |
| Normal | A2928 | Brain | male | 20 |
| Normal | A2918 | Brain | male | 15 |
| Normal | A1855 | Brain | male | 20 |
| Normal | A2592 | Brain | female | 16 |
| Normal | A1611 | Brain | female | 39 |
| NTDs | A2515 | Brain | female | 20 |
| NTDs | A2545 | Brain | female | 33 |
| NTDs | A1589 | Brain | female | 27 |
| NTDs | A1520 | Brain | male | 22 |
| NTDs | A2620 | Brain | male | 16 |
| NTDs | A1529 | Brain | female | 21 |
| NTDs | A1345 | Brain | male | 19 |
| NTDs | A1272 | Brain | female | 23 |
| NTDs | A1676 | Brain | female | 24 |
| NTDs | A2190 | Brain | female | 19 |
| NTDs | A2289 | Brain | male | 16 |
| NTDs | A2364 | Brain | male | 21 |
| NTDs | A2495 | Brain | female | 15 |
| NTDs | A2618 | Brain | male | 18 |
| NTDs | A1446 | Brain | female | 20 |
| NTDs | A2248 | Brain | female | 26 |
| NTDs | A1447 | Brain | female | 24 |
| NTDs | A1504 | Brain | male | 22 |
| NTDs | A2037 | Brain | male | 20 |
| NTDs | A1677 | Brain | female | 21 |
| NTDs | A2760 | Brain | female | 34 |
| NTDs | A1508 | Brain | female | 33 |
| NTDs | A1606 | Brain | male | 35 |
| NTDs | A1819 | brain and spine | female | 32 |
| NTDs | A1594 | Brain | female | 31 |
| NTDs | A1648 | Brain | male | 22 |
| NTDs | A2007 | Brain | male | 20 |
| NTDs | A2425 | Brain | female | 26 |
| NTDs | A2132 | brain and spine | female | 25 |
| NTDs | A1920 | Brain | female | 25 |
| NTDs | A1419 | Brain | female | 32 |
| NTDs | A2851 | Brain | female | 34 |
| NTDs | A2850 | Brain | male | 31 |
| NTDs | A1938 | Brain | male | 21 |
| NTDs | A1895 | Brain | female | 25 |
| NTDs | A1869 | Brain | male | 39 |
| NTDs | A1380 | Brain | male | 31 |
| NTDs | A2423 | Brain | male | 40 |
| NTDs | A2857 | Brain | female | 22 |
| NTDs | A2067 | Brain | male | 21 |
| NTDs | A2061 | Brain | female | 20 |
| NTDs | A2412 | brain and spine | female | 20 |
| NTDs | A1895 | brain and spine | female | 25 |
| NTDs | A2189 | brain and spine | female | 15 |
| NTDs | A1649 | Brain | male | 18 |
| NTDs | A2223 | Brain | female | 24 |
| NTDs | A1859 | brain and spine | male | 16 |
| NTDs | A1515 | brain and spine | female | 18 |
| NTDs | A2188 | Brain | female | 16 |
| NTDs | A1748 | Brain | female | 18 |
| NTDs | A2856 | Brain | male | 19 |
| NTDs | A2138 | Brain | female | 24 |
| NTDs | A2158 | Brain | female | 37 |
| NTDs | A2198 | Brain | female | 20 |
| NTDs | A2228 | Brain | male | 20 |
| NTDs | A2242 | Brain | male | 31 |
| NTDs | A2480 | Brain | female | 30 |
| NTDs | A2548 | Brain | male | 24 |
| NTDs | A2601 | Brain | male | 25 |
| NTDs | A2710 | Brain | female | 16 |
| NTDs | A2761 | Brain | male | 22 |
| NTDs | A1484 | Brain | male | 21 |
| NTDs | A1658 | Brain | male | 28 |
| NTDs | A1701 | Brain | male | 22 |
| NTDs | A1743 | Brain | male | 35 |
| NTDs | A1887 | Brain | male | 28 |
| NTDs | A1900 | Brain | male | 38 |
| NTDs | A1419 | Spine | female | 32 |
| NTDs | A2280 | Brain | female | 21 |
| NTDs | A2271 | Brain | male | 40 |
| NTDs | A2448 | Brain | male | 20 |
| NTDs | A1677 | brain and spine | female | 21 |
| NTDs | A2731 | Brain | male | 23 |
| NTDs | A1525 | Brain | male | 24 |
| NTDs | A2237 | Brain | female | 24 |
| NTDs | A1546 | Brain | female | 25 |
| NTDs | A2225 | Brain | male | 24 |
| NTDs | A1933 | Brain | male | 22 |
| NTDs | A2446 | Brain | female | 20 |
| NTDs | A2454 | Brain | female | 23 |
| NTDs | A2678 | Brain | female | 24 |
| NTDs | A1768 | Brain | male | 25 |
| NTDs | A2455 | brain and spine | male | 20 |
| NTDs | A1534 | Brain | male | 30 |
| NTDs | A1374 | Brain | female | 20 |
| NTDs | A2238 | Brain | female | 20 |
| NTDs | A2686 | Brain | male | 21 |
| NTDs | A2101 | Brain | male | 18 |
| NTDs | A1542 | Brain | male | 19 |
| NTDs | A2060 | Brain | male | 20 |
| NTDs | A1503 | Brain | female | 26 |
| NTDs | A1838 | Brain | male | 15 |
| NTDs | A2446 | Brain | female | 20 |
| NTDs | A2454 | Brain | female | 23 |
| NTDs | A1768 | Brain | male | 25 |
| NTDs | A1649 | Brain | male | 18 |
| NTDs | A2223 | Brain | female | 24 |
| NTDs | A1748 | Brain | female | 18 |
| NTDs | A1586 | Brain | male | 25 |
| NTDs | A2188 | Brain | female | 16 |
| NTDs | A2279 | Brain | female | 16 |
| NTDs | A2448 | Brain | male | 20 |
| NTDs | A2237 | Brain | female | 24 |
| NTDs | A2455 | Brain | male | 20 |
| NTDs | A1534 | Brain | male | 30 |
| NTDs | A1374 | Brain | female | 20 |
| NTDs | A2238 | Brain | female | 20 |
| NTDs | A2686 | Brain | male | 21 |
| NTDs | A2101 | Brain | male | 18 |
| NTDs | A2060 | Brain | male | 20 |
| NTDs | A1838 | Brain | male | 15 |
|  |  |  |  |  |
