# Supplementary material for: Excessive DNA Double‐Strand Breaks–Associated 3D Genome Reorganization Contributes to Neural Tube Defects with Folate Deficiency
Source: Adv Sci (Weinh). 2025 Sep 18;12(47):e10603. doi: 10.1002/advs.202410603 (PMC12713105; doi:10.1002/advs.202410603)
Supplement: Supplementary file 9 — Supplemental Table 8 [file ADVS-12-e10603-s008.docx]

Supplementary Table S8: Oligonucleotides for gRNA cloning into LentiCRISPR

| Locus | Forward/  Reverse | Oligonucleotide sequence (5'to3') |
| --- | --- | --- |
| Ift122-Ko1 | Forward | CACCGAAAGGGGCTGAGGATACGTG |
|  | Reverse | AAACCACGTATCCTCAGCCCCTTTC |
| Ift122-Ko1 | Forward | CACCGAAACAACCCAATAAAACAGT |
|  | Reverse | AAACACTGTTTTATTGGGTTGTTTC |
| Ift122-Ko2 | Forward | CACCGTTGACACCTAAAGTAAACTG |
|  | Reverse | AAACCAGTTTACTTTAGGTGTCAAC |
| Ift122-Ko2 | Forward | CACCGTGTCCTTCACTACACCACAC |
|  | Reverse | AAACGTGTGGTGTAGTGAAGGACAC |
| Ift122-Con | Forward | CACCGATGCACTGTGTATTGAGACA |
|  | Reverse | AAACTGTCTCAATACACAGTGCATC |
| Ift122-Con  Zeb1-3742  Zeb1-959  Ascl1-3263  Ascl1-331  Sox6-dis3251  Sox6-dis973  Sox6-con2996  Sox6-con61  Axin2-1833  Axin2-31 | Forward  Reverse  Forward  Reverse  Forward  Reverse  Forward  Reverse  Forward  Reverse  Forward  Reverse  Forward  Reverse  Forward  Reverse  Forward  Reverse  Forward  Reverse  Forward  Reverse | CACCGCACAGTACCCAGTATCCGCG  AAACCGCGGATACTGGGTACTGTGC  CACCGTATAGATGGGTAAATAACAG  AAACCTGTTATTTACCCATCTATAC  CACCGTAGGAAAATCCATTAGCAAG  AAACCTTGCTAATGGATTTTCCTAC  CACCGGTAATCTTAAGAAAACGATG  AAACCATCGTTTTCTTAAGATTACC  CACCGAACAACATATATCAAATATG  AAACCATATTTGATATATGTTGTTC  CACCGGTGGCACCTTTAGAGGTCCG  AAACCGGACCTCTAAAGGTGCCACC  CACCGACCACACAATTAGTGAGCAG  AAACCTGCTCACTAATTGTGTGGTC  CACCGTAACTCCAGAGATTGCCAGA  AAACTCTGGCAATCTCTGGAGTTAC  CACCGACAGCAGAATGGACATGTCA  AAACTGACATGTCCATTCTGCTGTC  CACCGAGTCACCAATCTTCATCATG  AAACCATGATGAAGATTGGTGACTC  CACCGTATAATCCAAGCCAAGATGG  AAACCCATCTTGGCTTGGATTATAC |
|  |  |  |
