# Supplementary material for: Excessive DNA Double‐Strand Breaks–Associated 3D Genome Reorganization Contributes to Neural Tube Defects with Folate Deficiency
Source: Adv Sci (Weinh). 2025 Sep 18;12(47):e10603. doi: 10.1002/advs.202410603 (PMC12713105; doi:10.1002/advs.202410603)
Supplement: Supplementary file 10 — Supplemental Table 9 [file ADVS-12-e10603-s005.docx]

Supplementary Table S9: Embryonic phenotypes of mice treated with folate-deficient

food and MTX

| MTX  (mg kg^-1^) | Embryos  (n) | Normal  n (%) | Resorption  n (%) | NTDs  n (%) | Other malformations n (%) |
| --- | --- | --- | --- | --- | --- |
| 0 | 120 | 118 | 2 | 0 | 0 |
| 1.5 | 150 | 90 | 4 | 56 | 1^a^ |

Note: ^a^Craniofacial malformation
